# Iron overload in the tumor microenvironment induces CD8^+^ T cell ferroptosis and dysfunction

**DOI:** 10.1038/s41467-026-73379-4

**Published:** 2026-05-22

**Authors:** Zhenyu Lin, Huanpeng Chen, Yujing Ke, Hanyue Xiao, Chao Li, Zilong Wu, Huixin Gao, Nanqi Huang, Lijuan Lu, Peng Sun, Yingjie Bian

**Affiliations:** 1 Guangzhou National Laboratory, Guangzhou International Bio-Island, Guangzhou, China; 2 Zhongshan School of Medicine, Sun Yat-sen University, Guangzhou, China; 3 State Key Laboratory of Respiratory Disease, Guangzhou Institute of Respiratory Health, The First Affiliated Hospital of Guangzhou Medical University, Guangzhou, China; 4 Guangzhou Medical University, Guangzhou, China; 5 Department of Gastrointestinal Surgery, Union Hospital, Tongji Medical College, Huazhong University of Science and Technology, Wuhan, China; 6 Department of Clinical Laboratory, Guangzhou Women and Children Medical Center, Guangzhou Medical University, Guangzhou, China; 7 Department of Gastrointestinal Surgery, Guangdong Provincial Key Laboratory of Major Obstetric Diseases, Guangdong Provincial Clinical Research Center for Obstetrics and Gynecology, The Third Affiliated Hospital of Guangzhou Medical University, Guangzhou, China; 8 Department of Medical Oncology, The Third Affiliated Hospital of Sun Yat-sen University, Guangzhou, China; 9 State Key Laboratory of Oncology in South China, Collaborative Innovation Center for Cancer Medicine, Guangzhou, China; 10 Department of Pathology, Sun Yat-sen University Cancer Center, Guangzhou, China

**Keywords:** Cancer metabolism, Cancer microenvironment, Cancer immunotherapy, Tumour immunology, CD8-positive T cells

## Abstract

While iron homeostasis in cancer cells is well-established, its role in mediating crosstalk between tumors and CD8^+^ T cells within the tumor microenvironment (TME) remains largely elusive. In this study, we compare iron levels across primary tissues populated by CD8^+^ T cells. Contrary to the systemic iron deficiency commonly found in cancer patients, the TME exhibits marked iron enrichment compared to lymphatic fluid and peripheral blood, a phenomenon primarily attributed to tumor necrosis. However, this iron-overloaded TME is detrimental to CD8^+^ T cells, triggering their ferroptosis and dysfunction. Mechanistically, tumoral T cell receptor (TCR) hyperactivation and tumor-derived hepcidin cooperatively downregulate the iron exporter SLC40A1 in CD8^+^ T cells, leading to intracellular iron accumulation and ferroptosis. Both genetic restoration of SLC40A1 and iron chelation inhibit CD8^+^ T cell ferroptosis and restore their cytotoxic activity, thereby suppressing tumor growth. Finally, to enhance chimeric antigen receptor T (CAR-T) cell adaptability to the iron-overloaded TME, we engineer SLC40A1-overexpressing CAR-T cells. These engineered cells resist ferroptosis induced by the TME and elicit potent anti-tumor immunity.

## Abstract (web summary)

Ferroptosis is an iron-dependent form of cell death with important implications for cancer biology. Here, the authors report that tumor necrosis-induced iron overload in the tumor microenvironment (TME) drives CD8^+^ T cell dysfunction by promoting ferroptosis, proposing the design of CAR-T cells overexpressing SLC40A1 (an iron exporter) to resist ferroptosis in the TME.

## Introduction

Iron deficiency is a prevalent manifestation in cancer patients, contributing to anemia-associated symptoms such as impaired physical function, weakness, and fatigue^1–4^. Thereby, iron supplementation has been integrated into clinical strategies to manage iron deficiency among cancer patients^3,4^. However, the impact of iron therapy on tumor progression remains contentious. Prior clinical trials evaluating iron repletion therapies report no evidence of tumor induction or accelerated progression within limited observation periods, yet uncertainties persist regarding long-term risks^5–10^. Conversely, other studies suggest iron acts as a tumor promoter by satisfying the metabolic demands of malignant cells^11–13^. Cancer cells frequently reprogram iron metabolism genes to meet iron demands for energy and biosynthesis. Key molecular adaptations include the upregulation of *TFRC* (encoding transferrin receptor 1, TfR1), a critical mediator of iron import, and the suppression of *SLC40A1*, the primary transporter responsible for intracellular iron efflux^14,15^. Studies have shown that high *TFRC* expression is a hallmark of aggressive malignancies and is robustly associated with poor prognosis across multiple cancer types^16–18^. Concurrently, tumors exploit downregulation of *SLC40A1* to sequester intracellular iron, a strategy linked to enhanced proliferation and metastasis^19–21^. Although the impact of iron homeostasis on tumor cells has been extensively characterized, its role in tumor-infiltrating lymphocytes (TILs) remains largely elusive^14^.

Iron serves as an essential cofactor for fundamental cellular processes, including the tricarboxylic acid (TCA) cycle, iron-sulfur cluster assembly, and mitochondrial biogenesis^22,23^. Recently, iron homeostasis has been implicated in CD4^+^ T cell-mediated autoimmune diseases. In systemic lupus erythematosus (SLE) and experimental autoimmune encephalomyelitis (EAE), activated CD4^+^ T cells upregulate TfR1 to enhance iron uptake, thereby driving autoimmune pathology^24,25^. And, iron promotes T follicular helper (Tfh) cell differentiation via Fe^2+^-dependent ten-eleven translocation (TET) enzyme activation, and drives CD4^+^ T cells toward a proinflammatory phenotype by glycolysis reprogramming^26–29^. However, emerging evidence also reveals iron overload impairs Th1 differentiation and interferon-γ (IFN-γ) production, while inducing mitochondrial fragmentation in activated CD4^+^ T cells^28,30^. Notably, the immunomodulatory role of iron in CD8^+^ T cells remains poorly defined. Here, we investigate the influence of the iron-overloaded tumor microenvironment (TME) on tumor-infiltrating CD8^+^ T cells (CD8^+^ TILs) and uncover an iron-dependent immune evasion employed by tumors.

In this work, we first compare iron concentrations across primary tissues containing CD8^+^ T cells. Under physiological conditions, lymphatic fluid shows significantly lower iron than peripheral plasma, whereas the TME exhibits substantial iron enrichment despite systemic iron deficiency. Functional assessment reveals a threshold requirement for iron in CD8^+^ T cells. Iron deficiency limits proliferation but largely spares cytotoxicity, whereas supraphysiological concentrations consistently induce ferroptosis and dysfunction. Activated CD8^+^ T cells synchronously upregulate iron importers, storage proteins, and the exporter SLC40A1 to maintain homeostasis. However, chronic TCR stimulation suppresses SLC40A1 transcription and translation, and tumor-derived hepcidin further promotes SLC40A1 degradation, leading to iron overload and ferroptosis. Accordingly, both SLC40A1 restoration and iron chelation rescue CD8^+^ T cell function. Finally, we find that the iron-overloaded TME impairs CAR-T cell efficacy by inducing ferroptosis. To address this, CAR-T cells are engineered to overexpress *Slc40a1*, enabling them to resist this stress and exhibit potent anti-tumor activity. Collectively, our findings uncover ferroptosis induced by the iron-overloaded TME in CD8^+^ T cells as a mechanism of tumor immune evasion and establish *Slc40a1*-overexpressing CAR-T cell therapy as a promising strategy to counteract tumoral iron stress.

## Results

### Tumor necrosis-induced iron-overloaded TME correlates with CD8^+^ T cell exhaustion

**Fig. 1:**
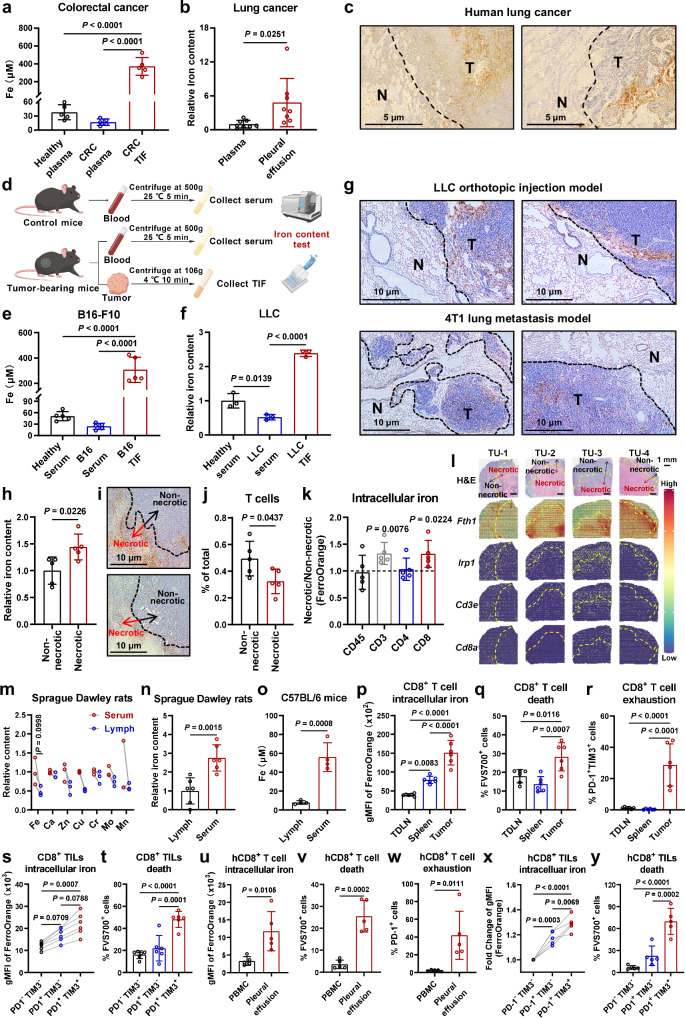
Tumor necrosis-induced iron-overloaded TME correlates with CD8^+^ T cell exhaustion. **a** Iron concentrations in plasma from healthy donors (*n* = 5 donors), and in plasma and paired tumor interstitial fluid (TIF) from colorectal cancer (CRC) patients (*n* = 5 patients). **b** Relative iron content in plasma and paired pleural effusion from lung cancer patients (*n* = 8 patients). **c** Representative Prussian blue staining on lung cancer sections from 2 patients. Brown deposits: iron enrichment. Dashed line: tumor (T) and normal (N). **d** Schematic of sample isolation. Created with BioGDP.com. Iron concentration (**e**, B16-F10, *n* = 5 mice) and relative iron content (**f**, LLC, *n* = 3 mice) in wild-type serum, and in serum and paired TIF from tumor-bearing mice. **g** Representative Prussian blue staining on lung sections from 4 mice. **h**–**k** Necrotic versus non-necrotic regions of LLC tumors (*n* = 5 mice). **h** Relative iron content. **i** Prussian blue staining. **j** Proportion of tumor-infiltrating T cells (TILs). **k** Ratio of FerroOrange-PE gMFI (intracellular iron) in CD45^+^, CD3^+^, CD4^+^ or CD8^+^ cells. **l** H&E and spatial transcriptomic maps of 4T1 tumors (*n* = 4 mice). **m,** **n** Relative content of metal elements (**m**, *n* = 3 rats) and iron (**n**, *n* = 6 rats) in serum and lymph from Sprague-Dawley rats. **o** Iron concentration in serum and lymph from C57BL/6 mice (*n* = 4 mice). CD8^+^ T cells from B16-F10 tumor-bearing mice (*n* = 6 mice): intracellular iron (**p**), cell death (**q**), and exhaustion (**r**) in CD8^+^ T cells from tumors, spleens, and tumor-draining lymph nodes (TDLN); intracellular iron (**s**) and cell death (**t**) in CD8^+^ TIL subpopulations. **u**–**y** Human CD8^+^ (hCD8^+^) T cells from lung cancer patients (*n* = 5 patients): intracellular iron (**u**), cell death (**v**), and exhaustion (**w**) in hCD8^+^ T cells isolated from pleural effusion and paired peripheral blood mononuclear cells (PBMC); intracellular iron (**x**) and cell death (**y**) in hCD8^+^ TIL subpopulations from pleural effusion. Data are mean ± SD. *P* values were calculated using one-way ANOVA (**a,** **e,** **f,** **p**–**t,** **x,** **y**); unpaired two-tailed t test (**b,** **h,** **j,** **k,** **n,** **o,** **u**–**w**); two-way ANOVA (**m**). Source data are provided as a Source Data file.

Iron deficiency is a common clinicopathological manifestation in cancer patients^1–4^, but comparative analyses of iron status across tissues remain limited. We collected peripheral plasma and tumor interstitial fluid (TIF) from colorectal cancer (CRC) patients. Inductively coupled plasma mass spectrometry (ICP-MS) analysis demonstrated that peripheral iron deficiency was observed in CRC patients. In contrast, iron was significantly enriched in the TME (Fig. 1a and Supplementary Fig. 1a). Furthermore, comparative analysis of interstitial fluid from cancerous and adjacent non-cancerous tissues revealed significantly enriched iron levels in the cancerous TIF (Supplementary Fig. 1b). Concordantly, malignant pleural effusion from lung cancer patients similarly exhibited iron enrichment relative to matched peripheral plasma (Fig. 1b). Prussian blue staining further confirmed significantly higher iron deposition in tumor regions versus adjacent non-tumor tissues (Fig. 1c and Supplementary Fig. 1c). In addition, we established multiple tumor-bearing murine models and collected peripheral serum and TIF (Fig. 1d). Notably, while peripheral serum showed iron deficiency in tumor-bearing mice as clinical patients, TIF exhibited significant iron enrichment (Fig. 1e, f and Supplementary Fig. 1d, e). Accordingly, Prussian blue staining across murine tumor tissues consistently demonstrated elevated iron abundance in tumor regions (Fig. 1g and Supplementary Fig. 1f). Collectively, these results indicate that despite peripheral iron deficiency, iron overload exists in the tumors.

To sustain rapid proliferation, tumor cells exhibit a high demand for iron. However, such aggressive growth often induces necrosis, thereby releasing intracellular contents into the extracellular environment due to compromised membrane integrity^31–34^. To understand whether necrosis is associated with iron enrichment in the TME, we collected necrotic and non-necrotic regions from LLC lung carcinoma (Supplementary Fig. 1g), and found that iron was significantly higher in necrotic regions (Fig. 1h). Prussian blue staining also corroborated this spatial iron accumulation (Fig. 1i). Accordingly, T cell infiltration of necrotic region was significantly reduced (Fig. 1j), while other types of immune cells, including macrophages (Mφ), dendritic cells (DCs), and natural killer (NK) cells, showed no significant differences (Supplementary Fig. 1h). We further compared the intracellular iron of T cells infiltrating necrotic versus non-necrotic regions, and found significant iron enrichment and cell death in CD8^+^ T cells within necrotic regions, whereas CD4^+^ T cells did not show such differences (Fig. 1k and Supplementary Fig. 1i). Additionally, to unambiguously identify iron metabolism in necrotic regions distinct from non-necrotic areas, we used spatial transcriptomic analyses of 4T1 tumours^35^. Unbiased clustering was able to differentiate between necrotic and non-necrotic regions, and these regions also separated clearly in the low-dimensional uniform manifold approximation and projection (UMAP) representation (Fig. 1l). Congruently, spatial transcriptomics data revealed significant enrichment of iron metabolism associated gene signatures in necrotic regions, whereas CD8^+^ T cell infiltration was markedly reduced compared to non-necrotic regions (Fig. 1l). However, the distribution of other immune cell types, including CD4^+^ T cells, Tregs, Mφ, DCs, and NK cells, showed no significant differences (Supplementary Fig. 1j). Besides, by inducing necrosis in vitro, we observed significant iron leakage in supernatants across different tumors (Supplementary Fig. 1k). Thus, these results indicate that tumor necrosis fosters iron overload in the TME, which is associated with impaired infiltration of T cells, particularly CD8^+^ T cells.

To further investigate how iron overload in the TME affects CD8^+^ T cells, we compared the iron landscape in the primary compartments where CD8^+^ T cells resided. By using ICP-MS, we quantified metal ions, including iron, in lymphatic fluid versus peripheral blood. Surprisingly, iron exhibited the largest magnitude of difference among all ions (Fig. 1m). Further analysis showed much lower iron in lymphatic fluid (7.95 ± 1.95 μM) than peripheral blood (56.00 ± 14.66 μM) (Fig. 1n, o). We also measured labile (redox-active) iron across different compartments and found that the TIF contains a much higher concentration of labile Fe^2+^ compared to lymph or serum (Supplementary Fig. 1l, m). To investigate whether environmental iron levels correlate with cellular iron status in T cells, we isolated CD8^+^ T cells from relevant tissues in tumor-bearing mice, including the tumor-draining lymph node (TDLN), spleen and tumor. We found tumor-infiltrating CD8^+^ T cells exhibited significantly higher intracellular iron than peripheral CD8^+^ T cells (Fig. 1p and Supplementary Fig. 1n), concomitant with increased cell death and exhaustion markers (Fig. 1q, r and Supplementary Fig. 1o). Notably, CD8^+^ T cells grouped by intracellular iron showed increased cell death with higher iron levels (Supplementary Fig. 1p). Thereafter, we further stratified CD8^+^ TILs by surface exhaustion markers into PD-1^-^TIM-3^-^, PD-1^+^TIM-3^-^, and PD-1^+^TIM-3^+^ subsets. The results revealed a stepwise increase in CD8^+^ T cell intracellular iron that paralleled the progression of exhaustion (Fig. 1s), and correlated with diminished cell viability (Fig. 1t). Similarly, analysis of clinical cancer patients confirmed that human CD8^+^ (hCD8^+^) TILs showed elevated iron compared to the peripheral counterparts (Fig. 1u), which significantly correlates with cell death and exhaustion (Fig. 1v, w). Moreover, stratifying hCD8^+^ TILs into exhaustion subsets confirmed that intracellular iron positively correlated with exhaustion severity and cell death (Fig. 1x, y). Taken together, these results reveal an association between iron overload in the TME and CD8^+^ T cell dysfunction, and suggest iron as a potential metabolic driver of T cell exhaustion.

### Iron overload drives CD8^+^ T cell dysfunction via ferroptosis

**Fig. 2:**
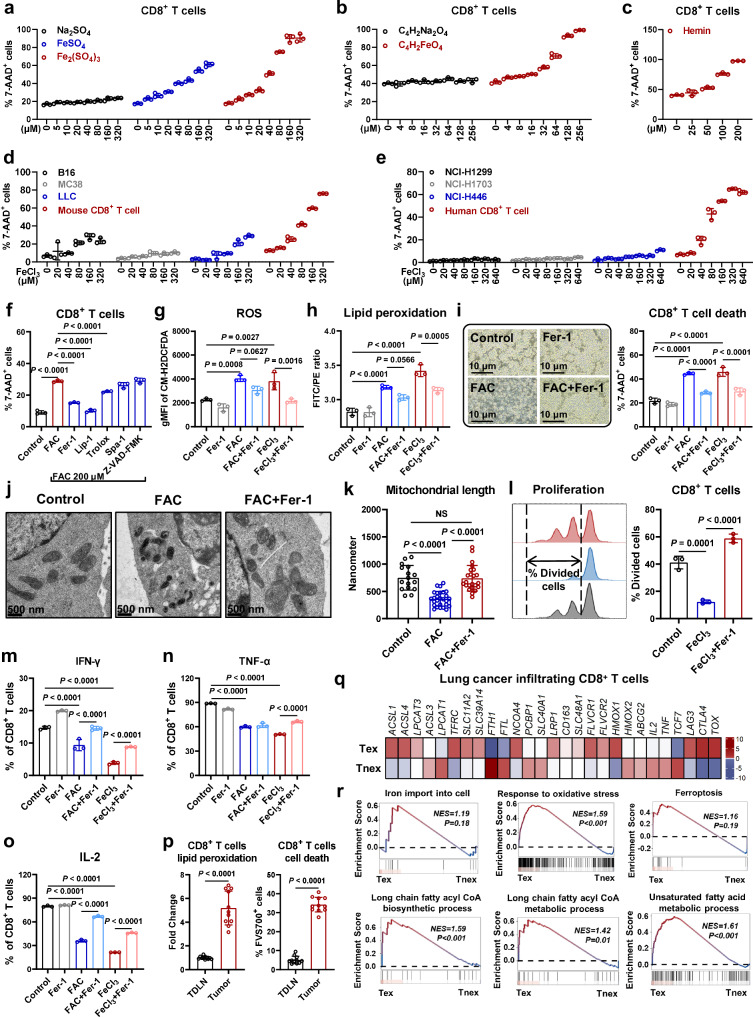
Iron overload drives CD8^+^ T cell dysfunction via ferroptosis. **a**–**c** Cell death of activated CD8^+^ T cells treated for 24 h with FeSO_4_, Fe_2_(SO_4_)_3_, Na_2_SO_4_ control; ferrous fumarate, sodium fumarate control; or hemin (*n* = 3 biologically independent samples). **d,** **e** Sensitivity of murine and human CD8^+^ T cells and tumor cell lines to FeCl_3_-induced cell death after 24 h treatment (*n* = 3 biologically independent samples). **f** Cell death of activated murine CD8^+^ T cells treated with ferric ammonium citrate (FAC, 200 μM) combined with Ferrostatin-1 (Fer-1, 1 μM), liproxstatin-1 (Lip-1, 1 μM), Trolox (20 μM), Spautin-1 (Spa-1, 5 μM), or Z-VAD-FMK (5 μM) for 48 h (*n* = 3 biologically independent samples). Activated murine CD8^+^ T cells were treated with FAC (200 μM) or FeCl_3_ (200 μM) ± Fer-1 (1 μM) for 48 h (*n* = 3 biologically independent samples). **g** Intracellular ROS. **h** Lipid peroxidation. **i** Bright-field morphology and cell death. Activated murine CD8^+^ T cells were treated with FAC (200 μM) ± Fer-1 (1 μM) for 48 h. **j** Transmission electron microscopy (TEM) images of mitochondria. **k** Long-axis length (Mitochondria counted: Control, *n* = 16; FAC, *n* = 27; FAC+Fer-1, *n* = 23). **l** Proliferation of CFSE-labeled activated CD8^+^ T cells treated with FeCl_3_ (200 μM) ± Fer-1 (1 μM) for 48 h (*n* = 3 biologically independent samples). **m**–**o** Cytokine production of activated CD8^+^ T cells treated with FAC (200 μM) or FeCl_3_ (200 μM) ± Fer-1 (1 μM) for 48 h (*n* = 3 biologically independent samples). **p** Lipid peroxidation and cell death in CD8^+^ T cells from B16-F10 tumors and matched TDLNs (*n* = 11 mice). **q,** **r** Analysis of single-cell RNA-sequencing data from human lung cancer, showing expression levels of genes related to ferroptosis, iron metabolism, and function across exhausted (Tex) and non-exhausted (Tnex) CD8^+^ T cell clusters and GSEA enrichment for ferroptosis and iron metabolism pathways in indicated clusters (*n* = 7 patients). Data are mean ± SD. *P* values were calculated using one-way ANOVA (**f**–**i,** **k**–**o**); unpaired two-tailed t test (**p**). Source data are provided as a Source Data file.

To delineate the mechanism by which iron overload compromised CD8^+^ T cell function, we used a serum-free T cell culture medium with iron deficiency (MT101), with an iron concentration significantly lower than that of physiological fluids and RPMI-1640 complete medium (Supplementary Fig. 2a). Under this condition, we found that iron supplementation at concentrations below the level in lymph significantly suppressed CD8^+^ T cell proliferation (Supplementary Fig. 2b), but did not substantially compromise viability, activation, or cytokine secretion (Supplementary Fig. 2c). However, supraphysiological iron concentrations promoted CD8^+^ T cell death and dysfunction in a dose-dependent manner, irrespective of iron preparations (Fig. 2a–c and Supplementary Fig. 2d, e). Conversely, tumor cells exhibited higher iron tolerance than CD8^+^ T cells (Fig. 2d and Supplementary Fig. 2f, g). These findings were further validated in primary human CD8^+^ T and tumor cells (Fig. 2e and Supplementary Fig. 2h, i). Thus, the above results demonstrate a potential threshold for iron requirement in CD8^+^ T cells, and supraphysiological iron induces CD8^+^ T cell death.

Ferroptosis is a programmed cell death characterized by the accumulation of lipid peroxides and the dependence on iron^36–38^. To determine whether the iron-overloaded TME induced CD8^+^ T cell ferroptosis, we treated CD8^+^ T cells with iron and cell death inhibitors. The results showed ferroptosis inhibitors, including ferrostatin-1 (Fer-1) and liproxstatin-1 (Lip-1), significantly attenuated iron-induced CD8^+^ T cell death, whereas autophagy or apoptosis inhibitors were ineffective (Fig. 2f and Supplementary Fig. 2j, k). Further, treatment of CD8^+^ T cells with diverse iron preparations consistently demonstrated that iron overload significantly triggered lipid peroxidation and induced ferroptosis (Fig. 2g–k). Notably, Fer-1 markedly suppressed CD8^+^ T cell ROS accumulation (Fig. 2g), lipid peroxidation (Fig. 2h), cell death (Fig. 2i) and mitochondrial shrinkage (Fig. 2j, k). Phenotypic analysis of CD8^+^ T cells revealed that ferroptosis inhibitors substantially rescued iron overload-mediated proliferation suppression (Fig. 2l), activation inhibition (Supplementary Fig. 2l, m), and cytokine impairment, including IFN-γ, tumor necrosis factor-α (TNF-α) and interleukin-2 (IL-2) (Fig. 2m–o). Apoptosis is a well-established cell death pathway in T cells. To further clarify the impact of iron overload on apoptosis, we assessed caspase-3 cleavage and found that while baseline apoptosis was detectable, iron treatment did not further promote apoptosis (Supplementary Fig. 2n). Consistently, treatment with the ferroptosis inhibitor Fer-1 prevented iron-induced cell death and dysfunction, whereas the apoptosis inhibitor Z-VAD-FMK had no such effect (Supplementary Fig. 2o, p). Furthermore, to assess whether tumors induce CD8^+^ T cell ferroptosis in vivo, we isolated CD8^+^ T cells both from tumors and matched TDLNs. The results showed that CD8^+^ TILs exhibited significantly elevated lipid peroxidation and cell death (Fig. 2p), indicating CD8^+^ T cell ferroptosis occurred intratumorally.

Next, to better understand the differential sensitivity of CD8^+^ T cells and tumor cells to ferroptosis induced by iron overload, we performed a comparative analysis of ferroptosis resistance-related molecules expression in human and murine cell lines spanning multiple cancer types and CD8^+^ T cells. The results showed that tumor cells generally upregulated multiple ferroptosis protective genes, including GPX4, FSP1, ferritin, and ACSL3, whereas CD8^+^ T cells exhibited substantially lower expression of these molecules (Supplementary Fig. 3a–g). Consistently, treatment with ferroptosis inducers demonstrated that CD8^+^ T cells were more susceptible to ferroptosis than tumor cells (Supplementary Fig. 3h–k). Furthermore, comparison of ferroptosis phenotypes in necrotic versus non-necrotic regions showed that CD8^+^ T cells in necrotic regions exhibited significantly enhanced ferroptosis and low GPX4 expression, whereas tumor cells in these regions markedly upregulated GPX4 to resist ferroptosis (Supplementary Fig. 3l–n). Thus, our data indicate that iron overload induces CD8^+^ T cell ferroptosis and dysfunction.

To correlate these findings with clinical data, we analyzed single-cell RNA sequencing (scRNA-seq) data from human cancers. Analysis further revealed that CD8^+^ T cells expressed lower levels of ferroptosis suppressor genes (*GPX4, AIFM2, SLC7A11, ACSL3*) but higher levels of the ferroptosis-promoting gene *ACSL4* compared to malignant cells (Supplementary Fig. 3o)^39–47^. Then, we analyzed scRNA-seq data from human cancers^48,49^, and stratified CD8^+^ TILs into exhausted and non-exhausted subsets (Fig. 2q and Supplementary Fig. 3p). Our analysis revealed significant enrichment of genes associated with polyunsaturated phospholipid metabolism (*e.g. ACSL4, LPCAT3*) and iron import (*e.g. TFRC, SLC11A2, SLC39A14*) within exhausted CD8^+^ T cells (Fig. 2q and Supplementary Fig. 3p). Conversely, genes involved in monounsaturated fatty acid metabolism *(e.g. ACSL3, LPCAT1*) and iron export (*SLC40A1*) were markedly downregulated (Fig. 2q and Supplementary Fig. 3p). Further gene set enrichment analysis (GSEA) showed robustly enriched pathways related to ferroptosis, iron import, oxidative stress, long chain fatty acyl CoA biosynthetic/metabolic process, and unsaturated fatty acid metabolism within the exhausted CD8^+^ T cell (Fig. 2r and Supplementary Fig. 3q). Collectively, these results demonstrate that iron overload in the TME drives CD8^+^ T cell exhaustion via ferroptosis.

### SLC40A1 suppression causes iron overload and ferroptosis in CD8^+^ TILs

**Fig. 3:**
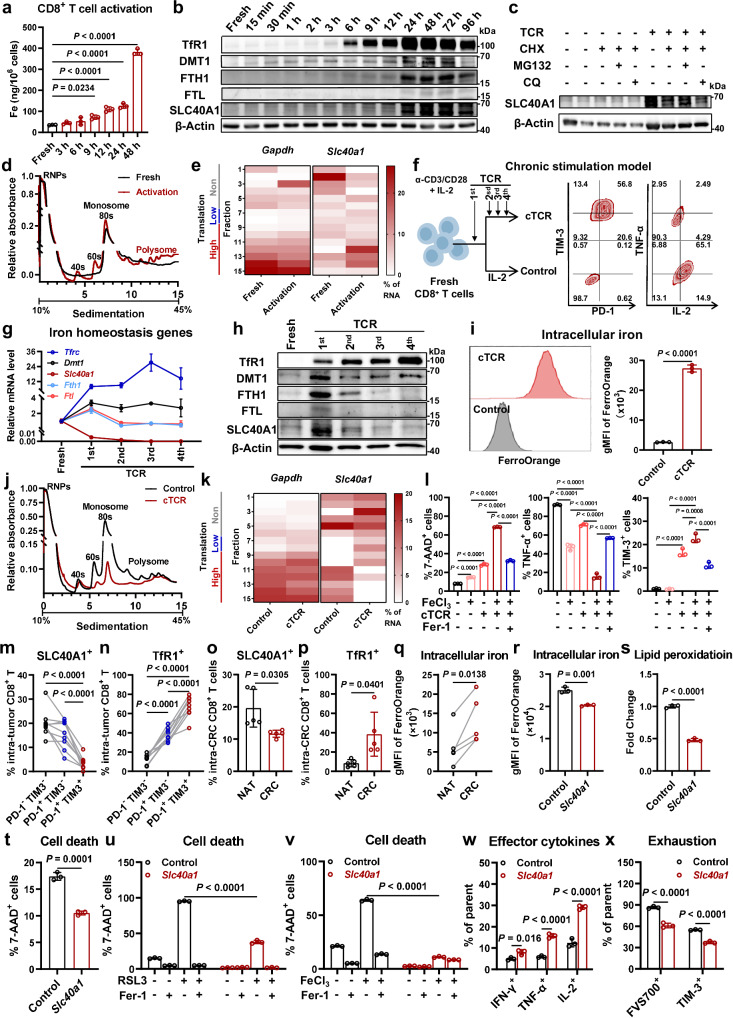
SLC40A1 suppression causes iron overload and ferroptosis in CD8^+^ TILs. **a,** **b** Intracellular total iron and protein expression of iron metabolism regulators during CD8^+^ T cell activation (*n* = 3 biologically independent samples). **c** Representative western blot (*n* = 3 independent experiments) of SLC40A1 protein stability in CD8^+^ T cells ± TCR stimulation. Activated and IL-7-maintained naïve cells were treated with cycloheximide (CHX, 100 μM) ± MG132 (10 μM) or chloroquine (CQ, 25 μM) for 6 h. **d** Representative polysome profiling (*n* = 3 independent experiments) of activated and naïve CD8^+^ T cells. Relative absorbance (260 nm) showing ribosomal subunits (40S, 60S), monosomes (80S), and polysomes. **e** Translational efficiency of *Slc40a1* mRNA, assessed by qRT-PCR of RNA from fractions in (**d**) (*n* = 3 biologically independent samples). **f**–**i** Iron metabolism of CD8^+^ T cell during chronic TCR (cTCR) stimulation (*n* = 3 biologically independent samples). **f** Schematic created with BioGDP.com. **g,** **h** mRNA and protein expression of iron metabolism regulators. **i** Intracellular iron. **j** Representative polysome profiling (*n* = 3 independent experiments) of control and cTCR-stimulated CD8^+^ T cells. **k** Translational efficiency of *Slc40a1* mRNA (*n* = 3 biologically independent samples). **l** Cell death, TNF-α secretion, and TIM-3 expression of CD8^+^ T cells treated with FeCl_3_ (100 μM) ± Fer-1 (1 μM) during cTCR stimulation (*n* = 3 biologically independent samples). **m,** **n** Expression of SLC40A1 and TfR1 in CD8^+^ TILs from B16-F10 model, stratified by exhaustion status (*n* = 9 mice). **o**–**q** Expression of SLC40A1, TfR1, and intracellular iron in CD8^+^ TILs from CRC tumors and paired adjacent normal tissues (NAT) (*n* = 5 patients). **r**–**x** Genetic restoration of *Slc40a1* in CD8^+^ T cells (*n* = 3 biologically independent samples). **r** Intracellular iron. **s** Lipid peroxidation. **t** Cell death. **u,** **v** Cell death after 24 h treatment with RSL-3 (50 nM) or FeCl_3_ (200 μM) ± Fer-1 (1 μM). **w** Effector cytokines. **x** Cell death and TIM-3 expression after cTCR stimulation. Data are mean ± SD. *P* values were calculated using one-way ANOVA (**a,** **l**–**n**); unpaired two-tailed t test (**i,** **r**–**t**); paired two-tailed t test (**o**–**q**); two-way ANOVA (**u**–**x**). Source data are provided as a Source Data file.

T cell exhaustion is canonically defined as a state of hypofunction driven by chronic T cell receptor (cTCR) stimulation. To investigate how the iron-overloaded TME disrupts CD8^+^ T cell iron homeostasis and triggers ferroptosis, we first analyzed iron homeostasis genes during CD8^+^ T cell activation. The results showed that the intracellular iron of CD8^+^ T cells gradually increased after activation (Fig. 3a and Supplementary Fig. 4a). Concurrently, genes for iron acquisition, storage, and utilization were significantly upregulated both at mRNA and protein levels (Fig. 3b and Supplementary Fig. 4b–e). However, mRNA of the iron efflux gene *Slc40a1* was downregulated (Supplementary Fig. 4f), while its protein level was significantly enriched (Fig. 3b). To determine if TCR activation enhanced SLC40A1 stability, we treated activated CD8^+^ T cells with the protein synthesis inhibitor cycloheximide (CHX). Although TCR activation robustly upregulated SLC40A1, CHX treatment revealed no increase in its stability (Fig. 3c). Furthermore, consistent with prior reports^50–52^, the proteasome inhibitor MG132, but not the lysosome inhibitor chloroquine (CQ), significantly delayed SLC40A1 degradation (Fig. 3c). Upregulation of protein levels is also attributable to enhancement in its translation efficiency. To investigate the regulatory mechanisms governing SLC40A1 expression upon TCR activation, we employed polysome profiling^53–55^. The result revealed that TCR activation induces a broad enhancement of mRNA translation, as evidenced by a global increase in polysome-associated mRNAs (Fig. 3d). Within this coordinated activation, *Slc40a1* mRNA was specifically and efficiently recruited to polysomes, demonstrating a significant increase in its ribosome occupancy and translational efficiency (Fig. 3e). Collectively, these data indicate that enhanced mRNA translation following TCR activation increases the abundance of SLC40A1 protein in CD8^+^ T cells to maintain iron homeostasis.

Next, to explore changes in iron homeostasis genes during CD8^+^ T cell dysfunction we utilized an in vitro chronic stimulation model (Fig. 3f). Results showed that cTCR activation led to further upregulation of iron acquisition genes, while mRNA levels of iron storage were largely maintained (Fig. 3g). Notably, the iron exporter SLC40A1 exhibited further downregulation at the RNA level, and its protein decreased significantly with cTCR stimulation (Fig. 3g, h and Supplementary Fig. 4g). Therefore, dysregulation of iron homeostasis genes ultimately results in intracellular iron overload in cTCR stimulated CD8^+^ T cells compared to control cells (Fig. 3i and Supplementary Fig. 4h). To investigate if translational regulation underlies the loss of SLC40A1, we compared *Slc40a1* translation efficiency in control and cTCR stimulated CD8^+^ T cells. Polysome profiling revealed a marked suppression of global mRNA translation (Fig. 3j) as well as a significant reduction in the ribosome occupancy of *Slc40a1* mRNA (Fig. 3k) upon cTCR stimulation in CD8^+^ T cells. Thus, during cTCR stimulation, impaired iron efflux culminates in intracellular iron overload and ferroptosis. To determine whether TCR signals are required for CD8^+^ T cell ferroptosis under iron-overloaded conditions, we examined CD8^+^ T cell ferroptosis with or without TCR stimulation. Notably, elevated iron levels alone were sufficient to trigger ferroptosis even in the absence of TCR engagement, whereas chronic TCR stimulation markedly exacerbated cell death (Supplementary Fig. 4i, j). Correspondingly, upon cTCR engagement, the ferroptosis inhibitor Fer-1 significantly suppressed CD8^+^ T cell death and dysfunction, while restoring effector cytokine expression, especially under iron-overloaded conditions (Fig. 3l).

Consistent with these findings, we further validated the results in primary human CD8^+^ T cells. Human CD8^+^ T cell activation consistently showed concurrent reduction of *SLC40A1* mRNA and elevation in its protein expression (Supplementary Fig. 4k–m), indicating a post-transcriptional regulation. However, upon cTCR stimulation, human CD8^+^ T cells showed further upregulation of *TFRC* but downregulation of *SLC40A1* (Supplementary Fig. 4n, o). This impairment in iron homeostasis led to intracellular iron accumulation (Supplementary Fig. 4p, q), consequently inducing ferroptosis (Supplementary Fig. 4r). Critically, this process was exacerbated by increasing iron concentrations (Supplementary Fig. 4s, t). Taken together, these results demonstrate that the suppression of SLC40A1 at both the transcriptional and translational levels is a key contributor to intracellular iron overload and ferroptosis during chronic TCR stimulation.

To validate these findings in vivo, we conducted three studies. First, we isolated CD8^+^ TILs from tumor-bearing mice, revealing significantly decreased SLC40A1 (Fig. 3m) and upregulated TfR1 (Fig. 3n). Second, analysis of CD8^+^ TILs from CRC patients demonstrated markedly reduced SLC40A1 compared to CD8^+^ T cells in the adjacent normal tissue (NAT) (Fig. 3o), concomitant with increased TfR1 and iron overload (Fig. 3p, q). And, within CD8^+^ TILs from CRC patients, PD-1^+^ exhausted subsets exhibited even lower SLC40A1 and higher TfR1, alongside higher iron accumulation relative to PD-1^−^ effector counterparts (Supplementary Fig. 4u). Last, scRNA-seq of human pan-cancer CD8^+^ TILs^56^ confirmed that exhausted cells displayed reduced *SLC40A1* and elevated *TFRC* compared to effector cells (Supplementary Fig. 4v), indicating iron dysregulation as a conserved hallmark of CD8^+^ T cell exhaustion in vivo. Notably, we further stratified CD8^+^ TILs into *SLC40A1*^high^ and *SLC40A1*^low^ expression groups. Gene set variation analysis (GSVA) of pan-cancers^57–61^ demonstrated significant enrichment of ferroptosis, iron import, oxidative stress and unsaturated fatty acid metabolic pathways in *SLC40A1*^low^-expression CD8^+^ TILs (Supplementary Fig. 4w).

Finally, to better validate whether downregulation of SLC40A1 induces ferroptosis and functional impairment in CD8^+^ T cells, we performed a rescue experiment. Genetic restoration of *Slc40a1* expression in T cells potentiated ferroptosis resistance under the iron overload conditions (Fig. 3r–v), conferred enhanced T cell effector cytokines, and decreased exhaustion (Fig. 3w, x). Collectively, TCR hyperactivation during tumor-induced CD8^+^ T cell dysfunction drives sustained repression of iron homeostasis genes, particularly the exporter SLC40A1, leading to cytotoxic iron accumulation, ferroptosis, and functional impairment.

### Cancer upregulates hepcidin to suppress SLC40A1 in CD8^+^ T cells

**Fig. 4:**
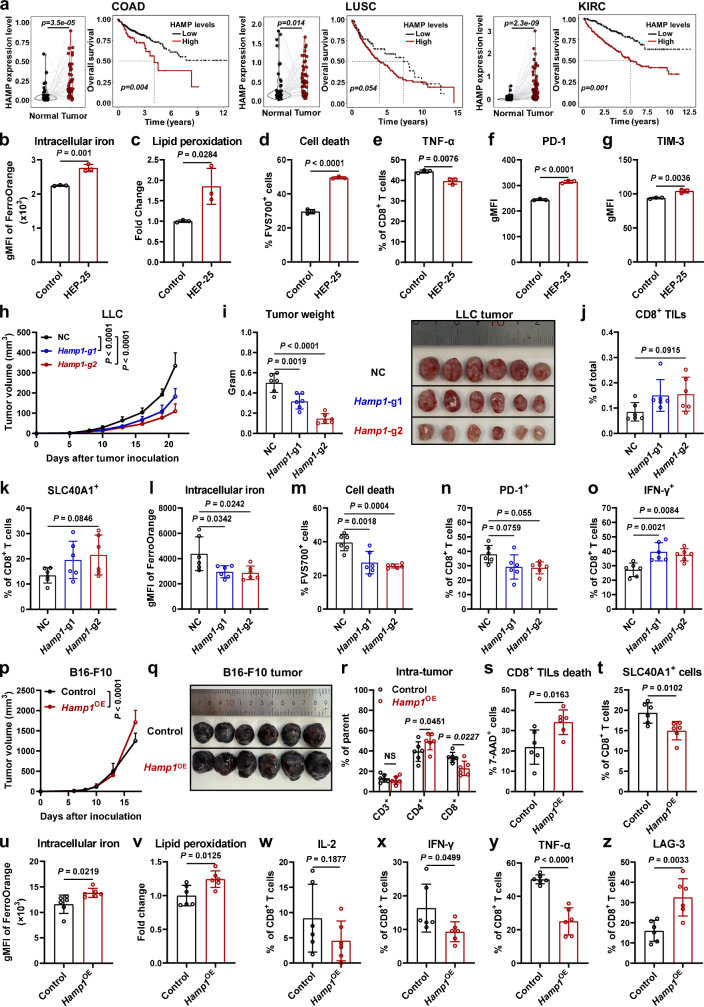
Cancer upregulates hepcidin to suppress SLC40A1 in CD8^+^ T cells. **a** *HAMP* mRNA expression in tumor versus adjacent normal tissues (Normal) from the TCGA database for colon adenocarcinoma (COAD, *n* = 41 patients), lung squamous cell carcinoma (LUSC, *n* = 51 patients), and kidney renal clear cell carcinoma (KIRC, *n* = 72 patients) (left panels). Kaplan-Meier survival curves of patients stratified by high versus low *HAMP* expression levels (right panels). Statistical significance was determined by the log-rank test. **b**–**g** Intracellular iron, lipid peroxidation, cell death, TNF secretion, and expression of exhaustion markers PD-1 and TIM-3 in human CD8^+^ T cells treated with Hepcidin-25 (HEP-25, 1 μM) during chronic TCR stimulation (*n* = 3 biologically independent samples). **h**–**o** Hepcidin (*Hamp1*) knockout suppresses LLC tumor progression in vivo (*n* = 6 mice). Control or *Hamp1*-knockout LLC cells (5 × 10^5^) were injected subcutaneously into C57BL/6 mice. **h,** **i** Tumor growth curves, weights, and images. **j** Proportions of CD8^+^ TILs. **k**–**o** SLC40A1 expression, intracellular iron, cell death, expression of exhaustion marker PD-1, and IFN-γ secretion of CD8^+^ TILs. **p**–**z** *Hamp1* overexpression promotes B16-F10 tumor progression in vivo (*n* = 6 mice). Control or *Hamp1*^OE^ B16-F10 cells (2 × 10^5^) were injected subcutaneously into C57BL/6 mice. **p,** **q** Tumor growth curves and images. **r** Proportions of tumor-infiltrating CD3^+^, CD4^+^, and CD8^+^ T cells. **s**–**z** Cell death, SLC40A1 expression level, intracellular iron, lipid peroxidation, secretion of cytokines IL-2, IFN-γ and TNF-α, and expression of exhaustion marker LAG-3 of CD8^+^ TILs. Data are mean ± SD. *P* values were calculated using unpaired two-tailed t test (**b**–**g,** **s**–**z**); two-way ANOVA (**h,** **p,** **r**); one-way ANOVA (**i**–**o**). Source data are provided as a Source Data file.

Hepcidin, the master regulator of systemic iron homeostasis, binds to and triggers the degradation of SLC40A1, thereby inhibiting cellular iron export^50,51,62^. Evidence indicated that tumor cells upregulate hepcidin to sequester iron, thereby promoting tumor survival and metastasis^63,64^. However, the impact of tumor-derived hepcidin on CD8^+^ T cell immunity remains undefined. Analysis of TCGA data reveals significant upregulation of hepcidin expression across multiple solid tumors (Fig. 4a and Supplementary Fig. 5a), which correlates significantly with poorer patient survival (Fig. 4a). To investigate whether tumor-derived hepcidin disrupts CD8^+^ T cell iron homeostasis and induces ferroptosis-related exhaustion, we designed complementary experimental approaches both in vitro and in vivo.

First, human CD8^+^ T cells were treated with bioactive hepcidin (HEP-25). HEP-25 significantly downregulated SLC40A1 (Supplementary Fig. 5b), which was inhibited by MG132 but not by CQ (Supplementary Fig. 5c). Furthermore, we observed that HEP-25 alone treatment slightly promoted human CD8^+^ T cell intracellular iron accumulation (Supplementary Fig. 5d), leading to enhanced lipid peroxidation and subsequent ferroptosis (Supplementary Fig. 5e, f). Correspondingly, inhibition using Fer-1 attenuated hepcidin-induced CD8^+^ T cell death (Supplementary Fig. 5f). However, within the chronic TCR stimulation model (Fig. 3f), HEP-25 supplementation in human CD8^+^ T cells markedly increased intracellular iron accumulation compared to untreated controls (Fig. 4b). This was accompanied by significantly elevated levels of lipid peroxidation and ferroptosis (Fig. 4c, d). Functional assessment further revealed that hepcidin promoted CD8^+^ T cell dysfunction, as evidenced by significantly impaired secretion of effector molecules and enhanced exhaustion markers (Fig. 4e–g). Similarly, treatment of murine CD8^+^ T cells with mouse hepcidin (Hep-1) resulted in a dose-dependent degradation of SLC40A1 protein (Supplementary Fig. 5g) and induced cell death and functional impairment in murine T cells (Supplementary Fig. 5h, i). These results demonstrate that hepcidin induces iron overload in CD8^+^ T cells via SLC40A1 suppression, driving ferroptosis and dysfunction in vitro.

To test the contributions of tumor-derived hepcidin in vivo, we endogenously knocked out hepcidin in LLC lung carcinoma and B16-F10 melanoma models (Supplementary Fig. 5j). Tumor hepcidin knockout significantly suppressed LLC tumor growth (Fig. 4h, i). Analysis of the tumor immune infiltrate revealed an increase in CD8^+^ T cell accumulation in hepcidin-knockout tumors (Fig. 4j). Furthermore, compared to those from control tumors, these CD8^+^ T cells exhibited higher SLC40A1 expression, reduced ferroptosis markers, and enhanced functional capacity (Fig. 4k–o). Similar results were observed in the B16-F10 melanoma model, where hepcidin knockout suppressed tumor growth and enhanced CD8^+^ T cell accumulation (Supplementary Fig. 5k–m).

Besides, we established tumor-bearing mouse models with tumor-specific hepcidin overexpression using LLC lung carcinoma and B16-F10 melanoma cells (Supplementary Fig. 5n). In vitro growth and proliferation assays confirmed that hepcidin overexpression did not intrinsically affect tumor cell viability (Supplementary Fig. 5o). Notably, LLC cells overexpressing hepcidin exhibited significantly accelerated tumor growth in immunocompetent mice (Supplementary Fig. 5p). Analysis of immune infiltration revealed that tumor-derived hepcidin had minimal impact on systemic immunity (Supplementary Fig. 5q, r), but profoundly suppressed the infiltration and viability of CD8^+^ TILs (Supplementary Fig. 5s, t). Similarly, hepcidin-overexpressing B16-F10 tumors demonstrated enhanced growth in immunocompetent mice (Fig. 4p, q). And hepcidin-overexpression significantly reduced infiltration and survival of CD8^+^ TILs (Fig. 4r, s), but with negligible effects on CD8^+^ T cells in TDLN (Supplementary Fig. 5u, v). Mechanistically, we found hepcidin overexpression within tumors down-regulated SLC40A1 expression on CD8^+^ TILs (Fig. 4t). This resulted in intracellular iron overload and significantly increased lipid ROS production of CD8^+^ TILs (Fig. 4u, v). Consequently, hepcidin-induced ferroptosis drove functional impairment of CD8^+^ T cells, characterized by diminished effector molecule secretion and the exhausted phenotypes (Fig. 4w–z). Notably, this SLC40A1 downregulation and iron dysregulation were absent in CD8^+^ T cells from peripheral lymphoid organs (Supplementary Fig. 5w–y).

In summary, tumors exploit hepcidin upregulation to degrade SLC40A1 expression in CD8^+^ TILs. This disrupts intracellular iron homeostasis of CD8^+^ T cells in the TME, ultimately inducing CD8^+^ T cell ferroptosis and exhaustion.

### Iron orchestration reprograms CD8^+^ T cell anti-tumor immunity

**Fig. 5:**
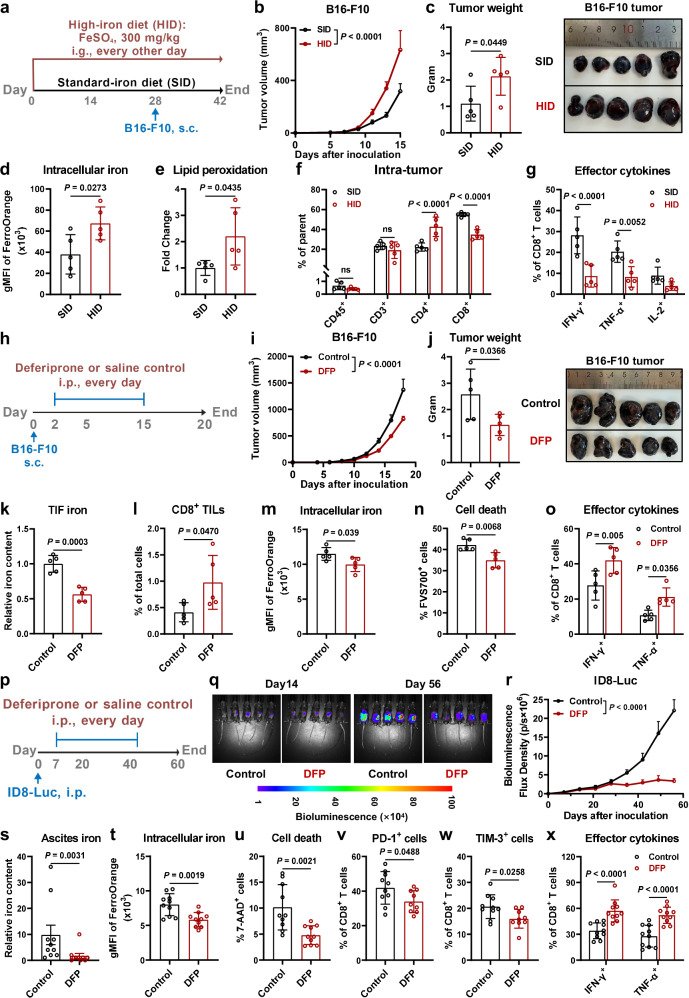
Iron orchestration reprograms CD8^+^ T cell anti-tumor immunity. High-iron diet (HID) promotes B16-F10 tumor progression (*n* = 5 mice). **a** Schematic. C57BL/6 mice in the HID group received FeSO_4_ (300mg/kg in ultrapure water, oral gavage every other day for 4 weeks); the standard-iron diet (SID) group received an equal volume of ultrapure water. On day 28, mice were subcutaneously inoculated with 2 × 10^5^ B16-F10 cells, and the gavage regimen was maintained until endpoint. **b, c** Tumor growth curves, weights and images. **d,** **e** Intracellular iron and lipid peroxidation in CD8^+^ TILs. **f** Proportions of tumor-infiltrating CD45^+^, CD3^+^, CD4^+^, and CD8^+^ cells. **g** Function of CD8^+^ TILs. **h**–**o** Deferiprone (DFP) therapy suppresses B16-F10 tumor growth (*n* = 5 mice). **h** Schematic. Mice were subcutaneously inoculated with 2 × 10^5^ B16-F10 cells on day 0. Treatment with DFP (150 mg/kg in saline) or vehicle control via intraperitoneal injection was initiated on day 2 and administered daily for two weeks, followed by a one-week withdrawal. **i,** **j** Tumor growth curves, weights and images. **k** Relative iron content in TIF. **l** Proportion of CD8^+^ TILs. **m**–**o** Intracellular iron, cell death, and effector function in CD8^+^ TILs. **p**–**x** DFP therapy suppresses ID8 tumor growth (*n* = 10 mice). **p** Schematic. Mice were intraperitoneally inoculated with 1.5 × 10^6^ ID8-luc cells on day 0. Treatment with DFP (150 mg/kg in saline) or vehicle control via intraperitoneal injection was initiated on day 7 and administered daily for 5 weeks, followed by an 18-day withdrawal. **q** Bioluminescence images of tumors on day 14 and day 56. **r** Tumor burden. **s** Relative iron content in ascites. **t**–**x** Intracellular iron, cell death, exhaustion markers expression, and cytokine production of CD8^+^ T cells from ascites. Data are mean ± SD except **s** (mean ± SEM). *P* values were calculated using two-way ANOVA (**b,** **f,** **g,** **i,** **o,** **r,** **x**); unpaired two-tailed t test (**c**–**e,** **j**–**n,** **t**–**w**); two-tailed Mann-Whitney U test (**s**). Source data are provided as a Source Data file.

To determine the potential anti-tumor effects of targeting iron accumulation in tumors, we first evaluated whether the iron supplementation may influence tumor progression. Mice received oral iron supplements every other day (Fig. 5a). Although the high-iron diet (HID) did not affect body weight (Supplementary Fig. 6a), it significantly promoted B16-F10 tumor growth in immunocompetent C57BL/6 mice compared to the standard iron diet (SID) (Fig. 5b, c). In contrast, HID treatment did not accelerate tumor growth in T cell-deficient nude mice, indicating that HID-mediated tumor promotion is T cell-dependent (Supplementary Fig. 6b–d). Flow cytometric analysis revealed that HID induced significant iron accumulation (Fig. 5d) and lipid peroxidation (Fig. 5e) in CD8^+^ T cells, whereas other immune cells, including macrophages, DCs, and NK cells, were minimally affected (Supplementary Fig. 6e). Consequently, the proportion of CD8^+^ T cells among TILs was markedly reduced (Fig. 5f), whereas their frequency in peripheral TDLNs remained unaltered (Supplementary Fig. 6f). Functional assessment further demonstrated that HID promoted CD8^+^ TIL exhaustion, as evidenced by impaired secretion of IFN-γ, TNF-α, and IL-2 (Fig. 5g), with no such effects observed in TDLNs (Supplementary Fig. 6g). Collectively, these results indicate that dietary iron supplementation exacerbates CD8^+^ T cell ferroptosis and exhaustion within the TME, thereby promoting tumor immune evasion.

Next, we evaluated whether pharmacologically blocking iron accumulation in the TME confers therapeutic benefits. Deferiprone (DFP) is an FDA-approved iron chelator used to treat iron overload in patients with β-thalassemia^65,66^. In the B16-F10 melanoma model, daily DFP was delivered on day 2 after tumor inoculation (Fig. 5h). DFP was well-tolerated, as indicated by no significant body weight loss (Supplementary Fig. 6h). DFP treatment significantly suppressed tumor progression in immunocompetent C57BL/6 mice (Fig. 5i, j). However, DFP treatment did not affect tumor progression in immunodeficient nude mice (Supplementary Fig. 6i-k). Iron quantification in TIF confirmed that DFP effectively reduced iron levels in the TME (Fig. 5k). Correspondingly, flow cytometric analysis demonstrated a marked increase in CD8^+^ T cell infiltration into tumors (Fig. 5l). Further investigation revealed that DFP treatment substantially reduced intracellular iron levels in CD8^+^ TILs (Fig. 5m) and promoted their survival (Fig. 5n) while showing no significant effect on CD8^+^ T cells in TDLNs (Supplementary Fig. 6l, m). Furthermore, DFP treatment specifically enhanced the effector function of CD8^+^ TILs, as evidenced by increased production of IFN-γ and TNF-α (Fig. 5o), but did not affect CD8^+^ T cells in TDLNs (Supplementary Fig. 6n).

These findings were further validated in the ID8 ovarian cancer model (Fig. 5p). DFP treatment potently suppressed tumor progression without affecting body weight (Fig. 5q, r and Supplementary Fig. 6o). Iron levels in malignant ascites were significantly reduced by DFP therapy (Fig. 5s). Further analysis demonstrated that DFP treatment decreased intracellular iron and suppressed ferroptosis specifically in CD8^+^ TILs (Fig. 5t, u), without affecting TDLN CD8^+^ T cells (Supplementary Fig. 6p, q). Functionally, DFP treatment alleviated exhaustion and enhanced effector cytokines production in CD8^+^ TILs (Fig. 5v–x), with no significant impact on their TDLN counterparts (Supplementary Fig. 6r).

In summary, these findings show how iron orchestration critically reprograms CD8^+^ T cell anti-tumor immunity. Specifically, iron supplementation promotes iron overload within the TME, leading to exacerbated ferroptosis of CD8^+^ T cells and consequent tumor immune evasion. Conversely, iron chelation restores CD8^+^ T cell function and reestablishes effective tumor immuno-surveillance.

### *Slc40a1*^OE^-CAR-T cells resist the iron-overloaded TME to potentiate anti-tumor immunity

**Fig. 6:**
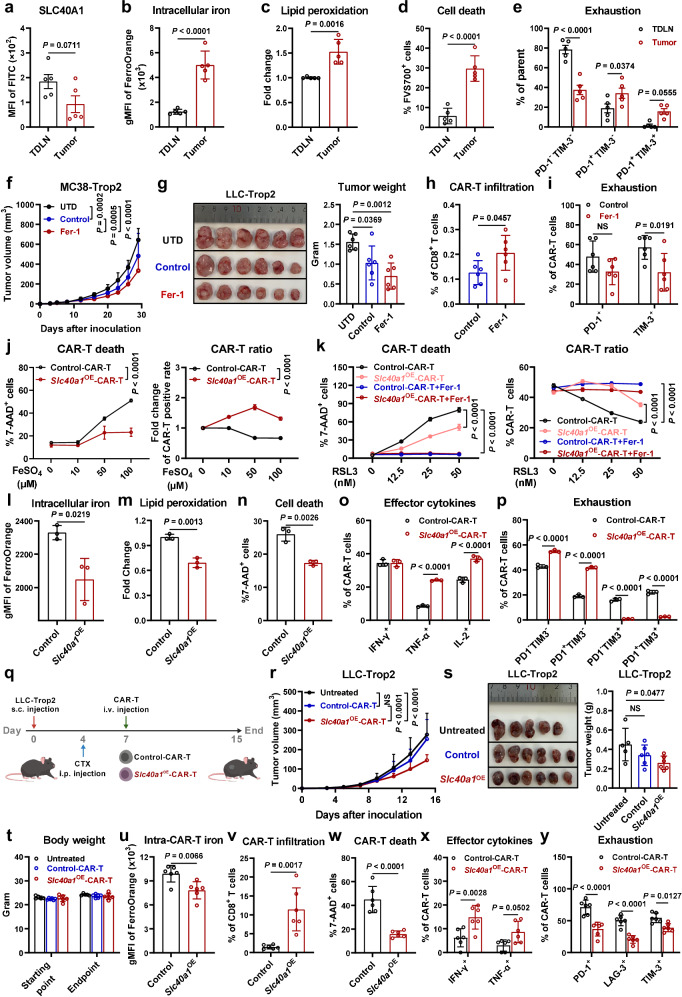
*Slc40a1*^OE^-CAR-T cells resist the iron-overloaded TME to potentiate anti-tumor immunity. **a**–**e** SLC40A1 expression, intracellular iron, lipid peroxidation, cell death, and exhaustion markers expression of CAR-T cells isolated from TDLNs and tumors of mice bearing B16-F10 tumors (*n* = 5 mice). **f**–**i** Fer-1 pretreatment enhances CAR-T cell efficacy in vivo (*n* = 6 mice). Mice were subcutaneously inoculated with 1 × 10^6^ MC38-Trop2 cells on day 0, received cyclophosphamide (CTX, 80 mg/kg, i.p.) on day 4 for lymphodepletion, and injected with 2 × 10^6^ CAR-T cells pretreated with Fer-1 or DMSO for two days via tail vein injection on day 7. **f,** **g** Tumor growth curves, images and weights. **h,** **i** Infiltration and exhaustion markers expression of CAR-T cells. **j,** **k** Cell death and the relative viable proportion of control and *Slc40a1*^OE^ CAR-T cells treated with FeSO_4_ and RSL-3 ± Fer-1 (1 μM) for 24 h (*n* = 3 biologically independent samples). **l**–**p** Intracellular iron, lipid peroxidation, cell death, cytokine secretion, and exhaustion markers expression of control and *Slc40a1*^OE^ CAR-T cells co-cultured with LLC-Trop2 target cells (E: T ratio = 1:2) (*n* = 3 biologically independent samples). **q**–**y** *Slc40a1*^OE^ CAR-T cells exhibit superior antitumor efficacy in an LLC-Trop2 syngeneic model (Mice: Untreated, n = 5; Control, *n* = 6; *Slc40a1*^OE^, *n* = 6). **q** Schematic created with BioGDP.com. Mice were subcutaneously inoculated with 1 × 10^6^ LLC-Trop2 cells on day 0, received cyclophosphamide (CTX, 80 mg/kg, i.p.) on day 4, and were treated with 1 × 10^6^ CAR-T cells via tail vein injection on day 7. **r,** **s** Tumor growth curves, images and weights. **t** Mouse body weight. **u**–**y** Intracellular iron, infiltration, cell death, effector function, and exhaustion markers expression of tumor-infiltrating CAR-T cells. Data are mean ± SD except **e** (mean ± SEM). *P* values were calculated using unpaired two-tailed t test (**a**–**d,** **h,** **l**–**n,** **u**–**w**); two-way ANOVA (**e,** **f,** **i**–**k,** **o,** **p,** **r,** **x,** **y**); one-way ANOVA (**g, s**). Source data are provided as a Source Data file.

CAR-T cell therapy has achieved remarkable success in hematologic malignancies but remains challenged in solid tumors due to the immunosuppressive TME. To investigate whether the iron-overloaded TME in solid tumors similarly induces ferroptosis in CAR-T cells and suppresses their efficacy, we engineered human Trop2-targeted CAR-T cells^67,68^. In vitro, iron induced a dose-dependent CAR-T cell death (Supplementary Fig. 7a). Correspondingly, escalating iron concentrations reduced the proportion of viable CAR-T cells and significantly suppressed their tumor-lytic capacity (Supplementary Fig. 7b, c). More importantly, comparative analysis of peripheral versus tumor-infiltrating CAR-T cells isolated 7 days post-adoptive transfer revealed that intratumoral CAR-T populations exhibited decreased SLC40A1 (Fig. 6a) and ferroptotic phenotypes, including intracellular iron overload, lipid peroxidation, cell death and upregulated exhaustion markers (Fig. 6b–e). To determine whether inhibiting ferroptosis could enhance CAR-T cell efficacy in vivo, we pretreated CAR-T cells with the ferroptosis inhibitor Fer-1 prior to adoptive transfer into MC38 tumor-bearing mice. Fer-1-treated CAR-T cells significantly suppressed tumor growth compared to untreated CAR-T cells (Fig. 6f, g). Analysis of intratumoral CAR-T cells revealed that Fer-1 pretreatment markedly enhanced their infiltration (Fig. 6h). Furthermore, Fer-1-treated CAR-T cells exhibited downregulated expression of exhaustion markers, including PD-1 and TIM-3 (Fig. 6i). Collectively, these findings indicate that the iron-overloaded TME drives CAR-T cell ferroptosis, thereby compromising anti-tumor efficacy.

Thus, to overcome iron dyshomeostasis caused by the iron-overloaded TME and restore CAR-T cell anti-tumor immunity, we engineered CAR-T cells overexpressing *Slc40a1* (*Slc40a1*^OE^-CAR-T) (Supplementary Fig. 7d, e). Surprisingly, *Slc40a1* overexpression minimally impacted T cell viability but enhanced effector cytokine secretion (Supplementary Fig. 7f, g). Critically, compared to control CAR-T cells, *Slc40a1*^OE^-CAR-T cells significantly resisted the iron-overload conditions, as evidenced by preserved viability and enhanced CAR-T ratios (Fig. 6j and Supplementary Fig. 7h–k). In addition, using different ferroptosis inducers, including RSL-3, ML210 and Erastin, confirmed the significant ferroptosis resistance in *Slc40a1*^OE^-CAR-T cells (Fig. 6k and Supplementary Fig. 7l–o).

Subsequently, we employed an in vitro killing assay to validate the efficacy of *Slc40a1*^OE^-CAR-T cells. Compared to control CAR-T cells, the results demonstrated that *Slc40a1*^OE^-CAR-T cells exhibited lower intracellular iron levels during persistent tumor killing (Fig. 6l). Concurrently, *Slc40a1*^OE^-CAR-T cells showed attenuated lipid peroxidation and reduced cell death (Fig. 6m, n). Accordingly, their capacity to secrete effector molecules, including IFN-γ, TNF-α, and IL-2, was significantly enhanced (Fig. 6o). Conversely, markers of tumor-induced CAR-T cell exhaustion were also markedly downregulated (Fig. 6p). Therefore, *Slc40a1* overexpression confers resistance against iron overload-induced ferroptosis, enabling CAR-T cells to sustain tumor-killing functions.

Furthermore, we validated the in vivo anti-tumor efficacy of *Slc40a1*^OE^-CAR-T cells using an LLC tumor-bearing mouse model (Fig. 6q). Compared to control CAR-T cells, *Slc40a1*^OE^-CAR-T cells significantly suppressed tumor growth (Fig. 6r, s) without significantly affecting body weight (Fig. 6t). Flow cytometric analysis revealed that *Slc40a1* overexpression markedly attenuated the iron overload phenotype in CAR-T cells (Fig. 6u). Notably, *Slc40a1*^OE^-CAR-T cells exhibited enhanced tumor infiltration capacity and sustained viability within the TME (Fig. 6v, w), concomitant with increased secretion of effector molecules and significantly reduced expression of exhaustion markers (Fig. 6x, y). These findings were further validated in the B16-F10 melanoma and MC38 colorectal carcinoma models (Supplementary Fig. 7p–y). Thus, *Slc40a1*^OE^-CAR-T cells effectively counteracted the iron-overloaded TME in vivo, resulting in enhanced tumor infiltration, improved cell survival, maintenance of effector functionality, and ultimately, significant suppression of tumor growth.

Collectively, these results demonstrate that by restoring iron homeostasis, *Slc40a1*^OE^-CAR-T cells acquire resistance to ferroptosis within the iron-overloaded TME, thus potentiating a robust anti-tumor immune response.

## Discussion

Iron homeostasis is critical for immune system function, but the iron requirement of CD8^+^ T cells remains debated. Critically, comparisons of iron levels across the primary environments inhabited by CD8^+^ T cells remain unexplored. Prior studies suggested lower free iron in lymph versus blood^69^. In this study, we quantified total iron levels in the main residential environment of CD8^+^ T cells by using ICP-MS. The results showed that although iron deficiency was a common systemic complication in cancer patients, the TME is iron-overloaded. Recent studies have shown significant CD8^+^ T cell proliferation inhibition under iron deficiency but with minimal functional impact^70^, suggesting relatively modest iron requirements for effector functions. Consistently, lymph showed much lower iron than peripheral blood. Using diverse iron preparations, we found that physiological iron levels were sufficient for CD8^+^ T cell proliferation and function. However, pathophysiological iron levels promoted significant CD8^+^ T cell death and dysfunction.

Systemic iron deficiency is a common complication in cancer patients, prompting iron supplementation as an adjuvant therapy in prior research and clinical trials^71–73^. Nevertheless, the impact of iron therapy on tumor progression remains controversial. Compared to immune cells, tumor cells often upregulate iron acquisition to support proliferation. Indeed, we observed higher iron tolerance in tumor cells than in CD8^+^ T cells. Thus, iron supplementation may negatively regulate CD8^+^ T cell anti-tumor immunity. Corroborating this, we found that a high-iron diet impaired CD8^+^ T function and accelerated tumor progression. Conversely, in vivo iron chelation restored CD8^+^ T cell anti-tumor activity, consistent with prior reports supporting iron chelators as therapeutic agents for cancer treatment^74–78^. Notably, previous studies have also shown that iron supplementation can boost type 1 T cell anti-tumor responses and enhance anti-PD-1 therapy^79^. This discrepancy is likely due to differences in study design, such as iron types, doses, timing, and models. Our data show that CD8^+^ T cells require iron within an optimal range: moderate iron supports their function, whereas iron overload in the tumor microenvironment induces T cell dysfunction. Thus, iron exerts dual effects on anti-tumor immunity depending on its concentration. These findings underscore the necessity for precise iron regulation in cancer treatment.

Ferroptosis is an iron-dependent cell death driven by lipid peroxidation^36–38^, which also regulates T cell homeostasis^28,80,81^. Prior studies demonstrate that CD8^+^ T cells induce tumor ferroptosis via IFN-γ-mediated SLC7A11 downregulation and ACSL4-dependent lipid remodeling^82,83^. However, whether tumors reciprocally orchestrate ferroptosis in CD8^+^ T cells remains unresolved. Using in vitro and in vivo models, we showed that the iron-overloaded TME triggered CD8^+^ T cell ferroptosis. It is noteworthy that, despite residing in the same iron-overloaded TME, CD8^+^ T cells exhibit greater sensitivity to ferroptosis than tumor cells. This heightened vulnerability stems from their intrinsically lower expression of ferroptosis-protective mechanisms and distinct metabolic architecture. In general, activated CD8^+^ T cells coordinately upregulated iron import (TfR1), storage (Ferritin), and export (SLC40A1) proteins to maintain iron homeostasis. However, chronic TCR stimulation within tumors disrupted this balance by suppressing SLC40A1 transcription and translation, leading to a profound decrease in SLC40A1 expression. Additionally, in vivo knockout and overexpression experiments revealed that tumor-derived hepcidin further exacerbated SLC40A1 degradation in CD8^+^ TILs. This dysregulation drove intracellular iron overload in CD8^+^ TILs, culminating in ferroptosis and exhaustion. Whether host-derived hepcidin contributes to this process, as well as the local concentrations of hepcidin within the TME, remains to be determined.

Adoptive T cell therapy (ACT), including TIL, CAR-T, and T cell receptor-engineered T cell (TCR-T) therapies, is a rapidly evolving pillar of cancer immunotherapy^84,85^. Despite success in hematologic malignancies, ACT efficacy in solid tumors remains limited, largely due to the suppressive TME^86,87^. During our investigation into the application of CAR-T therapy for solid tumors, we consistently observed extensive ferroptosis of CAR-T cells within the tumor sites, mediated by the iron-overloaded TME. To promote CAR-T fitness, we engineered *Slc40a1*-overexpression CAR-T cells to reprogram their iron homeostasis. Notably, *Slc40a1* overexpression minimally impacted T cell viability but enhanced effector cytokine secretion. Critically, *Slc40a1*^OE^-CAR-T cells resisted the iron-overloaded TME, maintained superior viability and tumor infiltration, and exhibited potent anti-tumor immunity. Therefore, *Slc40a1* overexpression may represent an effective strategy for ACT, enhancing its resistance to the suppressive TME.

In summary, we revealed a mechanism of immune evasion involving tumor necrosis-induced iron-overloaded TME and subsequent CD8^+^ T cell ferroptosis. Within CD8^+^ TILs, persistent TCR signaling disrupts iron homeostasis, causing multi-level downregulation of the iron exporter SLC40A1 through transcriptional suppression, translation impairment, and hepcidin-mediated degradation. SLC40A1 repression resulted in CD8^+^ T cell iron overload and consequent ferroptosis. Therefore, *Slc40a1* overexpression reprograms iron homeostasis in adoptively transferred T cells, conferring resistance to the iron-overloaded TME and robust anti-tumor efficacy (Supplementary Fig. 8).

## Methods

All experiments complied with relevant ethical guidelines and were approved by the institutions and ethics committees listed in the pertinent sections of the manuscript.

### Cell lines

B16-F10, B16-F10-Trop-2, LLC, LLC-Trop-2, MC38, MC38-Trop2, 4T1, ID8-Luc, HEK-293T, HepG2 and their derived cell lines were cultured in DMEM (Gibco, C11995500BT) with 10% Fetal bovine serum (FBS, Sigma-Aldrich, F8318) and 1% Penicillin-Streptomycin (Pen-Strep, Gibco, 15140-122) at 37 °C and 5% CO_2_. NCI-H446, NCI-H1299, NCI-H1703 and PC-9 cells were cultured in RPMI 1640 (Gibco, C11875500BT) with 10% FBS and 1% Pen-Strep at 37 °C and 5% CO_2_. All commercially sourced cell lines were authenticated by the vendors or published sources. All cell lines used in this study were routinely screened for mycoplasma contamination by PCR, and tumor cell lines were implanted during exponential growth.

### Animals

Seven-week-old wild-type C57BL/6J mice (both male and female) and male BALB/c-nude mice were purchased from GemPharmatech Co., Ltd (Jiangsu, China). Female C57BL/6J mice were used for the ID8 ovarian cancer model, while male C57BL/6J mice and male BALB/c-nude mice were used for other tumor models. Male Sprague-Dawley (SD) rats (280-300 g) were obtained from Slike Jingda Laboratory Animal Co., Ltd (Hunan, China). All animals were maintained under specific pathogen-free (SPF) conditions in a controlled environment (22 ± 2 °C, 50 ± 10% humidity, 12/12 h light/dark cycle) with free access to food and water. All mouse experiments were approved by the Institutional Animal Care and Use Committee (IACUC) of Guangzhou National Laboratory, GZLAB-AUCP-2024-04-A02.

### Human sample assessment

All procedures involving peripheral blood from healthy donors and patient samples were approved by the Institutional Review Boards of the Third Affiliated Hospital of Sun Yat-sen University. Written informed consent was obtained from each subject prior to sample collection. Human cancer tissues and paired peripheral blood were collected for flow cytometry, tumor interstitial fluid and plasma isolation. Detailed clinical information of the participants is provided in Supplementary Table 1. The Medical Ethics Committee of the Third Affiliated Hospital of Sun Yat-sen University approved this study. All human tissue samples were consumed during the experiments and disposed of according to institutional biosafety regulations. Peripheral blood from healthy donors for peripheral blood mononuclear cell (PBMC) and T cell isolation was purchased from Hycells (CAT# hPBLP001, Shanghai, China).

### T cells isolation, activation and culture

Mouse CD8^+^ T cells were isolated from spleen and lymph nodes and separated using the EasySep Mouse CD8^+^ T Cell Isolation Kit (STEMCELL, 19853). For activation, 24-well plates were pre-coated overnight at 4 °C with 0.02 mg/mL rabbit anti-hamster IgG (Invitrogen, A18897) in PBS. Cells were activated in the coated plates using RPMI 1640 (supplemented with 10% FBS) or iron-deficient serum-free MT101 medium (TransGen, MT101). The activation medium contained 50 μM 2-mercaptoethanol (2-ME, Sigma-Aldrich, M3148), 1% Pen-Strep, 0.25 μg/mL anti-CD3 (eBioscience, 16-0031-85), 1 μg/mL anti-CD28 (eBioscience, 16-0281-85), and 10 ng/mL IL-2 (Peprotech, 212-12-1MG). For iron-treatment experiments, 2-ME was omitted from the medium. All cultures were maintained at 37 °C with 5% CO_2_. Cells were harvested at 24 or 48 h for downstream analysis; following activation, cells were maintained in medium containing 10 ng/mL IL-2 and 50 μM 2-ME.

Human CD8^+^ T cells were isolated from PBMCs obtained from healthy donors. PBMCs were first isolated by Lymphoprep density gradient centrifugation (STEMCELL, 07801), after which CD8^+^ T cells were purified using the EasySep™ Human CD8^+^ T Cell Isolation Kit (STEMCELL, 17953). Cells were activated for 48 h in 24-well plates pre-coated with 2 μg/mL anti-human CD3 (eBioscience, 16-0037-85) and 1 μg/mL anti-human CD28 (eBioscience, 16-0289-85). Cells were cultured in either RPMI 1640 (supplemented with 10% FBS) or MT101 supplemented with 10 ng/mL human IL-2 (Peprotech, 200-02-1MG) at 37 °C with 5% CO_2_, and subsequently used for functional assays.

### Chronic TCR stimulation model

To induce dysfunction, activated mouse CD8^+^ T cells were restimulated every 48 h for three cycles using plates re-coated with 0.5 μg/mL anti-CD3 and 0.2 μg/mL anti-CD28 in the presence of 10 ng/mL IL-2. Control cells were maintained in IL-2 alone without restimulation. For human CD8^+^ T cells, a parallel protocol was followed using plates coated with 0.5 μg/mL anti-CD3 and 0.5 μg/mL anti-CD28. Dysfunctional phenotypes were assessed at endpoint via surface PD-1/TIM-3 staining and intracellular IL-2/TNF-α staining after PMA (STEMCELL, 74042), ionomycin (STEMCELL, 73722), Brefeldin A (eBioscience, 00-4506-51), and Monensin (eBioscience, 00-4505-51) stimulation.

### PCR and cloning

Murine *Slc40a1* mRNA (NM_016917.2) was amplified from cDNA synthesized with RevertAid First Strand cDNA Synthesis Kit (Thermo Fisher Scientific, K1621) from total RNA purified from mouse liver. The following primers were used: forward primer 5’ CTCCAACCCGCTCCCATAA; and reverse primer 5’ CAGGGGCCACAGCTAAACTAT. The *Slc40a1* cDNA was then cloned into MIGR1 and MIGR1-antiTROP2-m28z retroviral vectors^67,68^. The full-length murine *Hamp1* (NM_032541.1) cDNA was synthesized by Tsingke Biotechnology (Beijing, China) and directionally cloned into the pHAGE-MCS-3 × HA (NovoPro, V013646) lentiviral vector using BamHI and XhoI restriction sites. The construct was sequence-verified by Sanger sequencing prior to functional validation.

### Generation of *Hamp1*-overexpressing and *Hamp1*-knockout cell lines

Lentiviruses were produced by co-transfecting HEK-293T cells with pHAGE-MCS-*Hamp1*, psPAX2 (Addgene, 12259), and pMD2.G (Addgene, 12260) using ExFect Transfection Reagent (Vazyme, T101-01) at a 2:1 reagent-to-DNA ratio. The viral supernatant was harvested at 48 h post-transfection and used to transduce B16-F10 and LLC cells. Successfully transduced cells were selected with puromycin (YEASEN, 727136ES03) at 4 μg/mL for LLC cells and 2 μg/mL for B16-F10 cells for 72 h. *Hamp1* overexpression was confirmed by qPCR and immunoblotting.

For *Hamp1* knockout, the PX458 plasmid (Addgene, 48138) was linearized by restriction digestion with BbsI. Guide RNA oligonucleotides were annealed and inserted into the linearized vector using homologous recombination. The resulting constructs were verified by sequencing. B16-F10 and LLC cells were then transiently transfected with the validated PX458 plasmids encoding *Hamp1*-targeting guide RNAs. At 48 h post-transfection, GFP-positive single cells were sorted by flow cytometry into 96-well plates to establish monoclonal knockout cell lines. Guide RNA sequences are presented in Supplementary Table 2.

### *Slc40a1* overexpression CD8^+^ T cell and CAR-T cell generation

Retrovirus production and mouse CD8^+^ T cell transduction was conducted as described previously^67,68^. Briefly, HEK-293T cells were co-transfected with 12 μg of the respective retroviral plasmid (MIGR1, MIGR1-m-*Slc40a1*^OE^, MIGR1-antiTROP2-m28z, MIGR1-antiTROP2-m28z-m-*Slc40a1*^OE^) and 6 μg pCL-Eco packaging plasmid (Addgene, 12371) using ExFect Transfection Reagent at a 2:1 reagent-to-DNA ratio. Viral supernatants were harvested at 72 and 96 h post-transfection, filtered through a 0.45-μm filter (Jet Biofil, FPE404025), and stored at -80 °C. Mouse CD8^+^ T cells were isolated and activated for 2 days, and then the activated cells were transduced on RetroNectin (Takara, T100B)-coated plates via spinfection (800 × *g*, 32 °C, 90 min) with viral supernatant supplemented with 8 μg/mL polybrene (YEASEN, 40804ES76) and 5 ng/mL IL-2. Transduction efficiency was quantified by flow cytometry based on GFP expression.

### In vitro functional assays

Proliferation of CD8^+^ T cells was quantified by CFSE dilution. Briefly, isolated cells were labeled with 1 μM CFSE (Invitrogen, C34554) in PBS for 10 min at 37 °C. The reaction was quenched with a 5-fold volume of complete medium, followed by two washes with PBS. Labeled cells were then co-cultured with stimulators for 48 h, and proliferation was analyzed by flow cytometry based on CFSE dilution.

Tumor cell proliferation was determined using the CCK-8 assay. Tumor cells were seeded at 1 × 10³ cells per well in a 96-well plate and were measured every 12-24 h for 5 days by adding 10 μL of CCK-8 reagent (GLPBIO, GK10001) and incubating for 1.5 h, after which the absorbance at 450 nm was recorded.

Cytotoxicity was evaluated by measuring lactate dehydrogenase (LDH) release. Effector T cells and target tumor cells were co-cultured at specified effector-to-target ratios (E: T ratio) for 24 h. The LDH activity in the supernatant was quantified using the CytoTox 96® Non-Radioactive Cytotoxicity Assay (Promega, G1780). Specific cytotoxicity was calculated as follows: Specific Lysis (%) = [(Experimental LDH Release - Spontaneous Release) / (Maximum Release - Spontaneous Release)] × 100.

### Polysome profiling analysis

T cells (4 × 10⁷ cells/sample) were treated with 100 μg/mL cycloheximide (CHX, MedChemExpress, HY-12320) in complete RPMI-1640 medium at 37 °C for 15 min, followed by two washes with PBS containing 100 μg/mL CHX. Cells were lysed in 300 μL ice-cold lysis buffer (25 mM Tris-HCl pH 7.4, 100 mM NaCl, 5 mM MgCl_2_, 1% Triton X-100, 1% sodium deoxycholate, 40 U/mL RNase inhibitor, 1 mM DTT, 1 mM PMSF, and 100 μg/mL CHX) for 30 min on ice. Lysates were cleared by centrifugation at 13,000 × *g* for 10 min at 4 °C. Linear 10-45% (w/v) sucrose density gradients were prepared in gradient buffer (25 mM Tris-HCl pH 7.4, 100 mM NaCl, 5 mM MgCl_2_, 40 U/mL RNase inhibitor, 1 mM PMSF, 100 μg/mL CHX) using a Gradient Master (BioComp Instruments). Cleared lysates were layered atop gradients and centrifuged at 288,000 × *g* for 3 h at 4 °C in a Beckman Optima XPN-100 ultracentrifuge with SW41 Ti rotor. Gradients were fractionated using a BioComp Piston Gradient Fractionator with continuous monitoring at 260 nm absorbance. A total of 15 fractions were collected per sample. The fractions corresponding to 40S, 60S, 80S ribosomal subunits, and polysomes were identified based on the characteristic UV absorbance profile. Total RNA from the indicated fractions was isolated using VeZol Reagent (Vazyme, R411) for subsequent qRT-PCR analysis.

### Transmission electron microscopy sample preparation and imaging

Mouse CD8^+^ T cells were fixed in 2.5% Glutaraldehyde Fixative (Biosharp, BL911A) at 4 °C overnight, followed by post-fixation with 1% osmium tetroxide for 1 h. Samples were dehydrated through an ethanol series (30% to 100%) and embedded in EPON 812 resin (Sigma-Aldrich). Ultrathin sections (70 nm) were cut using a Leica EM UC7 ultramicrotome with a diamond knife (Diatome) and collected on 50-mesh copper grids. Grids were stained with uranyl acetate (2% in methanol) and lead citrate (0.4%) prior to imaging. Mitochondria were visualized using a Thermo Fisher Talos L120C TEM operated at 120 kV, with images captured at 11000 × magnification using a Ceta 16 M CCD camera. Mitochondrial long-axis lengths were measured using ImageJ (NIH) with scale calibration from TEM magnification standards.

### In vivo tumor implantation and therapeutic intervention

For tumor implantation, 2 × 10^5^ B16-F10, 2 × 10^5^ B16-F10-Trop2, 5 × 10^5^ LLC or 10^6^ LLC-Trop2 tumor cells were injected subcutaneously in 100 μL PBS. For orthotopic ID8-Luc models, 1.5 × 10⁶ cells in 100 μL PBS were administered intraperitoneally. Tumors were measured via caliper every 2–3 days post tumor engraftment with or without the indicated treatments, and tumor volume was calculated by volume = (length × width × height) / 2. The maximal tumor size permitted by the ethics committee was 2000 mm^3^. In some cases, this limit was exceeded on the final measurement day, and those mice were immediately euthanized.

For the high-iron diet (HID) experiment, mice in the HID group received ferrous sulfate (Aladdin, F116338) via oral gavage at 300 mg/kg in 300 μL ultrapure water every other day, commencing 4 weeks prior to tumor inoculation and maintained throughout the study. The standard iron diet (SID) group was administered equal volumes of ultrapure water on the identical schedule. All treatments continued until the experimental endpoint.

For deferiprone (DFP) intervention, tumor-bearing mice received daily intraperitoneal injections of DFP (150 mg/kg; Sigma-Aldrich, #379409) in sterile 0.9% saline (0.22-μm filtered) or vehicle control. In the B16-F10 model, treatment was initiated on day 2 post-inoculation and continued for two weeks, followed by a one-week drug withdrawal before endpoint analysis. In the ID8-Luc model, treatment was initiated on day 7 post-inoculation and continued for five weeks, followed by an 18-day withdrawal period. ID8-Luc tumor progression was monitored weekly via bioluminescence imaging using an AniView100 Pro multimodal in vivo imaging system (BLT, China) after 150 mg/kg D-luciferin (YEASEN, 40701ES01) was intraperitoneally injected.

For CAR-T cell therapy experiments, mice bearing subcutaneous B16-F10-Trop2, LLC-Trop2 or MC38-Trop2 tumors underwent lymphodepletion via intraperitoneal injection of cyclophosphamide (CTX, 80 mg/kg in 100 μL sterile PBS) on day 4 post-inoculation. CAR-T cells (1 × 10⁶ cells per mouse) were administered by tail vein injection on day 7.

### Tissue dissociation and single-cell isolation

Spleen and lymph nodes were mechanically dissociated through 100-μm cell strainers (Falcon, 352360) in MACS buffer (PBS + 2 mM EDTA + 1% FBS). Erythrocytes were lysed using RBC Lysis Buffer (Yuanye Bio-Technology, R20176) for 2 min at room temperature (RT), and the reaction was stopped by adding a 10 × volume of ice-cold MACS buffer.

For tumor tissues, specimens were minced into fragments smaller than 1 mm^3^ in RPMI-1640 complete medium supplemented with 0.1 mg/mL DNase I (HARVEYBIO, EZ1179), 1 mg/mL Collagenase Type II (Sigma-Aldrich, V900892), 1 mg/mL Collagenase Type IV (Sigma-Aldrich, V900893), and 2.5 μg/mL Hyaluronidase (Sigma-Aldrich, H3506). The mixtures were then digested for 30 min at 37 °C with gentle agitation (200 rpm). After digestion, the slurry was filtered through 70-μm cell strainers (Falcon, 352350) and further dissociated using a syringe plunger. Finally, cells were washed with MACS buffer and pelleted by centrifugation at 500 × *g* for 5 min to obtain single-cell suspensions.

### Flow cytometry analysis

For surface marker staining, single-cell suspensions were incubated with fluorochrome-conjugated antibodies in MACS buffer for 15 min at RT in the dark. For intracellular cytokine detection, cells were first restimulated ex vivo in RPMI 1640 containing 10% FBS, PMA, and ionomycin in the presence of protein transport inhibitors (brefeldin A and monensin) for 4-6 h at 37 °C under 5% CO_2_. Following stimulation, cells were stained for surface markers as described. For intracellular targets, two fixation and permeabilization procedures were used. For intracellular cytokine staining, cells were fixed overnight at 4 °C using IC Fixation Buffer (eBioscience, 00-8222-49) and then permeabilized with 1 × Permeabilization Buffer (eBioscience, 00-8333-56). For transcription factor staining, cells were fixed overnight at 4 °C with Foxp3/Transcription Factor Staining Buffer (eBioscience, 00-5523-00) and subsequently washed with the corresponding 1× permeabilization buffer. After permeabilization, cells were incubated with intracellular antibodies for 30 min at RT in the dark. All samples were analyzed on a flow cytometer, and the following antibodies were used in this study: FVD506 (1:1000, 65-0866-14), FITC anti-mouse TCR β (1:100, 11-5961-82), eF450 anti-mouse CD90.2 (1:100, 48-0902-82), eF506 anti-mouse CD4 (1:100, 69-0042-82), SB600 anti-mouse CD8a (1:100, 63-0081-82), APC anti-mouse PD-1 (1:100, 17-9985-82), eF710 anti-mouse TIM3 (1:100, 46-5870-82), PE-Cy7 anti-mouse LAG-3 (1:100, 25-2231-82), eF450 anti-mouse IFN-γ (1:100, 48-7311-82), APC anti-mouse IFN-γ (1:100, 17-7311-82), PerCP Cy5.5 anti-mouse IL-2 (1:100, 45-7021-82), PE-Cy7 anti-mouse GranzymeB (1:100, 25-8898-82), PE anti-mouse TfR1 (1:100, 12-0711-81), APC anti-mouse CD25 (1:100, 17-0251-82), SB600 anti-mouse CD69 (1:100, 63-0691-82), PE anti-human TfR1 (1:100, 12-0719-42), PerCP eF710 anti-human TIM3 (1:100, 46-3109-42), and APC anti-human CD4 (1:100, 17-0047-42) (all from eBioscience); anti-mouse GPX4 (1:1000, MA5-32827), and AF488 goat anti-rabbit (1:100, SA5-10384-AFP488) (all from Invitrogen); FVS700 (1:1000, 564997), 7-AAD (1:100, 559925), APC-Cy7 anti-mouse CD45 (1:100, 557659), APC anti-mouse CD4 (1:100, 553051), PE anti-mouse TNF (1:100, 554419), BV650 anti-Ki-67 (1:100, 563757), BV421 anti-mouse CD25 (1:100, 562606), PE-Cy7 anti-mouse CD11b (1:100, 552850), PE anti-mouse F4/80 (1:100, 565410), APC anti-mouse NK-1.1 (1:100, 550627), BV605 anti-mouse CD11c (1:100, 563057), V500 anti-mouse I-A/I-E (1:100, 562366), BV421 anti-human PD-1 (1:100, 564323), APC Cy7 anti-human CD3 (1:100, 557832), and BV510 anti-human CD8 (1:100, 743065) (all from BD Biosciences); FITC anti human/mouse SLC40A1 (1:100, Novus, NBP2-75923F). A representative gating strategy is shown in Supplementary Fig. 9.

### Fluorescent probes staining

For detection of the intracellular iron, cells were washed twice with Dulbecco’s Phosphate-Buffered Saline (DPBS) and subsequently incubated with 1 μM FerroOrange (Dojindo, F374) or RhoNox-1 (MedChemExpress, HY-D1533) in serum-free medium MT101 for 30 min at 37 °C in the dark. Stained cells were analyzed by flow cytometry, and the intracellular iron levels were quantified using the geometric mean fluorescence intensity (gMFI) of the PE channel.

To assess lipid peroxidation, cells were washed twice with Hank’s Balanced Salt Solution (HBSS) and then incubated with 1 × BDP 581/591 C11 (Dojindo, L267) or BODIPY™ 665/676 (Invitrogen, B3932) working solution for 30 min at 37 °C in the dark. After two additional washes with HBSS, cells were immediately analyzed by flow cytometry.

For total cellular ROS measurement, cells were washed twice with PBS and loaded with 1 μM CM-H2DCFDA (Invitrogen, C6827) in PBS for 30 min at 37 °C in the dark. Following two washes with PBS, cells were further incubated in complete growth medium for 30 min at 37 °C to allow for complete intracellular de-esterification. After two final washes with PBS, oxidant-sensitive fluorescence was quantified by flow cytometry using FITC channel.

### TIF, serum, lymph fluid, plasma, ascites and pleural effusion collection

Tumor interstitial fluid (TIF) was collected from fresh tumor or adjacent normal tissues using a previously described approach^88^. Tumors were briefly rinsed in PBS and blotted on filter paper. The tumors were then put onto 10 μm nylon filter (Membrane Solutions) affixed atop 50 mL conical tubes, and centrifuged at 106 × *g* for 20 min at 4 °C. The resulting TIF was collected from the tube bottom.

For serum isolation, mouse blood was obtained via retro-orbital bleeding. The blood was allowed to clot at RT, and serum was subsequently isolated by centrifugation at 500 × *g* for 10 min.

Lymph fluid was collected from C57BL/6 mice or SD rats using a previously described approach^89^. Animals were anesthetized by intraperitoneal injection of 1.25% tribromoethanol (Sigma-Aldrich, T48402), followed by aseptic laparotomy for direct sampling from the superior mesenteric lymph duct. Following the procedure, animals were euthanized. Lymph samples were centrifuged at 500 × *g* for 10 min, and the supernatants were collected.

For murine ascites collection, mice were euthanized and the abdominal skin was aseptically incised to expose the intact peritoneal muscle layer. Ascitic fluid was then carefully aspirated using a sterile syringe.

Plasma was obtained from peripheral blood drawn into EDTA-coated tubes and centrifuged at 500 × *g* for 10 min. Similarly, malignant pleural effusion samples from lung cancer patients were aseptically aspirated and centrifuged under the same conditions to obtain cell-free supernatants.

All samples were snap-frozen in liquid nitrogen and stored at -80 °C until further analysis.

### Trace element quantification by ICP-MS

For iron quantification, serum, TIF and lymph fluid samples were collected and processed under trace metal-clean conditions to prevent exogenous iron contamination. Sample aliquots were diluted with inverse aqua regia (3:1 HNO_3_/HCl) and subjected to complete digestion using a Milestone UltraCLAVE IV microwave digestion system (temperature program: ramp to 240 °C, hold for 40 min, followed by overnight cooling within sealed vessels). Iron concentrations were quantified by inductively coupled plasma mass spectrometry (ICP-MS) on an iCAP Qc instrument (Thermo Fisher Scientific) operated in standard mode (monitored isotope: ^57^Fe). Quantification employed matrix-matched external calibration standards with internal standardization for drift correction. Blank samples (acid mixture without biological matrix) were processed identically in each digestion batch, and all reported Fe concentrations were corrected by subtracting mean blank values.

For multi-element quantification, serum and lymph fluid samples were analyzed by Beijing BioTNT Biotechnologies Ltd. In brief, aliquots were digested with electronic-grade HNO_3_: H_2_O_2_ (2:1) at 150 °C for 2 h using microwave-assisted digestion, followed by evaporation to near-dryness. Residues were reconstituted in 2% HNO_3_ to 50 mL final volume. Element quantification was performed via ICP-MS under standardized analytical protocols.

### Measurement of iron content

Relative iron content in biological fluids was determined using a commercial Serum Ferri Ion Content Assay Kit (Solarbio, BC1735) according to the manufacturer’s instructions. For limited-volume samples, uniform dilution with ultrapure water was applied to maintain a consistent reaction volume. Absorbance at 520 nm was measured using a BioTek Synergy H1 microplate reader (Agilent Technologies), with blank-corrected values used for quantification.

Relative ferrous iron (Fe^2+^) content in biological samples was determined using a commercial Ferrous Iron Content Detection Kit (Yeasen, 40374ES48) according to the manufacturer’s instructions. For limited-volume samples, uniform dilution with ultrapure water was applied to maintain a consistent reaction volume. Absorbance at 593 nm was measured using a BioTek Synergy H1 microplate reader.

For cellular iron quantification, cell lysates were prepared using the Cell Iron Content Assay Kit (Solarbio, BC5310) with automated cryo-homogenization. Cells in extraction buffer were processed in 1.5 mL microtubes using a refrigerated ultrasonicator (Bioruptor® Pico, Diagenode; water bath temperature: 4 °C ± 1 °C) with pulsed sonication (200 W, 30 s ON/30 s OFF, 30 cycles). Homogenates were centrifuged at 8000 × *g* for 10 min at 4 °C. Supernatants were analyzed according to the manufacturer’s protocol, with absorbance at 510 nm measured on BioTek Synergy H1.

### Prussian blue iron staining

Paraffin-embedded tissue sections were dewaxed and rehydrated through a graded ethanol series. Iron staining was performed using a Prussian Blue Iron Stain Kit (Ferric Iron, enhanced with DAB, Solarbio, G1428) according to the manufacturer’s instructions. Briefly, sections were sequentially incubated with Perls’ working solution, signal incubation solution, and DAB enhancement solution, with gentle washing between steps. Nuclear counterstaining, distilled water rinse, ethanol dehydration, xylene clearance, and resin mounting preceded brightfield microscopy imaging. Ferric iron deposits were visualized as distinct brownish-yellow granules, while nuclei appeared blue.

### qRT-PCR and Western blot

Total RNA was isolated using FastPure Cell/Tissue RNA Kit (Vazyme, RC101), reverse-transcribed with HiScript II Q RT SuperMix (Vazyme, R223), and subjected to qPCR with ChamQ Universal SYBR qPCR Master Mix (Vazyme, Q711) on a StepOnePlus Real-Time PCR System (Applied Biosystems). Primer sequences are listed in Supplementary Table 3.

For western blotting, cells were washed and lysed in RIPA buffer (NCM Biotech, WB3100) containing protease inhibitor cocktail (Beyotime, P1005). Lysates were resolved on 10% or 15% SDS-PAGE (PG112, PG114, Epizyme) and transferred to 0.45 μm PVDF membranes (Millipore, IPFL00005) or 0.22 μm membranes (Millipore, ISEQ00010) using the eBlot^TM^ L1 (GenScript, L00686C). After blocking with 5% non-fat dairy milk for 90 min at RT, membranes were incubated with primary antibodies at 4 °C overnight, then HRP-conjugated secondary antibodies. Signals were developed with NcmECL Ultra (NCM Biotech, P10100) on Tanon-5200 (Tanon Science & Technology) using β-actin or GAPDH as loading controls. The following antibodies were used in this study: TfR1 (1:1000, Invitrogen, 13-6800), DMT1 (1:1000, Abcam, ab55735), FTH1 (1:1000, Cell Signaling Technology, 4393), FTL (1:1000, Proteintech, 10727-1-AP), SLC40A1 (1:1000, Novus, NBP1-21502), HAMP1 (1:500, Abcam, ab190775), Caspase-3 (1:1000, Cell Signaling Technology, 9662S), Cleaved Caspase-3 (1:1000, Cell Signaling Technology, 9664 T), ACSL3 (1:1000, Abclonal, A22085), FSP1 (1:1000, Proteintech, 68049), SLC7A11 (1:1000, Cell Signaling Technology, 98051S), GPX4 (1:1000, Cell Signaling Technology, 59735 T), β-actin (1:5000, RayBiotech, RM2001), GAPDH (1:5000, RayBiotech, RM2002), mouse IgG (1:10000, RayBiotech, RM3001), rabbit IgG (1:10000, RayBiotech, RM3002).

### Bioinformatics analysis

Analysis of publicly available 4T1 spatial transcriptomics data was performed using the Giotto toolbox in R for clustering and spatial gene expression analysis.

Gene expression levels in exhausted versus non-exhausted CD8^+^ T cell populations were analyzed across pan-cancer single-cell RNA sequencing datasets using the scRNA-seq Data Portal for T cell in Pan-Cancer (http://cancer-pku.cn:3838/PanC_T/).

Gene set enrichment analysis (GSEA) was applied to a pre-ranked gene list from exhausted *vs*. non-exhausted T cells to evaluate ferroptosis-related pathways enrichment, using default parameters and significance thresholds (FDR < 0.25, nominal *p* < 0.05).

To investigate ferroptosis-related pathway activity associated with *SLC40A1* expression, gene set variation analysis (GSVA) was performed on single-cell RNA-sequencing data stratifying CD8^+^ T cells into *SLC40A1*^Low^ and *SLC40A1*^High^ groups, with differential enrichment assessed using the Wilcoxon rank-sum test and FDR adjustment.

Using data from The Cancer Genome Atlas (TCGA), we compared *HAMP* expression between tumor and adjacent normal tissues across multiple cancer types. Expression values, represented as log_2_(FPKM + 1), were visualized using violin plots. For survival analysis, Kaplan-Meier curves were generated, stratifying patients based on an optimal expression cutoff for *HAMP*.

The accession codes and detailed information for all publicly available datasets utilized in these analyses are provided in the Supplementary Table 4.

### Statistics and reproducibility

All statistical analyses were performed using GraphPad Prism, Microsoft Excel, and R software. Detailed statistical methods, including sample sizes (n) and the exact *P* values, are provided in the figure legends or displayed in the corresponding figures. Data are presented as mean ± SD unless otherwise indicated. Schematic diagrams were created with BioGDP^90^. All experiments were repeated at least three times independently with similar results. Key resources used in this study are listed in Supplementary Table 5.

### Reporting summary

Further information on research design is available in the Nature Portfolio Reporting Summary linked to this article.

## Supplementary information

Supplementary Information

Reporting Summary

Transparent Peer Review file

## Source data

Source Data

## Data Availability

This paper analyzes existing, publicly available data from the original research article and accession numbers for the external datasets are indicated in the Supplementary Table 4. All other data supporting the findings of this study are available within the article, its Supplementary Information and Source Data. Source data are provided with this paper.
