# Supplementary material for: Iron overload in the tumor microenvironment induces CD8+ T cell ferroptosis and dysfunction
Source: Nat Commun. 2026 May 22;17:6754. doi: 10.1038/s41467-026-73379-4 (PMC13385831; doi:10.1038/s41467-026-73379-4)
Supplement: Supplementary file 1 — Supplementary Information [file 41467_2026_73379_MOESM1_ESM.pdf]

Supplementary Information

Iron overload in the tumor microenvironment induces CD8$^+$ T cell ferroptosis and dysfunction

Zhenyu Lin (林桢毓) [1, 2], Huanpeng Chen (陈焕鹏) [1], Yujing Ke (柯雨景) [1], Hanyue Xiao (肖涵月) [1, 3, 4], Chao Li (李超) [1, 3, 4], Zilong Wu (吴紫龙) [1, 5], Huixin Gao (高惠昕) [6], Nanqi Huang (黄南祺) [7], Lijuan Lu (卢丽娟) [8], Peng Sun (孙鹏) [9, 10], and Yingjie Bian (边英杰) [1, 3, 4, *]

[1] Guangzhou National Laboratory, Guangzhou International Bio-Island, Guangzhou, Guangdong 510005, China.

[2] Zhongshan School of Medicine, Sun Yat-sen University, Guangzhou, Guangdong 510080, China.

[3] State Key Laboratory of Respiratory Disease, Guangzhou Institute of Respiratory Health, the First Affiliated Hospital of Guangzhou Medical University, Guangzhou, Guangdong 510120, China.

[4] Guangzhou Medical University, Guangzhou, Guangdong 511436, China.

[5] Department of Gastrointestinal Surgery, Union Hospital, Tongji Medical College, Huazhong University of Science and Technology, Wuhan, Hubei 430022, China.

[6] Department of Clinical Laboratory, Guangzhou Women and Children Medical Center, Guangzhou Medical University, Guangzhou, Guangdong 510600, China.

[7] Department of Gastrointestinal Surgery, Guangdong Provincial Key Laboratory of Major Obstetric Diseases, Guangdong Provincial Clinical Research Center for Obstetrics and Gynecology, the Third Affiliated Hospital of Guangzhou Medical University, Guangzhou, Guangdong 510150, China.

[8] Department of Medical Oncology, the Third Affiliated Hospital of Sun Yat-sen University, Guangzhou, Guangdong 510630, China.

[9] State Key Laboratory of Oncology in South China, Collaborative Innovation Center for Cancer Medicine, Guangzhou, Guangdong 510060, China.

[10] Department of Pathology, Sun Yat-sen University Cancer Center, Guangzhou, Guangdong 510060, China.

[*] Corresponding author: Dr. Yingjie Bian,

Address: Guangzhou National Laboratory, Guangzhou International Bio-Island, Guangzhou, 510005, China,

Tel: 86-13811123714

E-mail: bian_yingjie@gzlab.ac.cn

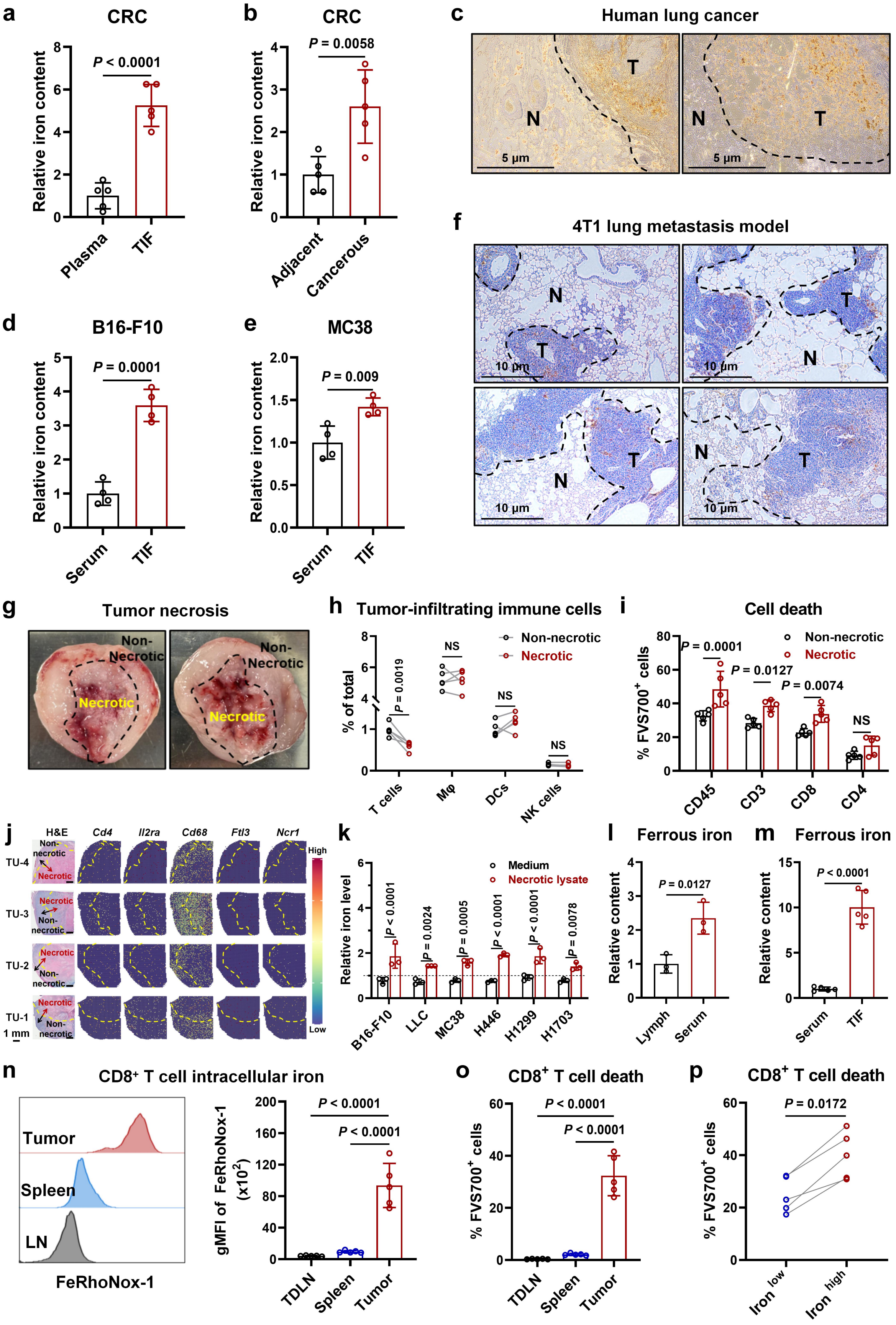

Supplementary Fig. 1 | Tumor necrosis-induced iron-overloaded TME correlates with CD8⁺ T cell exhaustion. a, **b** Relative iron content of plasma and

paired TIF (**a**), interstitial fluid from cancerous and adjacent non-cancerous tissues (**b**) from CRC patients (n = 5 patients). **c** Prussian blue staining on lung cancer tissue sections from 2 patients. Brown deposits: iron enrichment. Dashed line: tumor (T) and adjacent normal (N). **d**, **e** Relative iron content in serum and paired TIF from B16-F10 and MC38 tumor-bearing mice (n = 4 mice). **f** Prussian blue staining on lung sections from 4 mice with 4T1 lung metastasis. **g**–**i** Characterization of necrotic and non-necrotic regions in LLC tumors (n = 5 mice). **g** Demarcation of necrotic and non-necrotic regions. **h** Infiltration frequencies of T cells, macrophages (Mφ), dendritic cells (DCs), and natural killer (NK) cells. **i** Corresponding cell death rates of infiltrating $CD45^+$, $CD3^+$, $CD4^+$, and $CD8^+$ cells. **j** H&E scans and spatial transcriptomic maps showing the distribution of various immune cell populations, including $CD4^+$ T cells, regulatory T cells (Tregs), Mφ, DCs, and NK cells, in necrotic and non-necrotic regions of 4T1 tumors (n = 4 mice). **k** Relative iron content in necrotic lysates and their corresponding conditioned media from human and murine tumor cells compared to fresh culture medium (n = 3 biologically independent samples). **l** Relative labile iron content in serum and lymph from Sprague-Dawley rats (n = 3 rats). **m** Relative labile iron content in paired serum and TIF samples from C57BL/6 mice bearing B16 tumors (n = 5 mice). **n**–**p** Analysis of $CD8^+$ T cells from LLC tumor-bearing mice (n = 5 mice). Intracellular iron levels (**n**) and cell death (**o**) in $CD8^+$ T cells derived from tumors, spleens, and TDLNs. **p** Cell death comparison in $CD8^+$ TILs stratified into high (top 50%) and low (bottom 50%) iron-loading subgroups based on FeRhoNox-1 gMFI. Data are shown as mean ± SD. *P* values were calculated using unpaired two-tailed t test (**a**, **b**, **d**, **e**, **h**, **l**, **m**, **p**); two-way ANOVA (**i**, **k**); one-way ANOVA (**n**, **o**). Source data are provided as a Source Data file.

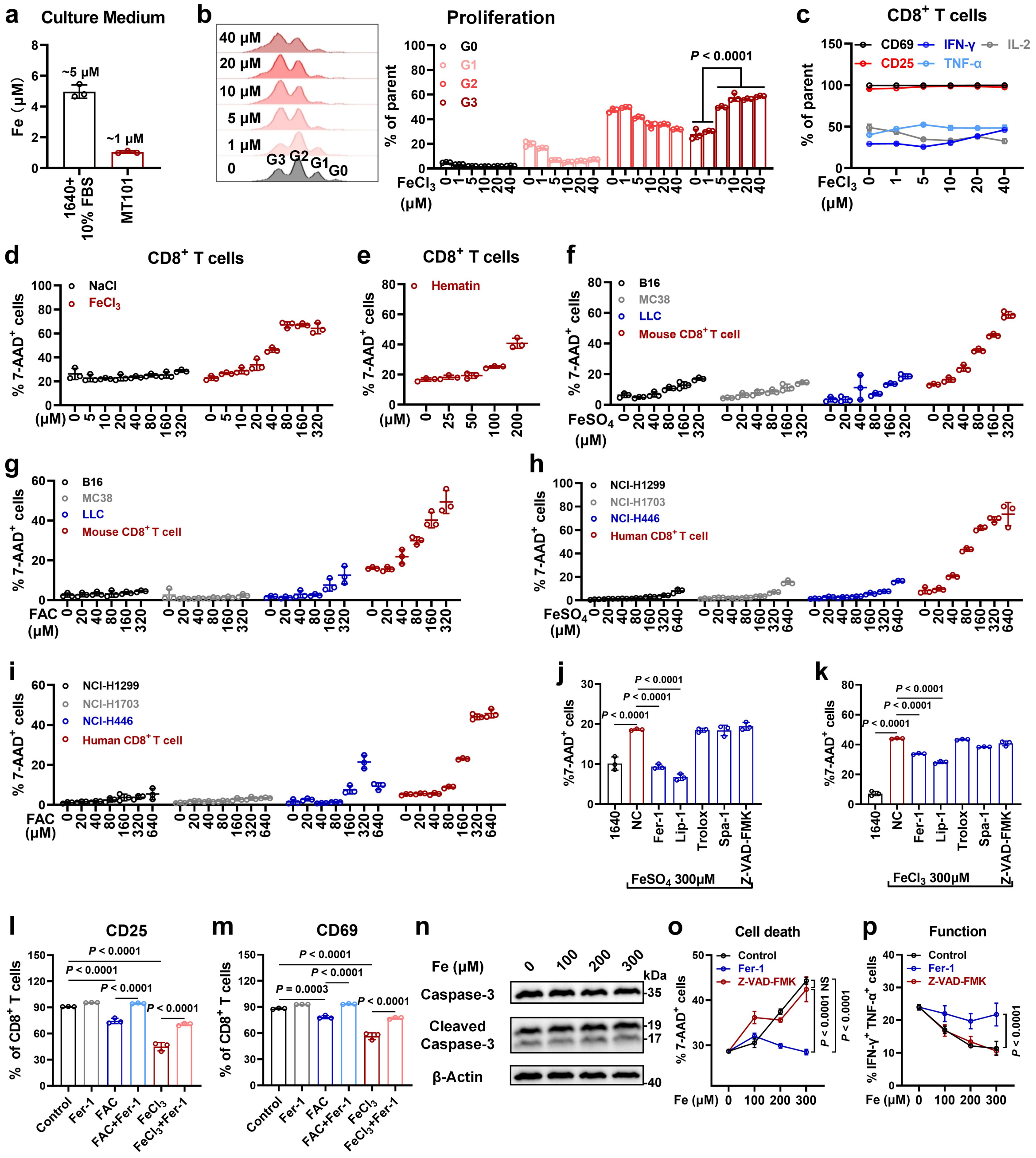

Supplementary Fig. 2 | Iron overload drives CD8⁺ T cell dysfunction via ferroptosis. a Iron concentration in RPMI-1640 base medium, complete RPMI-1640 medium (supplemented with 10% FBS), and iron-deficient, serum-free MT101 medium (n = 3 batches). **b**, **c** $CD8^+$ T cells were activated in MT101 medium supplemented with iron at physiological concentrations (n = 3 biologically independent samples). **b** Cell proliferation. **c** Expression of CD25 and CD69, and production of IFN-γ, TNF-α, and IL-2. **d**, **e** Cell death of $CD8^+$ T cells treated for 24 h with $FeCl_3$ and NaCl; or hematin during activation (n = 3 biologically independent samples). **f**, **g** Cell death of murine $CD8^+$ T cells and the indicated tumor cell lines after 24-h treatment with $FeSO_4$ or FAC (n = 3 biologically independent samples). **h**, **i** Cell death of human $CD8^+$ T cells and the indicated tumor cell lines after 24-h treatment with $FeSO_4$ or FAC (n = 3 biologically independent samples). **j**, **k** Cell

death of activated murine CD8$^+$ T cells treated with $FeSO_4$ (300 μM) or $FeCl_3$ (300 μM) in combination with Fer-1 (1 μM), Lip-1 (1 μM), Trolox (20 μM), Spa-1 (5 μM), or Z-VAD-FMK (5 μM) for 48 h (n = 3 biologically independent samples). **l, m** Activation marker CD25 and CD69 expression on activated CD8$^+$ T cells treated with FAC (200 μM) or $FeCl_3$ (200 μM) ± Fer-1 (1 μM) for 48 h (n = 3 biologically independent samples). **n–p** Iron overload does not induce apoptosis in murine CD8$^+$ T cells (n = 3 biologically independent samples). **n** Cleaved caspase-3 expression in cells treated with $FeCl_3$ for 48 h. **o**, **p** CD8$^+$ T cells were treated with increasing concentrations of $FeCl_3$ ± Fer-1 or Z-VAD-FMK for 48 h. **o** Cell death. **p** Frequencies of IFN-γ$^+$ and TNF-α$^+$ cells were determined by intracellular cytokine staining following restimulation. Data are shown as mean ± SD. *P* values were calculated using two-way ANOVA (**b**, **o**, **p**); one-way ANOVA (**j**–**m**). Source data are provided as a Source Data file.

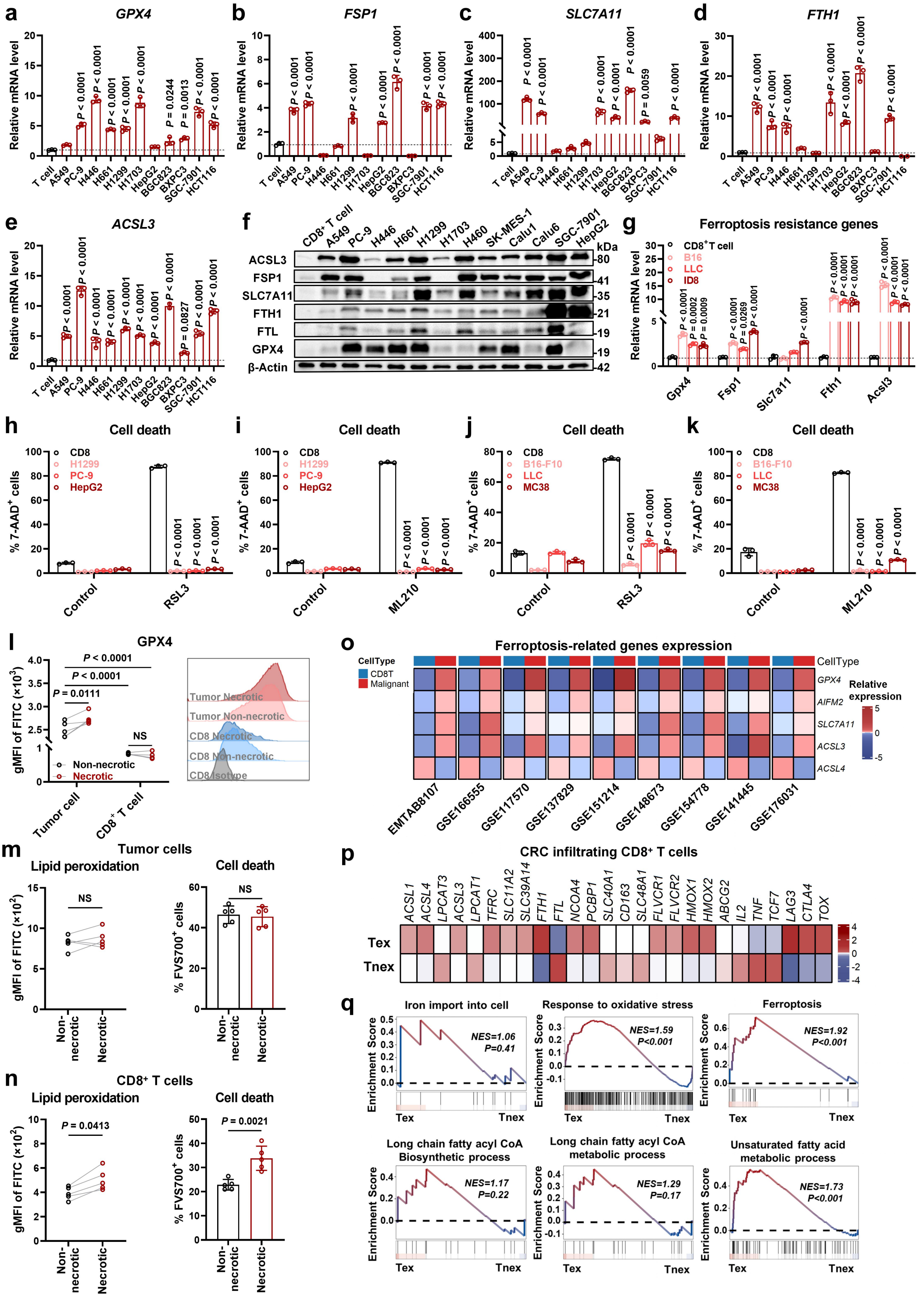

Supplementary Fig. 3 | Tumor cells exhibit enhanced ferroptosis resistance compared to CD8⁺ T cells. a–**e** mRNA expression levels of ferroptosis resistance genes, including *GPX4*, *FSP1*, *SLC7A11*, *FTH1*, and *ACSL3*, in human CD8⁺ T cells and human tumor cell lines (n = 3 biologically independent samples). **f**

Representative western blot (n = 3 independent experiments) of GPX4, FSP1, SLC7A11, FTH1, and ACSL3 in human $CD8^+$ T cells and human tumor cell lines. **g** mRNA expression levels of ferroptosis resistance genes, including *Gpx4*, *Fsp1*, *Slc7a11*, *Fth1*, and *Acsl3*, in murine $CD8^+$ T cells and murine tumor cell lines (n = 3 biologically independent samples). **h**–**k** Cell death of human (**h**, **i**) and murine (**j**, **k**) $CD8^+$ T cells and tumor cell lines treated with ferroptosis inducers for 24 h: 100 nM RSL3 (**h**), 1 μM ML210 (**i**), 200 nM RSL3 (**j**), or 2 μM ML210 (**k**) (n = 3 biologically independent samples). **l**–**n** Comparative analysis of tumor cells and $CD8^+$ T cells in necrotic versus non-necrotic regions of LLC tumors (n = 5 mice). **l** GPX4 expression levels in tumor cells and $CD8^+$ T cells from necrotic and non-necrotic regions. **m** Lipid peroxidation and cell death in tumor cells. **n** Lipid peroxidation and cell death in $CD8^+$ T cells. **o** Comparison of *GPX4*, *AIFM2* (*FSP1*), *SLC7A11*, *ACSL3*, and *ACSL4* expression levels between malignant cells and $CD8^+$ T cells in public single-cell RNA-sequencing datasets from human cancers (n = 75 patients from 9 datasets). **p**, **q** Analysis of public single-cell RNA-sequencing data from CRC (n = 6 patients). **p** Expression levels of key genes related to ferroptosis, iron metabolism, and T cell function across exhausted (Tex) and non-exhausted (Tnex) $CD8^+$ T cell clusters. **q** GSEA enrichment for ferroptosis and iron metabolism pathways in the indicated clusters. Data are shown as mean ± SD. *P* values were calculated using one-way ANOVA (**a**–**e**); two-way ANOVA (**g**–**l**); unpaired two-tailed t test (**m**, **n**). Source data are provided as a Source Data file.

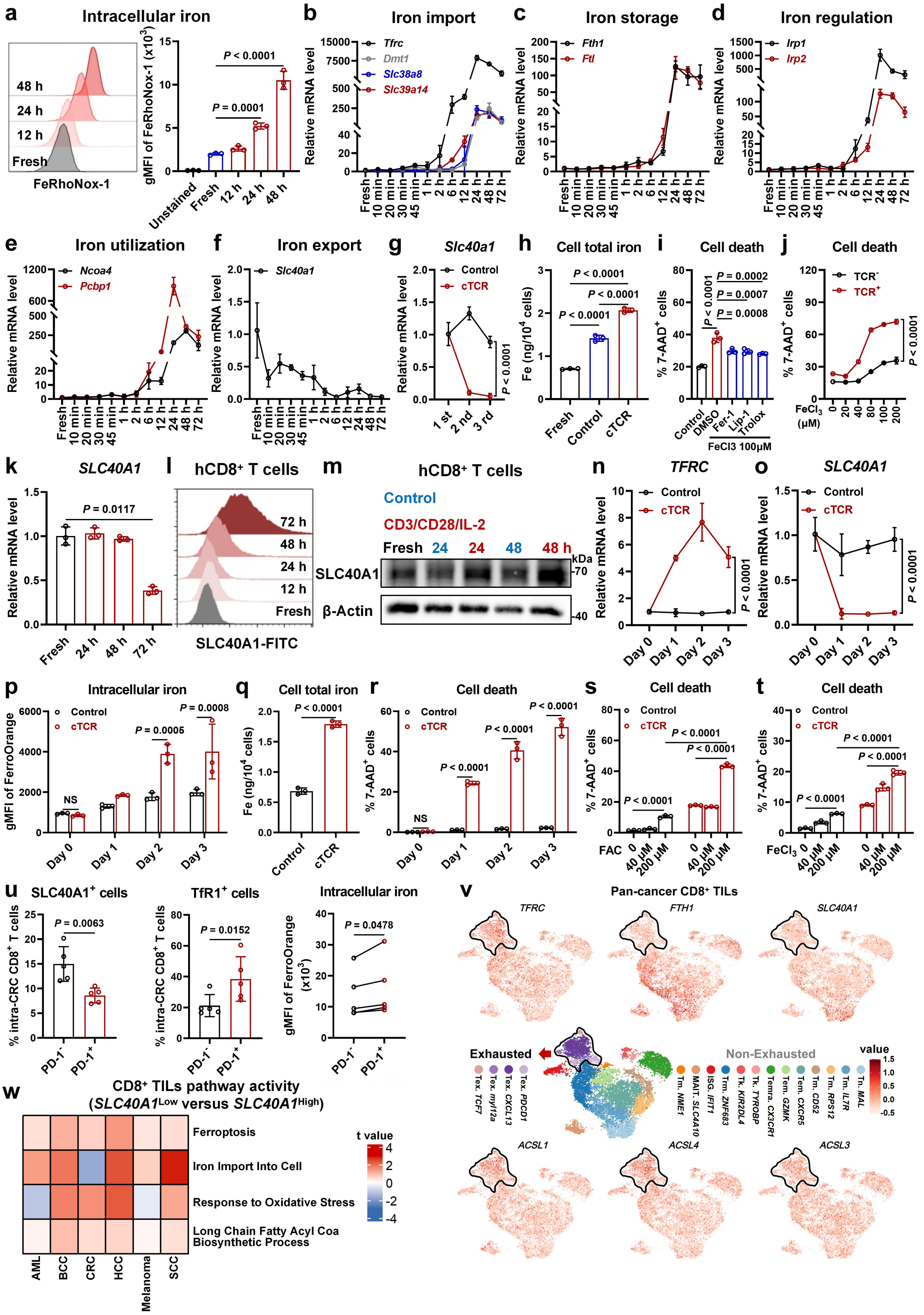

Supplementary Fig. 4 | SLC40A1 suppression causes iron overload and ferroptosis in CD8⁺ TILs. a–f Iron metabolism during murine CD8⁺ T cell activation (n = 3 biologically independent samples). **a** Intracellular iron. **b–f** mRNA dynamics of iron import, storage, regulation, utilization, and export genes. **g**

Slc40a1 mRNA expression in murine $CD8^+$ T cells maintained with IL-2 (10 ng/mL) or subjected to chronic TCR (cTCR) stimulation (n = 3 biologically independent samples). **h** Total intracellular iron content in fresh, control, and cTCR-stimulated $CD8^+$ T cells (n = 3 biologically independent samples). **i** Cell death of murine $CD8^+$ T cells treated with $FeCl_3$ (100 μM) ± Fer-1 (1 μM), Lip-1 (1 μM), or Trolox (20 μM) for 48 h (n = 3 biologically independent samples). **j** Cell death of resting versus TCR-stimulated $CD8^+$ T cells treated with $FeCl_3$ for 48 h (n = 3 biologically independent samples). **k**–**m** SLC40A1 expression during human $CD8^+$ T cell activation (n = 3 biologically independent samples). mRNA (**k**) and surface protein (**l**) expression levels. **m** protein levels in IL-7-maintained naïve versus activated cells. **n**–**r** Iron metabolism of human $CD8^+$ T cell during cTCR stimulation (n = 3 biologically independent samples). **n**, **o** *TFRC* and *SLC40A1* mRNA expression. **p**–**r** Intracellular iron, cell total iron, and cell death. **s**, **t** Cell death of human $CD8^+$ T cell treated with FAC or $FeCl_3$ during cTCR stimulation (n = 3 biologically independent samples). **u** SLC40A1, TfR1, and intracellular iron levels in exhausted ($PD\text{-}1^+$) versus non-exhausted ($PD\text{-}1^-$) $CD8^+$ T cell subsets from CRC patients (n = 5 patients). **v** t-SNE plot from pan-cancer single-cell RNA sequencing showing *TFRC*, *FTH1*, *SLC40A1*, *ACSL1*, *ACSL4*, and *ACSL3* expression in exhausted versus non-exhausted $CD8^+$ T cell populations (n = 316 patients from 21 cancer types). **w** Pathway activities differences, scored per cell by Gene Set Variation Analysis (GSVA), between $SLC40A1^{low}$ and $SLC40A1^{high}$ $CD8^+$ TILs across cancer types (n = 79 patients from 6 datasets). T values are from a linear model. Data are shown as mean ± SD. *P* values were calculated using one-way ANOVA (**a**, **h**, **i**, **k**); two-way ANOVA (**g**, **j**, **n**–**p**, **r**–**t**); unpaired two-tailed t test (**q**); paired two-tailed t test (**u**). Source data are provided as a Source Data file.

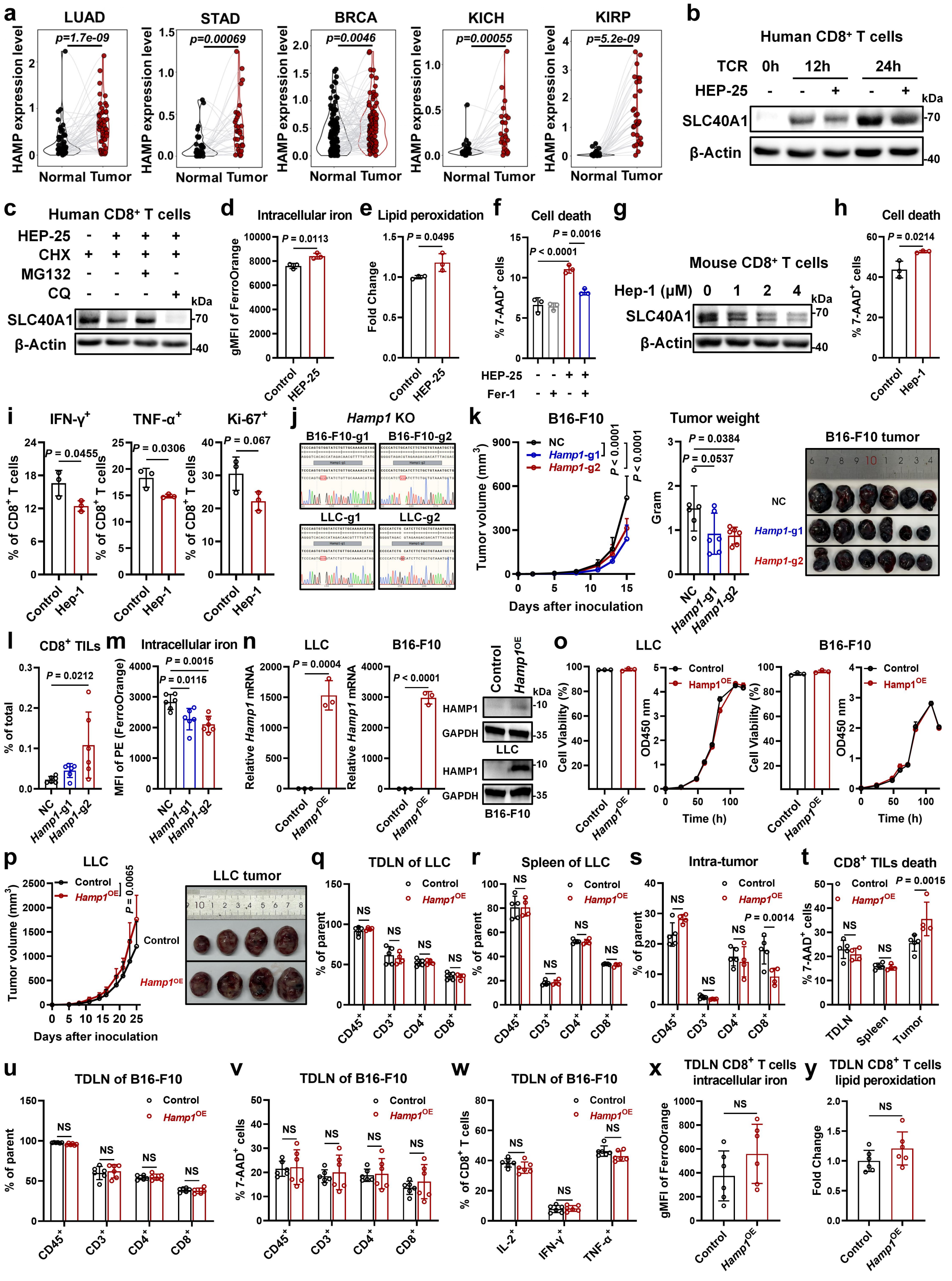

Supplementary Fig. 5 | Cancer upregulates hepcidin to suppress SLC40A1 in CD8⁺ T cells. a *HAMP* mRNA expression in tumor versus adjacent normal tissues from the TCGA database for lung adenocarcinoma (LUAD, n = 58 patients), stomach adenocarcinoma (STAD, n = 33 patients), breast invasive carcinoma (BRCA, n = 113 patients), kidney chromophobe (KICH, n = 25 patients), and kidney renal papillary cell carcinoma (KIRP, n = 32 patients). **b**, **c** Representative

western blots (n = 3 independent experiments) of SLC40A1 protein levels in human CD8+ T cells. **b** Cells treated with 500 nM HEP-25 during activation. **c** Cells treated with HEP-25 (500 nM) ± CHX (75 μM), MG132 (10 μM), or CQ (50 μM) for 4 h. **d**, **e** Intracellular iron and lipid peroxidation of $CD8^+$ T cells treated with HEP-25 (1 μM) for 48 h (n = 3 biologically independent samples). **f** Cell death of $CD8^+$ T cells treated with HEP-25 (1 μM) ± Fer-1 (1 μM) for 48 h (n = 3 biologically independent samples). **g** Representative western blots (n = 3 independent experiments) of SLC40A1 protein levels in murine $CD8^+$ T cells treated with Hep-1 for 48 h. **h**, **i** Cell death and functional impairment of cells treated with Hep-1 (1 μM) for 48 h (n = 3 biologically independent samples). **j** Verification of *Hamp1*-knockout cell lines by sequencing (at least 3 times). **k**–**m** Control or *Hamp1*-knockout B16-F10 cells (2×10^5) were injected subcutaneously into C57BL/6 mice (n = 6 mice). **k** Tumor growth curves, weights, and images. **l**, **m** Proportions and intracellular iron of $CD8^+$ TILs. **n** Validation of *Hamp1*-overexpressing (*Hamp1*^{OE}) cell lines (n = 3 biologically independent samples). **o** *In vitro* viability and proliferation of control and *Hamp1*^{OE} LLC and B16-F10 cell lines (n = 3 biologically independent samples). **p**–**t** Control or *Hamp1*^{OE} LLC cells (5×10^5) were injected subcutaneously into C57BL/6 mice (Control, n = 4; *Hamp1*^{OE}, n = 5). **p** Tumor growth curves and images. **q**–**t** Proportions of $CD45^+$, $CD3^+$, $CD4^+$, and $CD8^+$ cells and cell death of $CD8^+$ T cells in TDLNs, spleens and tumors. **u**–**y** Proportions and cell death of $CD45^+$, $CD3^+$, $CD4^+$, and $CD8^+$ immune cells, and functional capacity, intracellular iron and lipid peroxidation of $CD8^+$ T cells in TDLNs of B16-F10 *Hamp1*^{OE} syngeneic model (n = 6 mice). Data are shown as mean ± SD. *P* values were calculated using unpaired two-tailed t test (**d**, **e**, **h**, **i**, **n**, **x**, **y**); one-way ANOVA (**f**, tumor weight in **k**, **l**, **m**); two-way ANOVA (tumor growth curves in **k**, **p**–**w**). Source data are provided as a Source Data file.

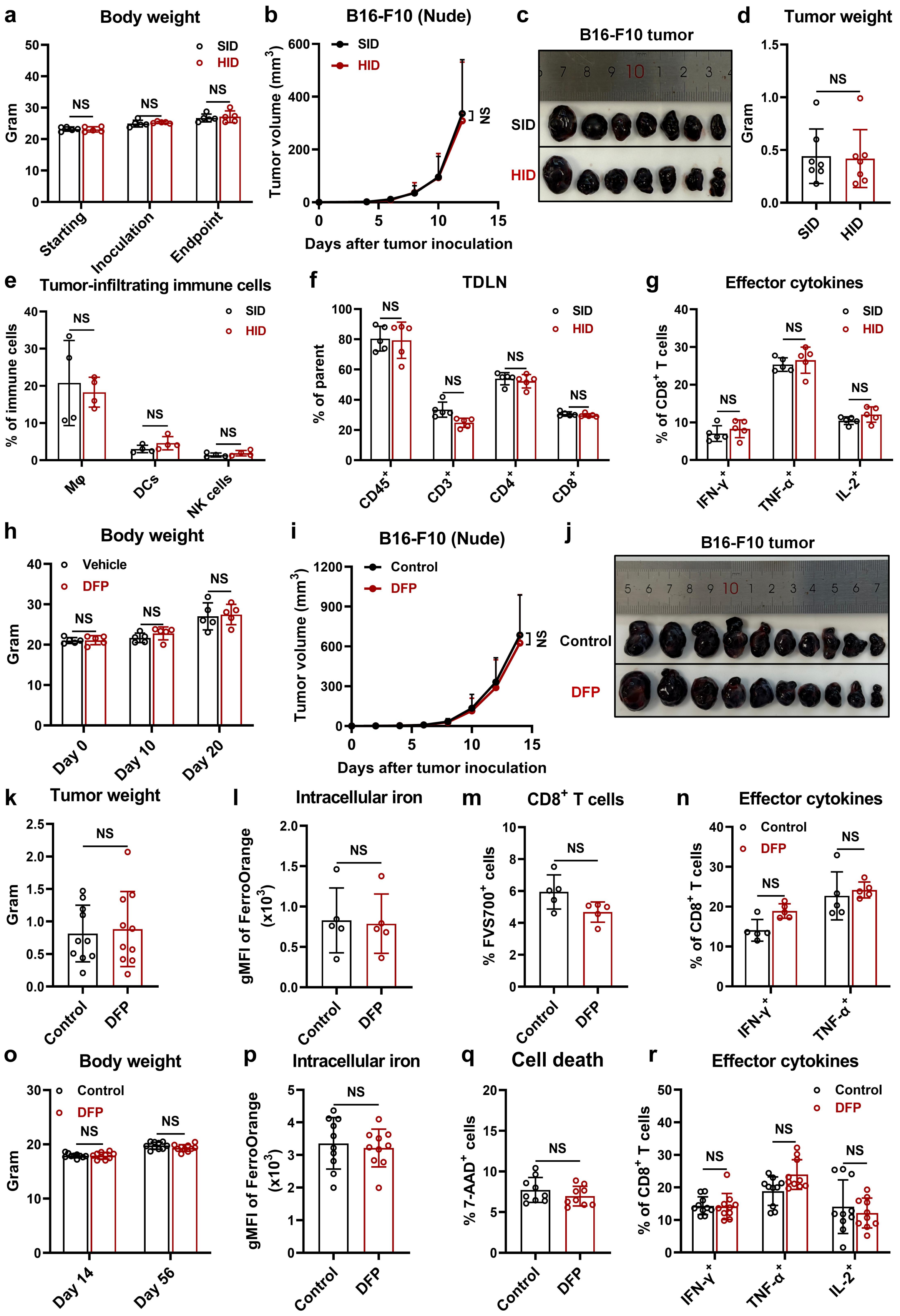

Supplementary Fig. 6 | Iron orchestration reprograms CD8⁺ T cell anti-tumor immunity. a–g Characterization of the B16-F10 tumor model under HID and SID. **a** Body weights of C57BL/6 mice fed a HID or SID at starting point, tumor

inoculation, and endpoint (n = 5 mice). **b–d** Tumor growth curves, images and weights of T cell-deficient nude mice fed a HID or SID (n = 7 mice). **e** Proportions of tumor-infiltrating macrophages (Mφ), dendritic cells (DCs), and natural killer (NK) cells in C57BL/6 mice fed HID or SID (n = 4 mice). **f** Proportions of $CD45^+$, $CD3^+$, $CD4^+$, and $CD8^+$ immune cells in TDLNs (n = 5 mice). **g** Effector function (IFN-γ, TNF-α, and IL-2 production) of $CD8^+$ T cells in TDLNs (n = 5 mice). **h–n** Characterization of the B16-F10 model with DFP treatment. **h** Body weights of control and DFP-treated C57BL/6 mice on days 0, 10, and 20 (n = 5 mice). **i–k** Tumor growth curves, images and weights of T cell-deficient nude mice treated with control or DFP (n = 10 mice). **l–n** Analysis of $CD8^+$ T cells from TDLNs for intracellular iron levels, cell death, and effector function (n = 5 mice). **o–r** Characterization of the ID8 model with DFP treatment (n = 10 mice). **o** Body weights of control and DFP-treated mice on days 14 and 56. **p–r** Analysis of $CD8^+$ T cells from TDLNs for intracellular iron levels, cell death, and effector function. Data are shown as mean ± SD. *P* values were calculated using two-way ANOVA (**a**, **b**, **e–i**, **n**, **o**, **r**); unpaired two-tailed t test (**d**, **k–m**, **p**, **q**). Source data are provided as a Source Data file.

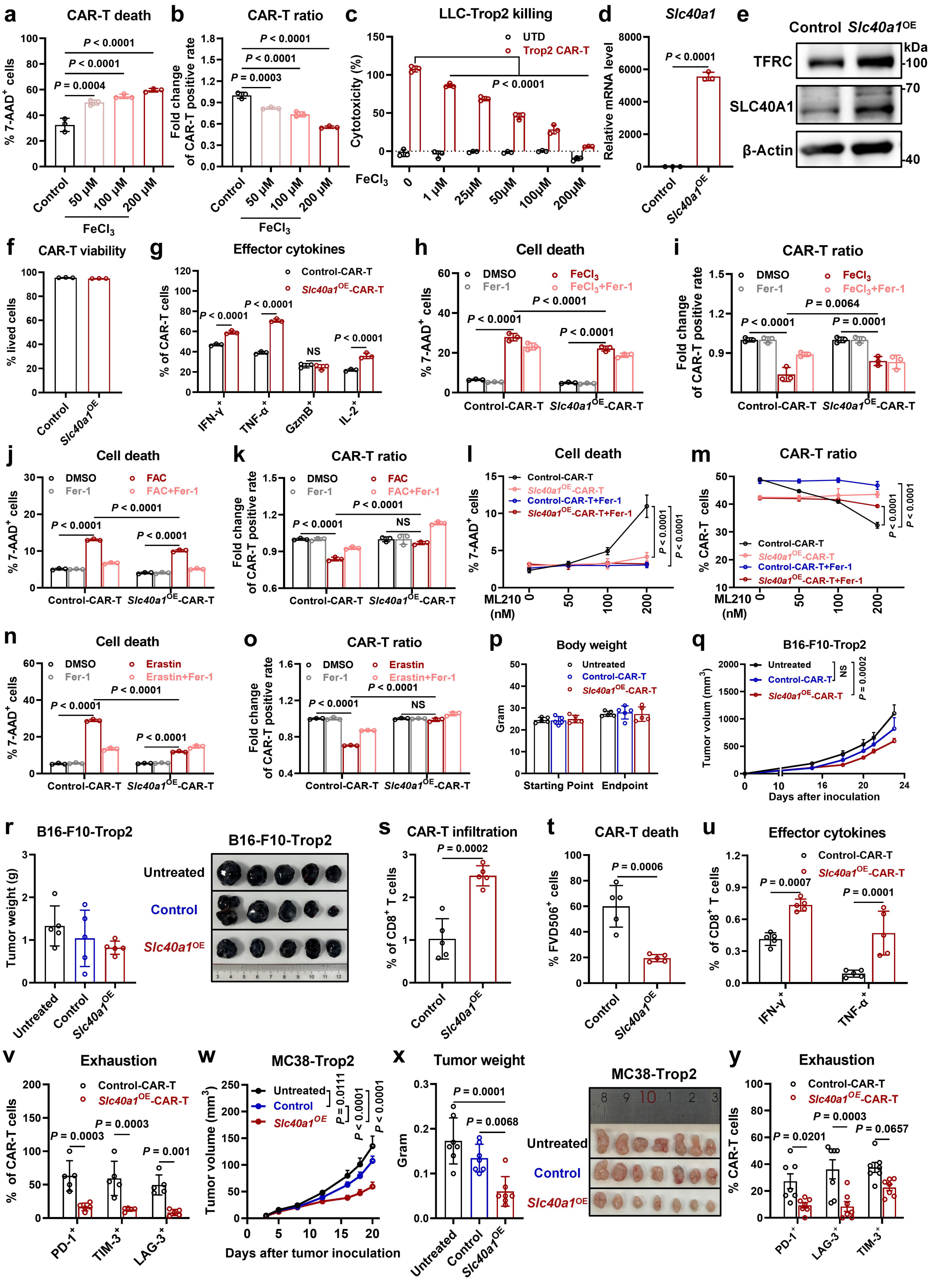

Supplementary Fig. 7 | *Slc40a1*^{OE}-CAR-T cells resist the iron-overloaded TME to potentiate anti-tumor immunity. a Cell death of CAR-T cells treated for 24 h with $FeCl_3$ (n = 3 biologically independent samples). **b**, **c** Proportion of surviving and target cell killing of CAR-T cells co-cultured with LLC-Trop2 (E:T ratio = 1:2) in the presence of $FeCl_3$ for 24 h (n = 3 biologically independent samples). **d**, **e**

Validation of *Slc40a1* overexpressing (*Slc40a1*^{OE}) CAR-T cells (n = 3 biologically independent samples). **f**, **g** Cell viability and effector function of *Slc40a1*^{OE} CAR-T cells under resting conditions (n = 3 biologically independent samples). **h**–**o** Cell death and ratio of CAR-T cells treated for 24 h with indicated compounds (n = 3 biologically independent samples). **h**, **i** $FeCl_3$ (200 μM) ± Fer-1 (1 μM). **j**, **k** FAC (200 μM) ± Fer-1 (1 μM). **l**, **m** ML210 (0, 50, 100, 200 nM) ± Fer-1 (1 μM). **n**, **o** Erastin (20 μM) ± Fer-1 (1 μM). **p**–**v** C57BL/6 mice were subcutaneously inoculated with 5 × 10^5 B16-F10-Trop2 cells on day 0, received CTX (80 mg/kg, i.p.) on day 4 for lymphodepletion, and were treated with 1 × 10^6 CAR-T cells per mouse via tail vein injection on day 7 (n = 5 mice). **p** Mouse body weight. **q**, **r** Tumor growth curves, images and weights. **s**–**v** Persistence, cell death, effector function and exhaustion of tumor-infiltrating CAR-T cells. **w**–**y** C57BL/6 mice were subcutaneously inoculated with 1 × 10^6 MC38-Trop2 cells on day 0, received CTX (80 mg/kg, i.p.) on day 4 for lymphodepletion, and were treated with 1 × 10^6 CAR-T cells per mouse via tail vein injection on day 7 (n = 7 mice). **w**, **x** Tumor growth curves, images and weights. **y** Expression of exhaustion markers (PD-1, TIM-3, and LAG-3) on tumor-infiltrating CAR-T cells. Data are shown as mean ± SD except **q**, **w**, **y** (mean ± SEM). *P* values were calculated using one-way ANOVA (**a**, **b**, **r**, **x**); two-way ANOVA (**c**, **g**–**q**, **u**–**w**, **y**); unpaired two-tailed t test (**d**, **f**, **s**, **t**). Source data are provided as a Source Data file.

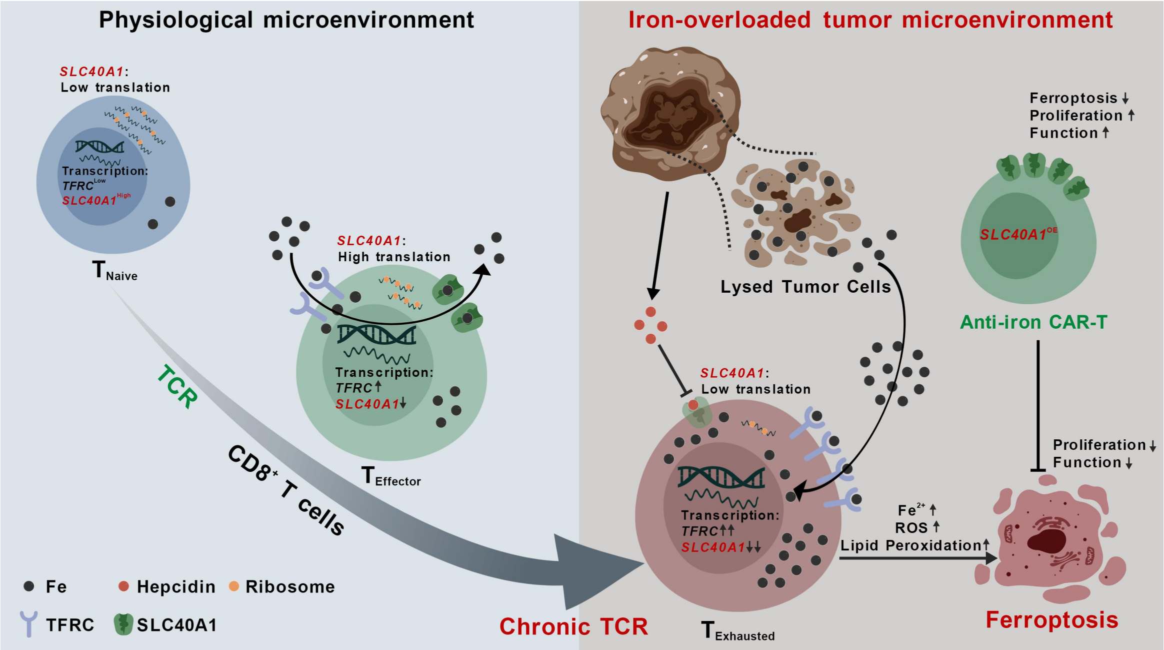

Supplementary Fig. 8 | Schematic Figure: Iron overload in the tumor microenvironment induces CD8$^+$ T cell ferroptosis and dysfunction. Created with BioGDP

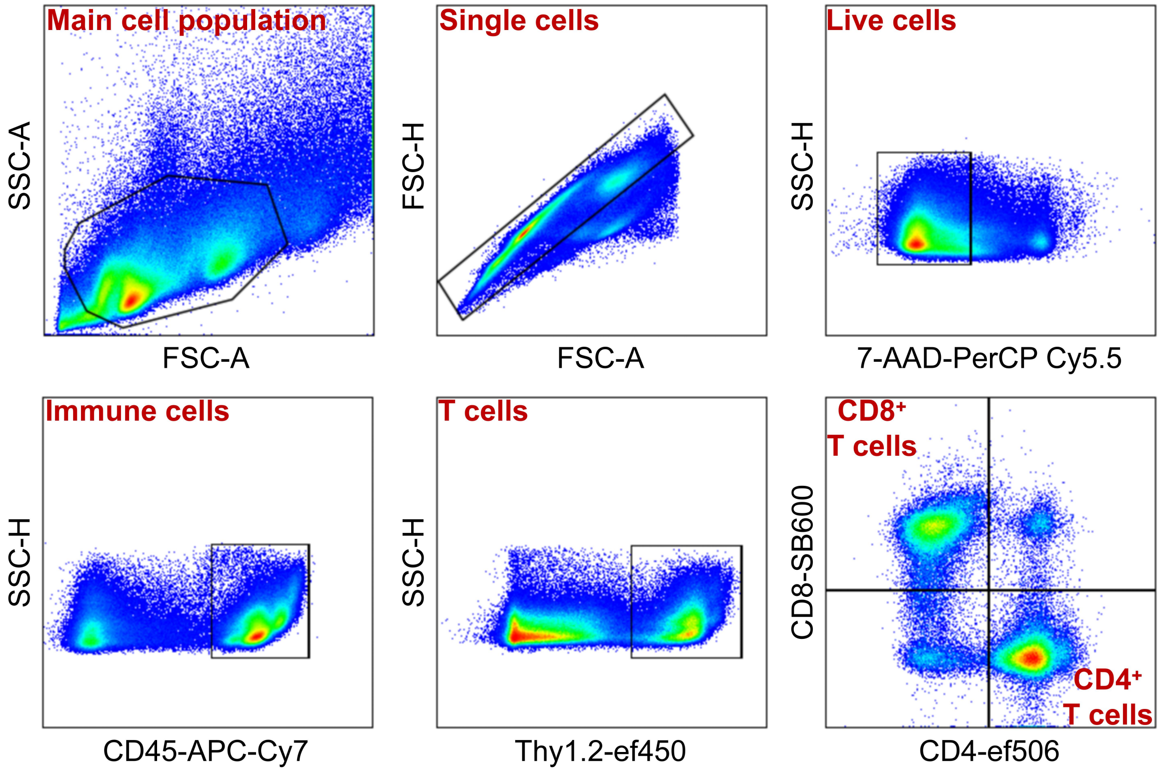

Supplementary Fig. 9 | Representative gating strategy for flow cytometry. Sequential gating used to identify CD8⁺ T cell subsets and markers. The same gating strategy was applied to the FACS data in **Fig. 1j**, **k**, **p–t**; **Fig. 2p**; **Fig. 3m**, **n**; **Fig. 4j–o**, **r–z**; **Fig. 5d–g**, **l–o**, **t–x**; **Fig. 6a–e**, **h**, **i**, **u–y**; **Supplementary Fig. 1i**, **n–p**; **Supplementary Fig. 3l–n**; **Supplementary Fig. 5l**, **m**, **q–y**; **Supplementary Fig. 6e–g**, **l–n**, **p–r**; **Supplementary Fig. 7s–v**, **y**.

Supplementary Fig. 10 | Uncropped scans of all blots and gels shown in the supplementary figures.

Supplementary Fig. 2n

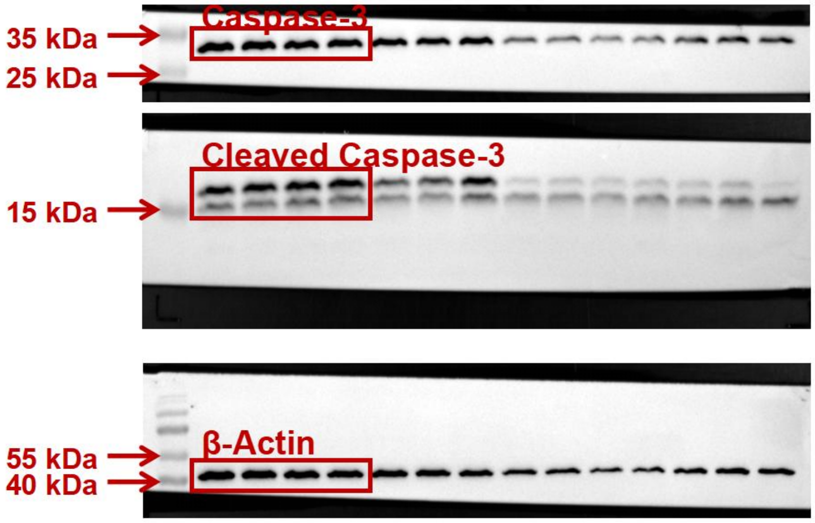

Supplementary Fig. 3f

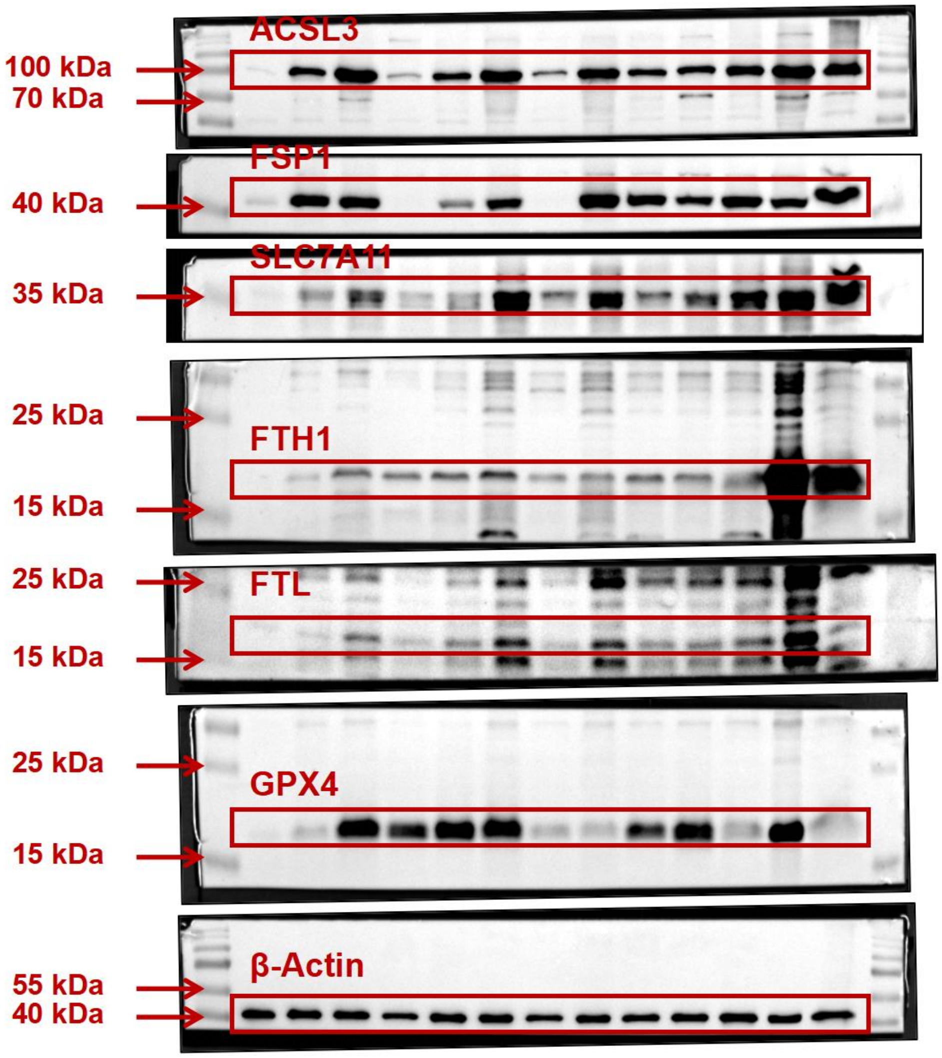

Supplementary Fig. 4m

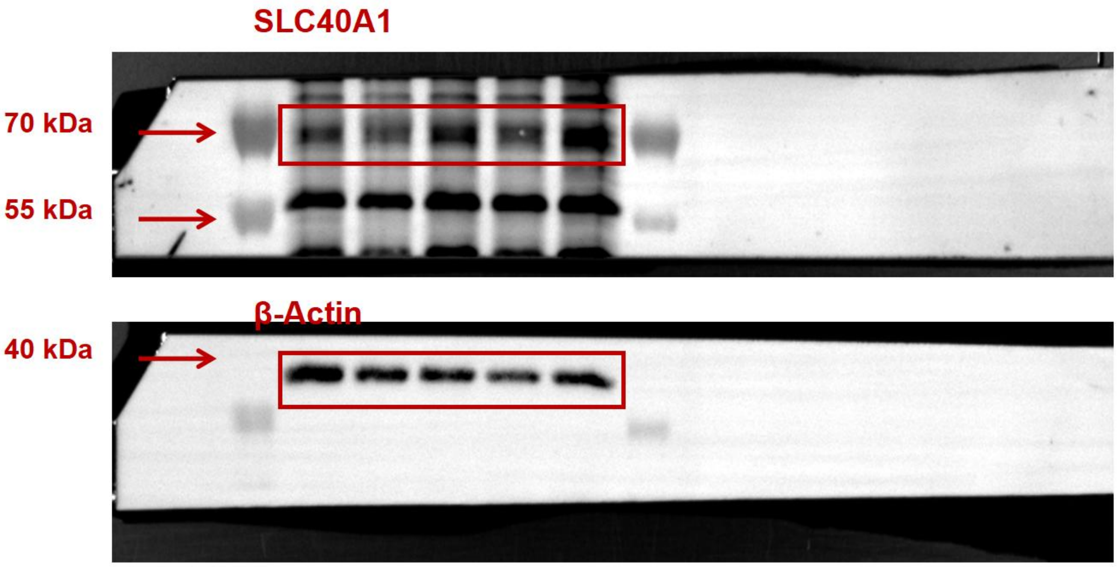

Supplementary Fig. 5b

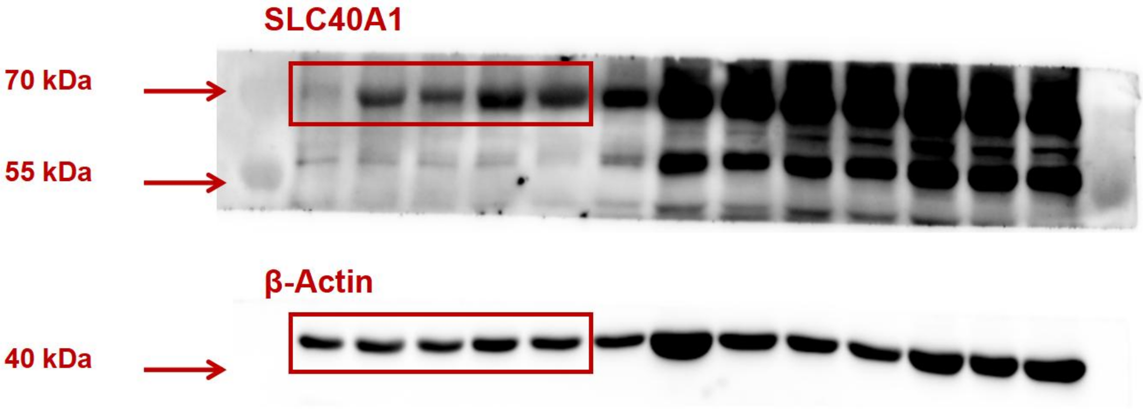

Supplementary Fig. 5c

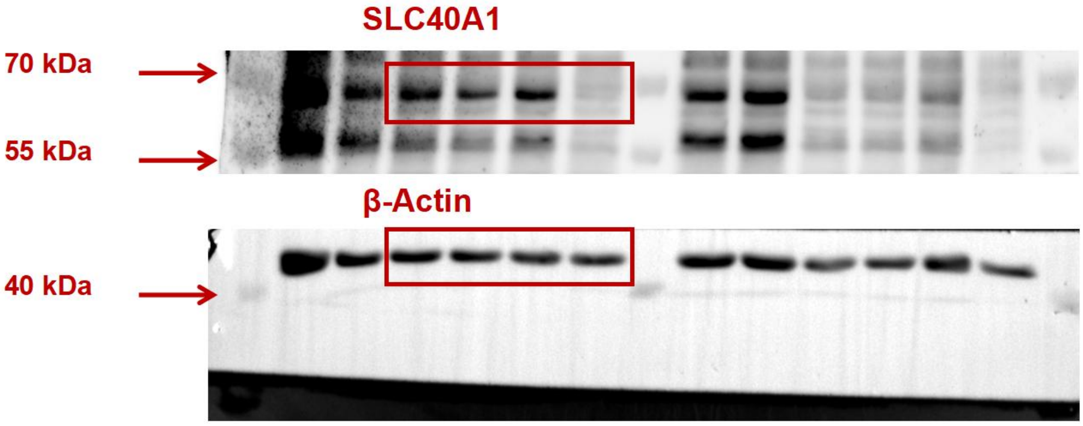

Supplementary Fig. 5g

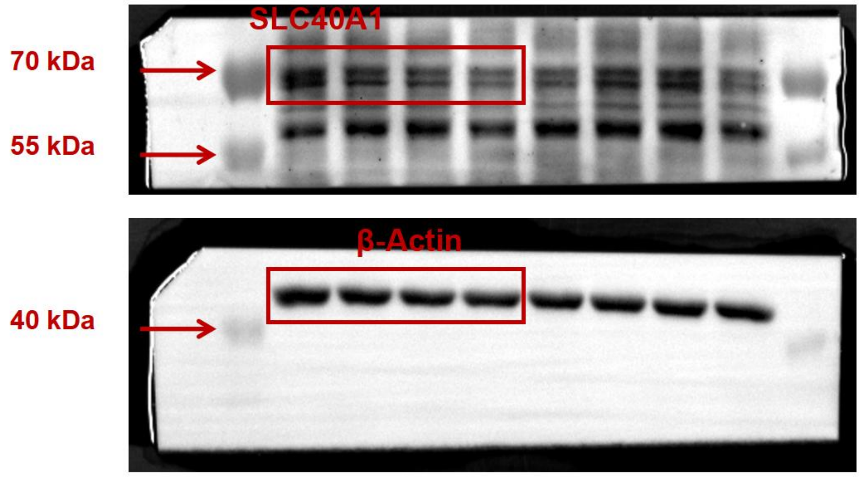

Supplementary Fig. 5n

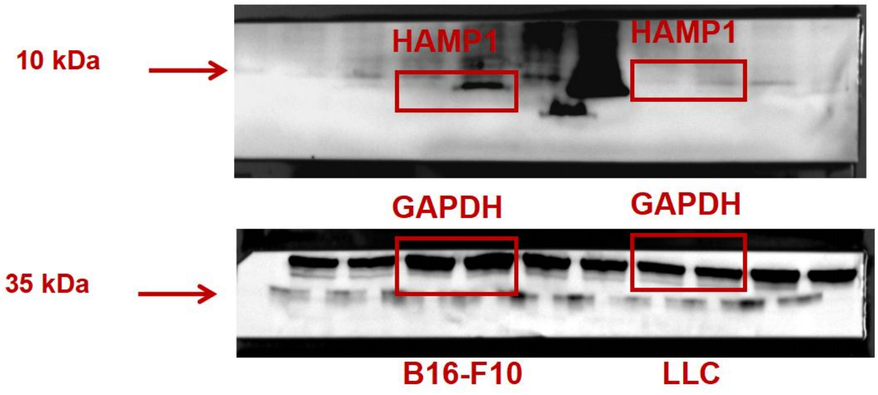

Supplementary Fig. 7e

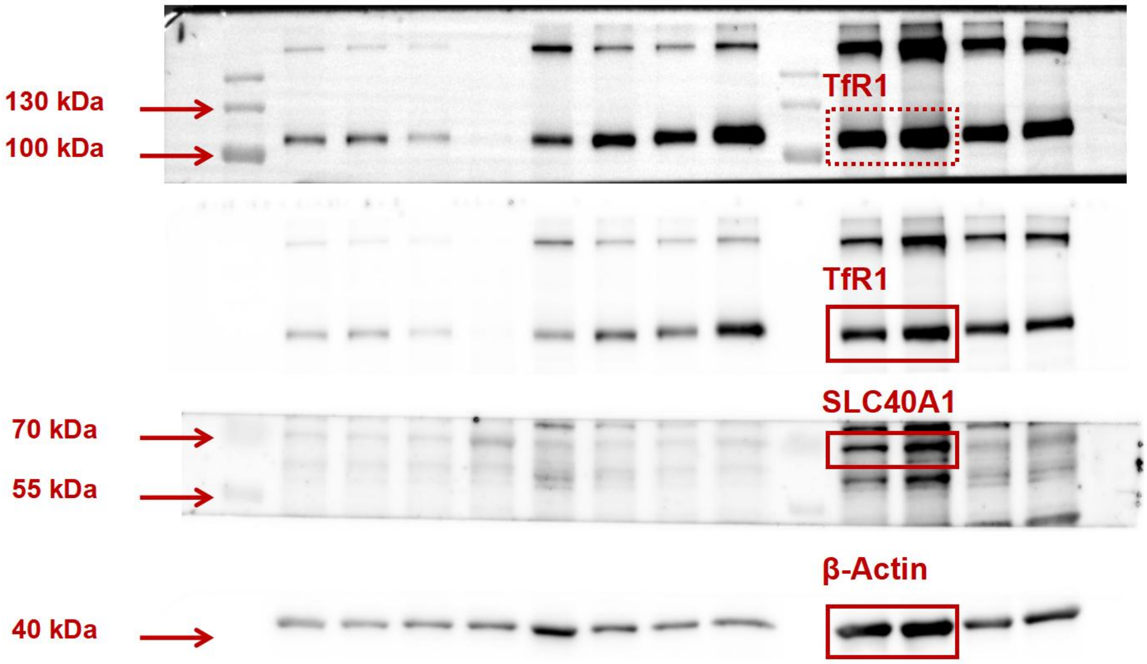

Supplementary Table 1 | Clinical and pathological characteristics of the patient cohorts.

Patient ID	Diagnosis	Average Age	Sex (M/F)	Disease Stage
**Clinical characteristics of lung cancer patients with malignant pleural effusion**				
LC001	Adenocarcinoma	67	M: 4 F: 4	cT4N2M1
LC002	Squamous Cell Carcinoma			cT3N3M1
LC003	Squamous Cell Carcinoma			cT4N2aM1
LC004	NA			cT4N3M1
LC005	Adenocarcinoma			cT4N2bM1
LC006	Adenocarcinoma			cT4N3M1
LC007	Adenocarcinoma			cT4N2bM1
LC008	Adenocarcinoma			cT4N2bM1
**Clinical characteristics of lung cancer patients with tissue sections**				
LC009	Adenocarcinoma	55	M: 4	pT2 aN0M0
LC010	Adenocarcinoma			pT1bN0M0
LC011	NA			pT2bN2M0
LC012	Squamous Cell Carcinoma			NA
**Clinical characteristics of colorectal cancer patients with tumor samples**				
CRC001	Adenocarcinoma	72.4	M: 3 F: 2	pT3N2bM1a
CRC002	Adenocarcinoma			pT3N1bM1a
CRC003	Adenocarcinoma			pT3N0M0
CRC004	Adenocarcinoma			pT3N0M0
CRC005	Adenocarcinoma			pT4aN1cM0

Supplementary Table 2 | gRNA sequences for *Hamp1* knockout cell lines construction.

Primer Name	Sequence
m*Hamp1*-g1	TTTTGCAACAGATACCACAC
m*Hamp1*-g2	TTTACAGCAGAAGATGCAGA

Supplementary Table 3 | qPCR primer sequences.

Primer Name	Sequence
h/m*18S-rRNA*-F	GCAATTATTCCCCATGAACG
h/m*18S-rRNA*-R	GGCCTCACTAAACCATCCAA
m*Tfrc*-F	GAAGTCCAGTGTGGGAACAGGT
m*Tfrc*-R	CAACCACTCAGTGGCACCAACA
m*Dmt1*-F	TTGCAGCGAGACTTGGAGTGGT
m*Dmt1*-R	GCTGAGCCAATGACTTCCTGCA
m*Slc39a8*-F	CACTGAGCCTAACGGACACATC
m*Slc39a8*-R	CACAAAGCGTGATCATCCAGGC
m*Slc39a14*-F	CTGGCTATTGGTGCCTCCTTCA
m*Slc39a14*-R	TGCCAGCATTGAGCAGGATGAC
m*Fth1*-F	GCCGAGAAACTGATGAAGCTGC
m*Fth1*-R	GCACACTCCATTGCATTCAGCC
m*Ftl*-F	CCTCGAGTTTCAGAACGATCGC
m*Ftl*-R	CCTGATTCAGGTTCTTCTCCATG
m*Irp1*-F	CCATCCGTGATGTTAGGAGCAG
m*Irp1*-R	GACAGGTAAGGCATGACTCCAC
m*Irp2*-F	AGAAACGGACCTGCTCTTCCCA
m*Irp2*-R	CCTCTGTCTCAATGCCACCAAC
m*Ncoa4*-F	TGCCATTGGTCTTCAGGCTCCT
m*Ncoa4*-R	CAGGCATCGCTGAAGAAACTGC
m*Pcbp1*-F	GGACAACACACCATTTCTCCGC
m*Pcbp1*-R	AGCCTTTCACCTCTGGAGAGCT
m*Slc40a1*-F	ACCAAGGCAAGAGATCAAACC
m*Slc40a1*-R	AGACACTGCAAAGTGCCACAT
m*Gapdh*-F	CATCACTGCCACCCAGAAGACTG
m*Gapdh*-R	ATGCCAGTGAGCTTCCCGTTCAG
m*Hamp1*-F	ACATTGCGATACCAATGCAG
m*Hamp1*-R	CACTGGGAATTGTTACAGCA
m*Gpx4*-F	CCTCTGCTGCAAGAGCCTCCC
m*Gpx4*-R	CTTATCCAGGCAGACCATGTGC
m*Fsp1*-F	GCGACCTTCAAGGACAACTTCC
m*Fsp1*-R	GCCAGGATAAGATGTGAGAAGGG
m*Slc7a11*-F	CTTTGTTGCCCTCTCCTGCTTC
m*Slc7a11*-R	CAGAGGAGTGTGCTTGTGGACA

Primer Name	Sequence
m*Acsl3*-F	GCGAGAAGGATTCCAAGACTGG
m*Acsl3*-R	GAAGAGTAGCCGATTCGGCATC
h*GPX4*-F	ACAAGAACGGCTGCGTGGTGAA
h*GPX4*-R	GCCACACACTTGTGGAGCTAGA
h*FSP1*-F	GACTCCTTCCACCACAATGTGG
h*FSP1*-R	CAGCACCATCTGGTTCTTCAGG
h*SLC7A11*-F	TCCTGCTTTGGCTCCATGAACG
h*SLC7A11*-R	AGAGGAGTGTGCTTGCGGACAT
h*ACSL3*-F	CTTTCTCACGGATGCCGCATTG
h*ACSL3*-R	CTGCTGCCATCAGTGTTGGTTTC
h*TFRC*-F	ATCGGTTGGTGCCACTGAATGG
h*TFRC*-R	ACAACAGTGGGCTGGCAGAAAC
h*SLC40A1*-F	CTACTTGGGGAGATCGGATGT
h*SLC40A1*-R	CTGGGCCACTTTAAGTCTAGC

Supplementary Table 4 | Accession codes and details of publicly available datasets.

Deposited Data	Accession code
Spatial transcriptomic analysis of 4T1 tumours	GEO datasets: GSE230098
scRNA-seq of Colorectal cancer (CRC)	ArrayExpress: EMTAB8107
scRNA-seq of CRC	GEO datasets: GSE166555
scRNA-seq of Non-small cell lung cancer (NSCLC)	GEO datasets: GSE117570
scRNA-seq of Prostate adenocarcinoma (PRAD)	GEO datasets: GSE137829
scRNA-seq of Ovarian cancer (OV)	GEO datasets: GSE151214
scRNA-seq of Pancreatic adenocarcinoma (PAAD)	GEO datasets: GSE148673
scRNA-seq of PAAD	GEO datasets: GSE154778
scRNA-seq of PRAD	GEO datasets: GSE141445
scRNA-seq of PRAD	GEO datasets: GSE176031
scRNA-seq of Lung cancer (LC)	GEO datasets: GSE127465
scRNA-seq of CRC	GEO datasets: GSE146771
scRNA-seq of Pan-Cancer	http://cancer-pku.cn:3838/PanC_T/
scRNA-seq of Acute myeloid leukemia (AML)	GEO datasets: GSE116256
scRNA-seq of Basal Cell Carcinoma (BCC)	GEO datasets: GSE123813
scRNA-seq of CRC	GEO datasets: GSE108989
scRNA-seq of Hepatocellular Carcinoma (HCC)	GEO datasets: GSE140228
scRNA-seq of Melanoma (MELA)	GEO datasets: GSE115978
scRNA-seq of Squamous Cell Carcinoma (SCC)	GEO datasets: GSE123813

Supplementary Table 5 | Key resources.

Resource	Source	Catalog Number
RhoNox-1	MedChemExpress	Cat# HY-D1533
FerroOrange	Dojindo	Cat# F374
Lipid Peroxidation Probe -BDP 581/591 C11	Dojindo	Cat# F267
BODIPY™ 665/676	Invitrogen	Cat# B3932
CM-H2DCFDA	Invitrogen	Cat# C6827
RSL3	TargetMol	Cat# T3646
ML210	TargetMol	Cat# T8375
Erastin	Sigma-Aldrich	Cat# E7781
Ferrostatin-1	MedChemExpress	Cat# HY-D1533
Liproxstatin-1	TargetMol	Cat# T2376
Trolox	TargetMol	Cat# T1710
Spautin-1	MedChemExpress	Cat# HY-12990
Z-VAD-FMK	MedChemExpress	Cat# HY-16658B
Sodium chloride	Acmec	Cat# S41330
Iron chloride hexahydrate	Aladdin	Cat# F102739
Sodium sulfate anhydrous	Aladdin	Cat# S112268
Iron sulfate heptahydrate	Aladdin	Cat# F116338
Iron (III) sulfate hydrate	Aladdin	Cat# I432161
Disodium fumarate	Boer	Cat# B607969
Iron (II) fumarate	Boer	Cat# B619056
Ammonium ferric citrate	Boer	Cat# B605372
Hematin	Sigma-Aldrich	Cat# H3281
Hemin	Sigma-Aldrich	Cat# 51280
3-Hydroxy-1,2-dimethyl-4(1H)-pyridone (Deferiprone)	Sigma-Aldrich	Cat# 379409
2-Mercaptoethanol	Sigma-Aldrich	Cat# M3148
Dimethyl sulfoxide	Sigma-Aldrich	Cat# D2650
Cyclophosphamide	Sigma-Aldrich	Cat# LEYH9ACEEAE7
CFSE	Invitrogen	Cat# C34555

Resource	Source	Catalog Number
Cycloheximide	MedChemExpress	Cat# HY-12320
MG-132	MedChemExpress	Cat# HY-13259
Chloroquine	MedChemExpress	Cat# HY-17589A
RNaseOUT Recombinant Ribonuclease Inhibitor	Invitrogen	Cat# 10777019
Brefeldin A	eBioscience	Cat# 00-4506-51
Monensin	eBioscience	Cat# 00-4505-51
Phorbol 12-myristate 13-acetate (PMA)	STEMCELL	Cat# 74042
Ionomycin	STEMCELL	Cat# 73722
Hepcidin-1	MedChemExpress	Cat# HY-P4373
Hepcidin-25	Synthesized by QYAOBIO	N/A
Mouse IL-2 Recombinant Protein	Peprotech	Cat# 212-12
Mouse IL-7 Recombinant Protein	Peprotech	Cat# 217-17
Human IL-2 Recombinant Protein	Peprotech	Cat# 200-02
Human IL-7 Recombinant Protein	Peprotech	Cat# 200-07
Serum Ferri Ion Content Assay Kit	Solarbio	Cat# BC1735
Prussian Blue Iron Stain Kit	Solarbio	Cat# G1428
Cell Iron Content Assay Kit	Solarbio	Cat# BC5310
Lipid Peroxidation Assay Kit	Abcam	Cat# ab243377
Cell Counting Kit-8	GLPBIO	Cat# GK10001
CytoTox 96®Non-Radioactive Cytotoxicity Assay	Promega	Cat# G1780
RevertAid First Strand cDNA Synthesis Kit	Thermo Fisher Scientific	Cat# K1621
EasySep Mouse $CD8^+$ T Cell Isolation Kit	STEMCELL	Cat# 19853
EasySep Human $CD8^+$ T Cell Isolation Kit	STEMCELL	Cat# 17953
DH5α chemically competent cell	AlpaLifeBio	Cat# KTSM101L
Stbl3 chemically competent cell	AlpaLifeBio	Cat# KTSM110L

Resource	Source	Catalog Number
Kanamycin Sulfate Storage Solution	Coolaber	Cat# SL3820
Ampicillin	Acmec	Cat# AC10038
2×Taq PCR StarMix (Dye)	GenStar	Cat# A012
Monoclone Genotype Validation Kit (extraction free)	Ubigene	Cat# YK-MV-1000
B16-F10	Newgainbio	Cat# CM3055
LLC	Newgainbio	Cat# CM3035
MC38	Cellverse	Cat# iCell-m032
4T1	Procell	Cat# CL-0007
ID8	Cellverse	Cat# iCell-m064
NCI-H446	Newgainbio	Cat# CH1106
NCI-H1299	Newgainbio	Cat# CH1075
NCI-H1703	Newgainbio	Cat# CH1089
HEK-293T	Newgainbio	Cat# CH1058
PC-9	Ubigene	Cat# YC-C114
HepG2	Newgainbio	Cat# CH1012
