# Supplementary material for: Iron overload in the tumor microenvironment induces CD8+ T cell ferroptosis and dysfunction
Source: Nat Commun. 2026 May 22;17:6754. doi: 10.1038/s41467-026-73379-4 (PMC13385831; doi:10.1038/s41467-026-73379-4)
Supplement: Supplementary file 2 — Reporting Summary [file 41467_2026_73379_MOESM2_ESM.pdf]

nature portfolio

Corresponding author(s): Yingjie Bian

Last updated by author(s): Apr 3, 2026

Reporting Summary

Nature Portfolio wishes to improve the reproducibility of the work that we publish. This form provides structure for consistency and transparency in reporting. For further information on Nature Portfolio policies, see our Editorial Policies and the Editorial Policy Checklist.

Statistics

For all statistical analyses, confirm that the following items are present in the figure legend, table legend, main text, or Methods section.

n/a	Confirmed	
☐	☒	The exact sample size (n) for each experimental group/condition, given as a discrete number and unit of measurement
☐	☒	A statement on whether measurements were taken from distinct samples or whether the same sample was measured repeatedly
☐	☒	The statistical test(s) used AND whether they are one- or two-sided<br>*Only common tests should be described solely by name; describe more complex techniques in the Methods section.*
☒	☐	A description of all covariates tested
☒	☐	A description of any assumptions or corrections, such as tests of normality and adjustment for multiple comparisons
☐	☒	A full description of the statistical parameters including central tendency (e.g. means) or other basic estimates (e.g. regression coefficient) AND variation (e.g. standard deviation) or associated estimates of uncertainty (e.g. confidence intervals)
☐	☒	For null hypothesis testing, the test statistic (e.g. F, t, r) with confidence intervals, effect sizes, degrees of freedom and P value noted<br>*Give P values as exact values whenever suitable.*
☒	☐	For Bayesian analysis, information on the choice of priors and Markov chain Monte Carlo settings
☒	☐	For hierarchical and complex designs, identification of the appropriate level for tests and full reporting of outcomes
☒	☐	Estimates of effect sizes (e.g. Cohen's d, Pearson's r), indicating how they were calculated

Our web collection on statistics for biologists contains articles on many of the points above.

Software and code

Policy information about availability of computer code

Data collection	CytoFLEX S, Agilent Novocyte Advanteon, and Attune NxT were used to collect flow cytometry data. The StepOnePlus Real-Time PCR System (Applied Biosystems), Tanon-5200 (Tanon Science & Technology), Thermo Fisher Talos L120C TEM, iCAP Qc instrument (Thermo Fisher Scientific), AniView100 Pro multimodal in vivo imaging system (BLT, China), BioComp Piston Gradient Fractionator (BioComp Instruments), and BioTek Synergy H1 microplate reader (Agilent Technologies) were used to collect qPCR, Western blotting, TEM, ICP-MS, in vivo imaging, polysome profiling, and absorbance data, respectively.
Data analysis	GraphPad Prism 8 (GraphPad Software) was used for statistical graph generation; FlowJo_v10.8 software (BD Life Sciences) was used for flow cytometry data analysis; Microsoft Excel was employed for primary data calculation and statistics; ImageJ (National Institutes of Health, NIH) was used for mitochondrial morphology analysis; and R (The R Foundation) was utilized for bioinformatics analysis. In addition, the proprietary software accompanying the aforementioned instruments was also applied for data acquisition and analysis.

For manuscripts utilizing custom algorithms or software that are central to the research but not yet described in published literature, software must be made available to editors and reviewers. We strongly encourage code deposition in a community repository (e.g. GitHub). See the Nature Portfolio guidelines for submitting code & software for further information.

Data

Policy information about availability of data

All manuscripts must include a data availability statement. This statement should provide the following information, where applicable:

- Accession codes, unique identifiers, or web links for publicly available datasets
- A description of any restrictions on data availability
- For clinical datasets or third party data, please ensure that the statement adheres to our policy

The accession codes for all publicly available datasets utilized in this study are as follows: Spatial transcriptomic data of 4T1 tumours (GSE230098); scRNA-seq data of Lung cancer (GSE127465), Colorectal cancer (EMTAB8107, GSE166555, GSE146771, GSE108989), Non-small cell lung cancer (GSE117570), Prostate adenocarcinoma (GSE137829, GSE141445, GSE176031), Ovarian cancer (GSE151214), Pancreatic adenocarcinoma (GSE148673, GSE154778), Acute myeloid leukemia (GSE116256), Basal Cell Carcinoma (GSE123813), Hepatocellular Carcinoma (GSE140228), Melanoma (GSE115978), and Squamous Cell Carcinoma (GSE123813). The Pan-Cancer scRNA-seq dataset is available at http://cancer-pku.cn:3838/PanC_T/. All other data supporting the findings of this study are available from the corresponding author. Source data are provided with this paper.

Research involving human participants, their data, or biological material

Policy information about studies with human participants or human data. See also policy information about sex, gender (identity/presentation), and sexual orientation and race, ethnicity and racism.

Reporting on sex and gender	The sex of patients from whom tumor tissues, blood samples, and malignant pleural effusions were collected is documented in Supplementary Table 1. As the study was not powered to detect sex-based differences, this variable was not considered as a covariate in our analytical models.
Reporting on race, ethnicity, or other socially relevant groupings	All samples were collected from consecutively enrolled patients, irrespective of race, ethnicity, or other sociodemographic characteristics.
Population characteristics	The clinicopathological characteristics of the patient cohorts with lung cancer and colorectal cancer are summarized in Supplementary Table 1.
Recruitment	For any primary tumor, blood, or malignant pleural effusion samples prospectively collected by our institution, written informed consent was obtained from all patients prior to collection. No participants received compensation for this study, in accordance with our institutional guidelines. Patients were enrolled consecutively to avoid selection bias. Regarding the transcriptomic data (including RNA-seq, spatial transcriptomic, and single-cell RNA-seq data) used in this study, all were sourced from public repositories (GEO and TCGA databases). As we utilized only pre-existing, de-identified public data, we were not involved in patient recruitment or original data collection for these datasets, and thus separate ethical approval was not required for their use.
Ethics oversight	The study was approved by the Institutional Review Boards of the Third Affiliated Hospital of Sun Yat-sen University.

Note that full information on the approval of the study protocol must also be provided in the manuscript.

Field-specific reporting

Please select the one below that is the best fit for your research. If you are not sure, read the appropriate sections before making your selection.

☒ Life sciences ☐ Behavioural & social sciences ☐ Ecological, evolutionary & environmental sciences

For a reference copy of the document with all sections, see nature.com/documents/nr-reporting-summary-flat.pdf

Life sciences study design

All studies must disclose on these points even when the disclosure is negative.

Sample size	The sample size for each experiment is indicated in figures or figure legends. The sample sizes were not predetermined by statistical tools. The sample or group sizes of the experiments were chosen based on previous experience in the lab.
Data exclusions	No samples or animals were excluded from the analysis.
Replication	Multiple independent repeats were included for related experiments. Each experiment was performed at least three times to make sure similar results are reproducible. All attempts at replication were successful.
Randomization	Mice or cells were randomly allocated to experimental groups.
Blinding	For animal study, mice were given a number prior to data collection and analysis. Data was collected and analyzed blindly. For in vitro experiments, investigators were not blinded, as standard in this manner of study, which contained multiple steps requiring distinct operations for accuracy and precision precluding blinding to experimental variables.

Reporting for specific materials, systems and methods

We require information from authors about some types of materials, experimental systems and methods used in many studies. Here, indicate whether each material, system or method listed is relevant to your study. If you are not sure if a list item applies to your research, read the appropriate section before selecting a response.

Materials & experimental systems

n/a	Involved in the study
☐	☒ Antibodies
☐	☒ Eukaryotic cell lines
☒	☐ Palaeontology and archaeology
☐	☒ Animals and other organisms
☒	☐ Clinical data
☒	☐ Dual use research of concern
☒	☐ Plants

Methods

n/a	Involved in the study
☒	☐ ChIP-seq
☐	☒ Flow cytometry
☒	☐ MRI-based neuroimaging

Antibodies

Antibodies used	TfR1 (1:1000, Invitrogen, 13-6800), DMT1 (1:1000, Abcam, ab55735), FTH1 (1:1000, Cell Signaling Technology, 4393), FTL (1:1000, Proteintech, 10727-1-AP), SLC40A1 (1:1000, Novus, NBP1-21502), HAMP1 (1:500, Abcam, ab190775), Caspase-3 (1:1000, Cell Signaling Technology, 9662S), Cleaved Caspase-3 (1:1000, Cell Signaling Technology, 9664T), ACSL3 (1:1000, Abclonal, A22085), FSP1 (1:1000, Proteintech, 68049), SLC7A11 (1:1000, Cell Signaling Technology, 98051S), GPX4 (1:1000, Cell Signaling Technology, 59735T), β-actin (1:5000, RayBiotech, RM2001), GAPDH (1:5000, RayBiotech, RM2002), mouse IgG (1:10000, RayBiotech, RM3001), and rabbit IgG (1:10000, RayBiotech, RM3002). FVD506 (65-0866-14), FITC anti-mouse TCR β (11-5961-82), eF450 anti-mouse CD90.2 (48-0902-82), eF506 anti-mouse CD4 (69-0042-82), SB600 anti-mouse CD8a (63-0081-82), APC anti-mouse PD-1 (17-9985-82), eF710 anti-mouse TIM3 (46-5870-82), PE-Cy7 anti-mouse LAG-3 (25-2231-82), eF450 anti-mouse IFN-γ (48-7311-82), APC anti-mouse IFN-γ (17-7311-82), PerCP Cy5.5 anti-mouse IL-2 (45-7021-82), PE-Cy7 anti-mouse GranzymeB (25-8898-82), PE anti-mouse TfR1 (12-0711-81), APC anti-mouse CD25 (17-0251-82), SB600 anti-mouse CD69 (63-0691-82), PE anti-human TfR1 (12-0719-42), PerCP eF710 anti-human TIM3 (46-3109-42), and APC anti-human CD4 (17-0047-42) (both from eBioscience); FVS700 (564997), 7-AAD (559925), APC-Cy7 anti-mouse CD45 (557659), APC anti-mouse CD4 (553051), PE anti-mouse TNF (554419), BV650 anti-Ki-67 (563757), BV421 anti-mouse CD25 (562606), PE-Cy7 anti-mouse CD11b (552850), PE anti-mouse F4/80 (565410), APC anti-mouse NK-1.1 (550627), BV605 anti-mouse CD11c (563057), V500 anti-mouse I-A/I-E (562366), anti-mouse GPX4 (MA5-32827), AF488 goat anti-rabbit (SA5-10384-AFP488), BV421 anti-human PD-1 (564323), APC Cy7 anti-human CD3 (557832), and BV510 anti-human CD8 (743065) (both from BD Biosciences); FITC anti human/mouse SLC40A1 (Novus, NBP2-75923F).
Validation	All antibodies and stains are commercially available and were validated on the manufacturer's website.

Eukaryotic cell lines

Policy information about cell lines and Sex and Gender in Research

Cell line source(s)	B16-F10, LLC, 4T1, NCI-H446, NCI-H1299, NCI-H1703, PC-9, HepG2 and HEK-293T cell lines were obtained from Newgainbio (Jiangsu, China). MC38 and ID8 cell lines were obtained from Cellverse (Shanghai, China).
Authentication	None of the cell lines used were authenticated.
Mycoplasma contamination	All cell lines were tested negative for mycoplasma contamination.
Commonly misidentified lines (See ICLAC register)	No commonly misidentified lines were used.

Animals and other research organisms

Policy information about studies involving animals; ARRIVE guidelines recommended for reporting animal research, and Sex and Gender in Research

Laboratory animals	C57BL/6J and BALB/c-nude male mice (7-week-old) were purchased from GemPharmatech Co., Ltd (Jiangsu, China). C57BL/6J female mice (7-week-old) were purchased from GemPharmatech Co., Ltd (Jiangsu, China). Sprague-Dawley (SD) rats (280-300 g) were obtained from Slike Jingda Laboratory Animal Co., Ltd (Hunan, China).
Wild animals	No wild animals involved in this study.
Reporting on sex	Mouse experiments utilized female C57BL/6 mice for the ID-8 ovarian cancer model, and both sexes for other studies without specific sex-based considerations.
Field-collected samples	This study didn't involve field-collected samples.

Ethics oversight | All mouse experiments were approved by the Institutional Animal Care and Use Committee (IACUC) of Guangzhou National Laboratory, GZLAB-AUCP-2024-04-A02.

Note that full information on the approval of the study protocol must also be provided in the manuscript.

Plants

Seed stocks	*Report on the source of all seed stocks or other plant material used. If applicable, state the seed stock centre and catalogue number. If plant specimens were collected from the field, describe the collection location, date and sampling procedures.*
Novel plant genotypes	*Describe the methods by which all novel plant genotypes were produced. This includes those generated by transgenic approaches, gene editing, chemical/radiation-based mutagenesis and hybridization. For transgenic lines, describe the transformation method, the number of independent lines analyzed and the generation upon which experiments were performed. For gene-edited lines, describe the editor used, the endogenous sequence targeted for editing, the targeting guide RNA sequence (if applicable) and how the editor was applied.*
Authentication	*Describe any authentication procedures for each seed stock used or novel genotype generated. Describe any experiments used to assess the effect of a mutation and, where applicable, how potential secondary effects (e.g. second site T-DNA insertions, mosiacism, off-target gene editing) were examined.*

Flow Cytometry

Plots

Confirm that:

☒ The axis labels state the marker and fluorochrome used (e.g. CD4-FITC).

☒ The axis scales are clearly visible. Include numbers along axes only for bottom left plot of group (a 'group' is an analysis of identical markers).

☒ All plots are contour plots with outliers or pseudocolor plots.

☒ A numerical value for number of cells or percentage (with statistics) is provided.

Methodology

Sample preparation	Spleen and lymph nodes were mechanically dissociated through 100-μm cell strainers (Falcon, 352360) in MACS buffer (PBS + 2 mM EDTA + 1% FBS). Erythrocytes were lysed using RBC Lysis Buffer (Yuanye Bio-Technology, R20176) for 2 min at room temperature (RT), and the reaction was stopped by adding a 10 × volume of ice-cold MACS buffer. For tumor tissues, specimens were minced into fragments smaller than 1 mm3 in RPMI-1640 complete medium supplemented with 0.1 mg/mL DNase I (HARVEYBIO, EZ1179), 1 mg/mL Collagenase Type II (Sigma-Aldrich, V900892), 1 mg/mL Collagenase Type IV (Sigma-Aldrich, V900893), and 2.5 μg/mL Hyaluronidase (Sigma-Aldrich, H3506). The mixtures were then digested for 30 min at 37°C with gentle agitation (200 rpm). After digestion, the slurry was filtered through 70-μm cell strainers (Falcon, 352350) and further dissociated using a syringe plunger. Finally, cells were washed with MACS buffer and pelleted by centrifugation at 500 × g for 5 min to obtain single-cell suspensions. For surface marker staining, single-cell suspensions were incubated with fluorochrome-conjugated antibodies in MACS buffer for 15 min at RT in the dark. For intracellular cytokine detection, cells were first restimulated ex vivo in RPMI 1640 containing 10% FBS, PMA, and ionomycin in the presence of protein transport inhibitors (brefeldin A and monensin) for 4-6 h at 37°C under 5% CO2. Following stimulation, cells were stained for surface markers as described. For intracellular targets, two fixation and permeabilization procedures were used. For intracellular cytokine staining, cells were fixed overnight at 4°C using IC Fixation Buffer (eBioscience, 00-8222-49) and then permeabilized with 1 × Permeabilization Buffer (eBioscience, 00-8333-56). For transcription factor staining, cells were fixed overnight at 4°C with Foxp3/Transcription Factor Staining Buffer (eBioscience, 00-5523-00) and subsequently washed with the corresponding 1× permeabilization buffer. After permeabilization, cells were incubated with intracellular antibodies for 30 min at RT in the dark. For detection of the intracellular iron, cells were washed twice with Dulbecco's Phosphate-Buffered Saline (DPBS) and subsequently incubated with 1 μM FerroOrange (Dojindo, F374) or RhoNox-1 (MedChemExpress, HY-D1533) in serum-free medium MT101 for 30 min at 37°C in the dark. To assess lipid peroxidation, cells were washed twice with Hank's Balanced Salt Solution (HBSS) and then incubated with 1 × BDP 581/591 C11 (Dojindo, L267) or BODIPY™ 665/676 (Invitrogen, B3932) working solution for 30 min at 37°C in the dark. For total cellular ROS measurement, cells were washed twice with PBS and loaded with 1 μM CM-H2DCFDA (Invitrogen, C6827) in PBS for 30 min at 37°C in the dark. Following two washes with PBS, cells were further incubated in complete growth medium for 30 min at 37°C to allow for complete intracellular de-esterification. After two final washes with PBS, oxidant-sensitive fluorescence was quantified by flow cytometry.
Instrument	CytoFLEX S (Beckman Coulter), Agilent Novocyte Advanteon (Agilent Technologies), and Attune NxT (Thermo Fisher Scientific).
Software	Using CytExpert software, NovoExpress software, and Attune NxT Cytometer software to collect data and FlowJo_v10.8 software to analyze data.
Cell population abundance	At least 10,000 cells were analyzed for each sample.

Gating strategy

The flow cytometry gating strategy began by selecting the main cell population based on FSC and SSC, followed by the isolation of single cells using FSC-H vs. FSC-A to exclude doublets. Live cells were then identified by negativity for FVS700 (or 7-AAD). CD45-positive immune cells were gated from the live cells, followed by the identification of T cells using CD3 (or Thy1.2). Finally, T cell subsets were resolved by staining for CD4 and CD8.

☒ Tick this box to confirm that a figure exemplifying the gating strategy is provided in the Supplementary Information.
