# Supplementary material for: Iron overload in the tumor microenvironment induces CD8+ T cell ferroptosis and dysfunction
Source: Nat Commun. 2026 May 22;17:6754. doi: 10.1038/s41467-026-73379-4 (PMC13385831; doi:10.1038/s41467-026-73379-4)
Supplement: Supplementary file 3 — Transparent Peer Review file [file 41467_2026_73379_MOESM3_ESM.pdf]

nature portfolio

Peer Review File

Iron overload in the tumor microenvironment induces CD8+ T cell ferroptosis and dysfunction

Corresponding Author: Dr Yingjie Bian

This file contains all reviewer reports in order by version, followed by all author rebuttals in order by version.

Version 0:

Reviewer comments:

Reviewer #1

(Remarks to the Author)
This manuscript explores how excess iron within the tumor microenvironment promotes ferroptosis and functional exhaustion of $CD8^+$ T cells through suppression of the iron exporter SLC40A1—a process the authors link to chronic TCR stimulation and tumor-derived hepcidin. The study also introduces SLC40A1-overexpressing CAR-T cells as a potential strategy to improve T-cell fitness in an iron-rich TME.
The topic is timely and relevant, and the authors make good use of patient samples, mouse models, and in vitro systems to connect intratumoral iron overload with compromised T-cell immunity.
That said, several important conceptual and mechanistic issues remain unresolved. In several places the conclusions extend beyond what the data directly support, and key aspects of selective ferroptotic susceptibility among immune and tumor cell populations require deeper investigation. In addition, the CAR-T section, while intriguing, currently occupies a disproportionate amount of space compared with the central mechanistic story and would benefit from tighter integration.

Major Comments
1. The title and overall framing imply a broad immune regulatory mechanism, yet the experimental work focuses almost exclusively on $CD8^+$ T cells. Other iron-responsive immune populations—such as $CD4^+$ Th1 cells, Tregs, NK cells, dendritic cells, and macrophages—are only briefly examined for iron loading and not functionally assessed. Because TAMs, DCs, and Tregs play major roles in iron handling and immune suppression in tumors, limiting the mechanistic analysis to $CD8^+$ T cells weakens the broader claim of a pan-immune phenomenon.
2. The data clearly show that $CD8^+$ T cells succumb to iron-induced death at doses tolerated by tumor cells, but the reason for this difference is left unexplained. This is a significant conceptual gap. Tumor cells generally reinforce multiple ferroptosis-protective pathways (GPX4, FSP1, CoQ_{10} synthesis, ferritin, and MUFA-rich lipid remodeling), whereas T cells have a very different metabolic and lipid architecture. Without examining these pathways directly, the basis for selective vulnerability remains speculative. In addition, it is unclear whether this disparity reflects intrinsic biology or is influenced by the particular cancer cell lines and tumor models chosen for the study.
3. Although the authors show that hepcidin can degrade SLC40A1 in T cells in vitro, several questions remain. It is not established whether hepcidin concentrations in tumor interstitial fluid are high enough to modulate TIL biology. The relative contributions of tumor-derived versus host-derived hepcidin also remain undefined. Most importantly, the requirement for hepcidin in driving TIL ferroptosis has not been tested—its sufficiency is shown, but its necessity is not.
4. The CAR-T section is interesting and potentially useful, but in its current form it reads like an independent study attached to the end of the manuscript. The mechanistic bridge between endogenous TIL biology and engineered CAR-T cells is not fully developed, and the length of this section feels disproportionate to its connection with the central iron-SLC40A1 pathway.
5. Measuring total iron in lymph, blood, and tumor tissue is informative, but several critical issues remain unresolved. It is not clear how labile (redox-active) iron differs across compartments. Nor is it shown that these differences dictate T-cell activation thresholds or the trajectory toward exhaustion. Another open question is whether simply moving T cells from a low-iron environment into a high-iron environment is sufficient to trigger ferroptosis without additional TCR signals. As presented, the data demonstrate correlation but fall short of establishing causation.
6. The manuscript highlights iron accumulation in necrotic tumor regions but does not examine ferroptotic markers in tumor cells in situ—such as lipid peroxidation, GPX4 expression, or other ferroptosis signatures. This point is important, because if tumor cells in these high-iron areas are also undergoing ferroptosis, the argument that iron enrichment serves primarily as an immune-evasion mechanism becomes less compelling.

7. Although ferroptosis is a popular focus in current research, the contribution of apoptosis—an established and well-characterized cell-death pathway—should not be overlooked. The manuscript would benefit from clarifying whether and how apoptotic mechanisms contribute to the observed T-cell dysfunction, as prior findings in this area cannot simply be dismissed.

Reviewer #2

(Remarks to the Author)
In this study, the authors study the impact of iron on CD8 T cells. They established that iron is elevated in the TME compared to serum or lymph from the same patients. This high level of iron is triggering ferroptosis in CD8 T cells that express low level of SLC40A1 and cannot export iron. The authors used both SLC40A1 overexpression and iron chelation and showed they could limit ferroptosis and restore some cytotoxic activities in CD8 T cells. They also used a similar strategy in CAR T cells, leading to slightly increased tumor control. The study is interesting, but some conclusions are overstated. The authors need to address some points before publication.
One point is to discuss a recent publication suggesting the opposite effect of iron on T cell response and immunotherapy (doi: 10.1158/2326-6066.CIR-23-0739).
The authors should also be careful with the use of the term exhaustion. They never stained for TOX in their T cells and use many in vitro assays where they qualified activated T cells as exhausted. It is also important that the authors show flow cytometry dot plots and not only bar graphs. Gating strategies are not displayed in any supplemental figures.
Figure 2: In vitro, the use of the term exhaustion is not appropriate. The presence of high level of iron decreases their capacity to produce cytokines. In vivo, the authors showed that CD8 T cells are undergoing ferroptosis. There is nothing establishing the link between ferroptosis and exhaustion unless the authors can restore TILs function in vivo/ex-vivo with ferroptosis inhibitor.
Figure 3: the authors states that chronic TCR stimulation drives SLC40A1 downregulation. However, in figure 3G, slc40a1 RNA is already much lower at the first re-stimulation. Could the author add the level for the same mRNA for T cells only stimulated once? It would be helpful to have a direct comparison. Furthermore, the in vitro model used should be termed “chronic stimulation” model. As the author shows, exhaustion induction is more complex than just TCR triggering. The overexpression experiment is nice, but would be more interesting in vivo.
Figure 4: In figure 3, the authors concluded that chronic TCR triggering was sufficient to downregulate SLC40A1. There was no hepcidin in the chronic stimulation model. I don’t see the logic to investigate hepcidin then. Furthermore, the functional effects of HEP-25, although statistically relevant, are minor (TNF, PD-1, TIM-3). Similarly, the effect of overexpression on tumor growth is minor. Maybe a knock down of hepcidin expression in the tumor would be more appropriate.
Figure 5: I didn’t find any mention about the time point when the ex-vivo stimulations are performed for assessing cytokine production in TILs. In figure 5b, HID induces faster tumor progression. If cytokine assessment is performed at the end of the experiment, the size of the tumor, rather than the iron content, could be the main factor of T cell exhaustion. The authors should perform this analysis when the tumors have similar sizes. Same remark for the DFP treatment in both B16 and ID8. As both treatments could impact directly tumor growth, the author must perform similar experiments in immunodeficient animals to prove that the main effect is through T cells. So far, they have not proved it.
Figure 6: The data are interesting, although the effect on tumor growth remains quite limited.

Version 1:

Reviewer comments:

Reviewer #1

(Remarks to the Author)

Overall, the authors have substantially improved the manuscript and addressed most of my major concerns through additional experiments and more cautious interpretation. One remaining concern is that, although the role of tumor-derived hepcidin has been strengthened, the relative contribution of host-derived hepcidin and the local hepcidin concentrations within the tumor microenvironment remain insufficiently defined.

Reviewer #2

(Remarks to the Author)
I am satisfied with the answers from the authors. I congratulate them for their nice study.

Open Access This Peer Review File is licensed under a Creative Commons Attribution 4.0 International License, which permits use, sharing, adaptation, distribution and reproduction in any medium or format, as long as you give appropriate credit to the original author(s) and the source, provide a link to the Creative Commons license, and indicate if changes were made.
In cases where reviewers are anonymous, credit should be given to 'Anonymous Referee' and the source.
The images or other third party material in this Peer Review File are included in the article's Creative Commons license, unless indicated otherwise in a credit line to the material. If material is not included in the article's Creative Commons license and your intended use is not permitted by statutory regulation or exceeds the permitted use, you will need to obtain permission directly from the copyright holder.
To view a copy of this license, visit https://creativecommons.org/licenses/by/4.0/

REVIEWER COMMENTS

REVIEWER 1:

This manuscript explores how excess iron within the tumor microenvironment promotes ferroptosis and functional exhaustion of $CD8^+$ T cells through suppression of the iron exporter SLC40A1 - a process the authors link to chronic TCR stimulation and tumor-derived hepcidin. The study also introduces SLC40A1-overexpressing CAR-T cells as a potential strategy to improve T-cell fitness in an iron-rich TME.

The topic is timely and relevant, and the authors make good use of patient samples, mouse models, and in vitro systems to connect intratumoral iron overload with compromised T-cell immunity.

That said, several important conceptual and mechanistic issues remain unresolved. In several places the conclusions extend beyond what the data directly support, and key aspects of selective ferroptotic susceptibility among immune and tumor cell populations require deeper investigation. In addition, the CAR-T section, while intriguing, currently occupies a disproportionate amount of space compared with the central mechanistic story and would benefit from tighter integration.

Response: We sincerely thank the reviewer for the constructive summary and positive evaluation of our study. We are encouraged by the reviewer's recognition of the timeliness and relevance of our work, as well as the use of complementary experimental systems. We have carefully considered all raised concerns and have performed additional experiments and text revisions to strengthen the mechanistic support, clarify the conclusions, and better integrate the CAR-T section with the main story. Below, we provide detailed point-by-point responses to each specific comment.

1. The title and overall framing imply a broad immune regulatory mechanism, yet the experimental work focuses almost exclusively on $CD8^+$ T cells. Other iron-responsive immune populations-such as $CD4^+$ Th1 cells, Tregs, NK cells, dendritic cells, and macrophages-are only briefly examined for iron loading and not functionally assessed. Because TAMs, DCs, and Tregs play major roles in iron handling and immune suppression in tumors, limiting the mechanistic analysis to $CD8^+$ T cells weakens the broader claim of a pan-immune phenomenon.

Response: We thank the reviewer for raising this important point and agree that a comprehensive analysis of iron's impact across multiple immune subsets would be valuable. To address this specifically, we conducted further experiments and analyses, which reinforce our rationale for focusing primarily on $CD8^+$ T cells:

First, we performed regional analysis in the LLC tumor model by separately collecting tissues from necrotic and non-necrotic areas. While T cells were markedly reduced in necrotic areas, the distribution of macrophages, dendritic cells, and NK cells showed no significant difference (Extended Data Fig. 1h). Further analysis revealed that within T cells of necrotic regions, tumor-infiltrating $CD8^+$ T cells exhibited pronounced iron

accumulation and cell death, whereas $CD4^+$ T cells did not (Fig. 1k and Extended Data Fig. 1i).

Second, spatial transcriptomic analysis of tumors revealed that among major immune populations - including $CD8^+$ T cells, $CD4^+$ T cells, Tregs, macrophages, dendritic cells, and NK cells - $CD8^+$ T cells showed pronounced and consistent exclusion from iron-rich necrotic regions, but not other cell types (Fig. 1l and Extended Data Fig. 1j).

Third, in high-iron diet tumor models, we observed a reduction in $CD8^+$ T cell infiltration, whereas $CD4^+$ T cell numbers were even increased, and no significant changes were observed in macrophages, dendritic cells, or NK cells (Fig. 5f and Extended Data Fig. 6e).

Together, these data indicate that $CD8^+$ T cells exhibit a vulnerability to iron-rich necrotic niches, which led us to prioritize them for mechanistic investigation. In response to the reviewer's comment, we have toned down broader claims in the title and text to emphasize that our findings delineate a $CD8^+$ T cell-focused mechanism within the iron-loaded TME.

2. The data clearly show that $CD8^+$ T cells succumb to iron-induced death at doses tolerated by tumor cells, but the reason for this difference is left unexplained. This is a significant conceptual gap. Tumor cells generally reinforce multiple ferroptosis-protective pathways (GPX4, FSP1, CoQ_{10} synthesis, ferritin, and MUFA-rich lipid remodeling), whereas T cells have a very different metabolic and lipid architecture. Without examining these pathways directly, the basis for selective vulnerability remains speculative. In addition, it is unclear whether this disparity reflects intrinsic biology or is influenced by the particular cancer cell lines and tumor models chosen for the study.

Response: We thank the reviewer for raising this critical point and agree that elucidating the mechanistic basis for the differential ferroptosis sensitivity is essential for a complete understanding of our findings. To address this, we performed a systematic, multi-level comparison between $CD8^+$ T cells and various tumor cell lines (including human and murine, spanning multiple cancer types).

First, we analyzed the expression of key ferroptosis-protective genes and pathways highlighted by the reviewer. Using bioinformatics, qPCR, and western blotting, we consistently found that tumor cells express significantly higher levels of GPX4, FSP1, SLC7A11, ACSL3, and ferritin compared to $CD8^+$ T cells (Extended Data Fig. 3a-g, o).

Second, to functionally validate these observations, we treated both cell types with classical ferroptosis inducers. $CD8^+$ T cells exhibited markedly higher sensitivity to ferroptosis than all tested tumor cell lines, confirming their heightened sensitivity to ferroptosis (Extended Data Fig. 3h-k).

Together, these results indicate that the selective vulnerability of $CD8^+$ T cells is linked to their intrinsically lower expression of multiple ferroptosis-protective mechanisms, a

difference that appears to be conserved across species and tumor models. We have incorporated these new data into the revised manuscript and have expanded the discussion to clarify how the distinct metabolic and lipid architectures of T cells and tumor cells contribute to this disparity (Page 31, Line 833-836).

3. Although the authors show that hepcidin can degrade SLC40A1 in T cells in vitro, several questions remain. It is not established whether hepcidin concentrations in tumor interstitial fluid are high enough to modulate TIL biology. The relative contributions of tumor-derived versus host-derived hepcidin also remain undefined. Most importantly, the requirement for hepcidin in driving TIL ferroptosis has not been tested-its sufficiency is shown, but its necessity is not.

Response: We thank the reviewer for these insightful questions, which have helped us significantly strengthen the *in vivo* relevance and mechanistic understanding of hepcidin in our study.

To directly test the necessity of tumor-derived hepcidin, we generated hepcidin-knockout B16-F10 and LLC tumor cells (Extended Data Fig. 5j). In subsequent *in vivo* tumor implantation experiments, tumors lacking hepcidin exhibited significantly attenuated growth (Fig. 4h, i, and Extended Data Fig. 5k). Analysis of the tumor immune infiltrate revealed a marked increase in $CD8^+$ T cell accumulation in hepcidin-knockout tumors (Fig. 4j and Extended Data Fig. 5l). These $CD8^+$ T cells displayed higher SLC40A1 expression, reduced ferroptosis markers, and enhanced functional capacity compared to those from control tumors (Fig. 4k-o and Extended Data Fig. 5m). This suggests that tumor-derived hepcidin is necessary to disrupt $CD8^+$ T cell iron homeostasis and promote ferroptosis.

4. The CAR-T section is interesting and potentially useful, but in its current form it reads like an independent study attached to the end of the manuscript. The mechanistic bridge between endogenous TIL biology and engineered CAR-T cells is not fully developed, and the length of this section feels disproportionate to its connection with the central iron-SLC40A1 pathway.

Response: We thank the reviewer for this valuable point. To strengthen the connection between our central findings and CAR-T cell therapy, we performed additional experiments linking CAR-T cell dysfunction to the iron-SLC40A1-ferroptosis axis. First, we found that CAR-T cells infiltrating solid tumors similarly downregulate SLC40A1 and undergo ferroptosis in the iron-rich TME, mirroring the phenotype of endogenous $CD8^+$ TILs (Fig. 6a-e). Second, ferroptosis inhibitor Fer-1 treatment significantly enhanced CAR-T cell efficacy, demonstrating that ferroptosis inhibition represents a viable strategy to improve CAR-T therapy (Fig. 6f-i). Building on these insights, we engineered SLC40A1-overexpressing CAR-T cells, which exhibited marked resistance to ferroptosis and superior anti-tumor efficacy (Fig. 6 and Extended Data Fig. 7). Together, these findings bridge the gap between the

mechanistic understanding of iron-induced T cell ferroptosis and a rationally designed CAR-T cell therapy.

5. Measuring total iron in lymph, blood, and tumor tissue is informative, but several critical issues remain unresolved. It is not clear how labile (redox-active) iron differs across compartments. Nor is it shown that these differences dictate T-cell activation thresholds or the trajectory toward exhaustion. Another open question is whether simply moving T cells from a low-iron environment into a high-iron environment is sufficient to trigger ferroptosis without additional TCR signals. As presented, the data demonstrate correlation but fall short of establishing causation.

Response: We thank the reviewer for these essential questions, which have allowed us to strengthen the biochemical and functional evidence linking iron speciation to T cell fate. As suggested, we performed the following experiments:

1) Quantification of redox-active (labile) iron. We directly measured labile (redox-active) iron and found that its level differs across compartments. The data confirm that the tumor interstitial fluid contains a several-fold higher concentration of labile Fe^{2+} compared to lymph or serum (Extended Data Fig. 1l, m). Furthermore, using the Fe^{2+} probe FerroOrange, we directly visualized elevated labile iron specifically in tumor-infiltrating $CD8^+$ T cells (Fig. 1p, u, and Extended Data Fig. 1n), which correlated with exacerbated cell death and the upregulation of exhaustion markers (Fig. 1q-t, v-y and Extended Data Fig. 1o, p). These results substantiate that the tumor microenvironment is enriched in redox-active iron that is directly encountered by TILs.

2) Causality between iron exposure, TCR signaling, and ferroptosis: To address whether iron exposure alone is sufficient, we transferred $CD8^+$ T cells from a low-iron to a high-iron environment in the absence of TCR stimulation. This transfer alone was capable of triggering ferroptosis (Extended Data Fig. 4i), confirming that elevated extracellular iron can directly drive this death pathway. However, concurrent TCR signaling dramatically amplified iron-induced ferroptosis (Extended Data Fig. 4j).

Together, these data establish that the high labile iron in the TME directly overloads T cells. Chronic antigen exposure then drives TCR-mediated downregulation of SLC40A1, causing the T cells to lose iron export capacity, thereby triggering ferroptosis and functional impairment.

6. The manuscript highlights iron accumulation in necrotic tumor regions but does not examine ferroptotic markers in tumor cells in situ - such as lipid peroxidation, GPX4 expression, or other ferroptosis signatures. This point is important, because if tumor cells in these high-iron areas are also undergoing ferroptosis, the argument that iron enrichment serves primarily as an immune-evasion mechanism becomes less compelling.

Response: We thank the reviewer for raising this critical point. To directly address whether tumor cells in high-iron, necrotic regions undergo ferroptosis, we performed

a comparative analysis of CD45-negative tumor cells and $CD8^+$ T cells from necrotic versus non-necrotic areas within the same tumors.

Our data revealed that tumor cells in necrotic areas significantly upregulate GPX4 expression and are resistant to ferroptosis (Extended Data Fig. 3l, m). In contrast, $CD8^+$ T cells exhibit no difference in GPX4 levels between these regions, maintain significantly lower GPX4 than tumor cells, and consequently display higher lipid peroxidation and ferroptosis susceptibility (Extended Data Fig. 3l, 3n). These results are consistent with the earlier comparative experiments in "Comment #2", which showed that tumor cells possess substantially higher intrinsic resistance to iron overload and ferroptosis induction than $CD8^+$ T cells.

7. Although ferroptosis is a popular focus in current research, the contribution of apoptosis-an established and well-characterized cell-death pathway-should not be overlooked. The manuscript would benefit from clarifying whether and how apoptotic mechanisms contribute to the observed T-cell dysfunction, as prior findings in this area cannot simply be dismissed.

Response: We thank the reviewer for raising this important point. We fully agree that the potential role of apoptosis, a classical and well-characterized cell death pathway, should be carefully evaluated alongside ferroptosis.

To directly assess whether apoptosis contributes to the iron-induced $CD8^+$ T cell dysfunction observed in our study, we performed a series of complementary experiments. First, we examined cleaved caspase-3 levels by western blotting in $CD8^+$ T cells following iron challenge. While a baseline of apoptotic activity was detectable in control $CD8^+$ T cells, iron treatment did not elevate cleaved caspase-3 expression, suggesting that iron-induced death does not proceed through caspase-3-dependent apoptosis (Extended Data Fig. 2n).

To functionally distinguish the two pathways, we then co-treated $CD8^+$ T cells with iron and either the pan-caspase inhibitor Z-VAD-FMK or the ferroptosis-specific inhibitor ferrostatin-1 (Fer-1). The apoptosis inhibitor Z-VAD-FMK failed to rescue iron-induced cell death and did not restore cytokine secretion capacity. In contrast, Fer-1 provided significant protection against cell death and preserved $CD8^+$ T cell function (Extended Data Fig. 2o, p).

Collectively, these data indicate that under our experimental conditions, iron-induced $CD8^+$T cell death and functional impairment are primarily driven by ferroptosis.

REVIEWER 2:

In this study, the authors study the impact of iron on CD8 T cells. They established that iron is elevated in the TME compared to serum or lymph from the same patients. This high level of iron is triggering ferroptosis in CD8 T cells that express low level of SLC40A1 and cannot export iron. The authors used both SLC40A1 overexpression and iron chelation and showed they could limit ferroptosis and restore some cytotoxic activities in CD8 T cells. They also used a similar strategy in CAR T cells, leading to slightly increased tumor control. The study is interesting, but some conclusions are overstated. The authors need to address some points before publication.

Response: We sincerely thank the reviewer for the constructive summary and overall positive assessment of our study. We agree that some conclusions in the original manuscript could be presented with greater precision. In response, we have carefully revised the text to ensure our claims are closely aligned with the experimental data, moderating any overstatements. We have also performed additional experiments and analyses to strengthen the mechanistic support and clarify the biological implications. Below, we provide a detailed point-by-point response to each specific comment.

1. One point is to discuss a recent publication suggesting the opposite effect of iron on T cell response and immunotherapy (doi: 10.1158/2326-6066.CIR-23-0739).

Response: We thank the reviewer for bringing this relevant reference to our attention. We are aware of this recent study, and we believe several key methodological differences likely account for this discrepancy:

First, the nature, dosage, route, and timing of iron administration differ significantly between the studies. Our work models chronic, systemic iron overload via direct intragastric $FeSO_4$ gavage, focusing on its sustained impact within the tumor microenvironment and its selective effect on $CD8^+$ T cell fitness. In contrast, the cited study employed a specific regimen of high-iron diet supplementation alongside anti-PD-1 therapy, which may represent a distinct pharmacological and kinetic scenario[1]. Second, our data show that $CD8^+$ T cells require iron within an optimal range: moderate iron supports their function, whereas iron overload in the tumor microenvironment induces T cell dysfunction. Thus, iron exerts dual, concentration-dependent effects on anti-tumor immunity, and the outcomes of iron modulation likely depend on the baseline iron status, treatment duration, and therapeutic context.

We have integrated this comparison into the revised “Discussion” to cite this work and clarify the contextual differences (Page 30, Line 818-826).

2. The authors should also be careful with the use of the term exhaustion. They never stained for TOX in their T cells and use many in vitro assays where they qualified activated T cells as exhausted. It is also important that the authors show flow cytometry dot plots and not only bar graphs. Gating strategies are not displayed in any supplemental figures.

Response: We thank the reviewer for this critical comment. We agree that the term "exhaustion" denotes a specific differentiation state and typically associated with markers like TOX. Accordingly, we have replaced "exhaustion" with the more accurate term "dysfunction" throughout the manuscript.

All relevant flow cytometry gating strategies and representative pseudocolor plots have now been provided in the "Source Data: Flow cytometry gating strategies and representative plots" to ensure full transparency and reproducibility.

3. Figure 2: In vitro, the use of the term exhaustion is not appropriate. The presence of high level of iron decreases their capacity to produce cytokines. In vivo, the authors showed that CD8 T cells are undergoing ferroptosis. There is nothing establishing the link between ferroptosis and exhaustion unless the authors can restore TILs function in vivo/ex-vivo with ferroptosis inhibitor.

Response: We thank the reviewer for these insightful suggestions. We have replaced "exhaustion" with the more accurate term "dysfunction". We agree that directly demonstrating that ferroptosis inhibition can restore the function of tumor-infiltrating $CD8^+$ T cells is crucial for establishing a functional link between the two processes.

To address this, we performed the following key experiment:

We pre-treated CAR-T cells with the ferroptosis inhibitor Ferrostatin-1 (Fer-1) prior to adoptive transfer into tumor-bearing mice. The Fer-1-pre-treated group exhibited significantly enhanced anti-tumor efficacy (Fig. 6f, g). Analysis of tumor-infiltrating CAR-T cells revealed increased infiltration and downregulated expression of exhaustion markers compared to the control group (Fig. 6h, i).

These data provide direct experimental evidence that inhibiting ferroptosis is sufficient to improve the survival and function of $CD8^+$ T cells in the iron-rich TME, thereby establishing a functional connection between ferroptosis and the observed T cell impairment in vivo. We have incorporated these findings into the revised manuscript (Fig. 6f-i).

4. Figure 3: the authors states that chronic TCR stimulation drives SLC40A1 downregulation. However, in figure 3G, slc40a1 RNA is already much lower at the first re-stimulation. Could the author add the level for the same mRNA for T cells only stimulated once? It would be helpful to have a direct comparison. Furthermore, the in vitro model used should be termed "chronic stimulation" model. As the author shows, exhaustion induction is more complex than just TCR triggering. The overexpression experiment is nice, but would be more interesting in vivo.

Response: We thank the reviewer for these constructive suggestions.

As suggested, we have now performed additional control experiments in which mouse $CD8^+$ T cells were stimulated once as control. qPCR analysis confirmed that *Slc40a1* mRNA levels remained stable in these cells over time, in contrast to the progressive downregulation observed upon chronic TCR stimulation (Extended Data

Fig. 4g). This conserved pattern was also validated in human $CD8^+$ T cells (Extended Data Fig. 4o).

We fully agree with the reviewer and have revised the manuscript to consistently refer to our *in vitro* system as a "chronic TCR stimulation model," which more accurately describes the experimental design.

We appreciate the reviewer's interest in the *in vivo* relevance of SLC40A1. In the current study, we chose to test the therapeutic potential of counteracting this pathway in a translational setting. Therefore, we engineered SLC40A1-overexpressing CAR-T cells and evaluated their function *in vivo*. This approach directly tests the hypothesis that bolstering iron export can enhance T cell resilience within the iron-rich TME. The results, presented in Figure 6 and Extended Figure 7, demonstrate that SLC40A1 overexpression improves CAR-T cell persistence and anti-tumor efficacy, providing *in vivo* proof-of-concept for the functional importance of this regulator.

5. Figure 4: In figure 3, the authors concluded that chronic TCR triggering was sufficient to downregulate SLC40A1. There was no hepcidin in the chronic stimulation model. I don't see the logic to investigate hepcidin then. Furthermore, the functional effects of HEP-25, although statistically relevant, are minor (TNF, PD-1, TIM-3). Similarly, the effect of overexpression on tumor growth is minor. Maybe a knock down of hepcidin expression in the tumor would be more appropriate.

Response: We thank the reviewer for these insightful comments, which have helped us clarify the rationale and strengthen the evidence for hepcidin's role.

We agree that our original description of the interplay between chronic TCR signaling and hepcidin was insufficiently clear. Our data indicate that these are two distinct but cooperating layers of regulation. Chronic TCR signaling intrinsically reprograms T cells, leading to transcriptional downregulation of SLC40A1 mRNA. Tumor-derived hepcidin acts post-translationally on the remaining SLC40A1 protein, accelerating its degradation. This logic is supported by our data showing that hepcidin induces more severe death in chronic TCR stimulated T cells (Fig. 4b-d) compared to resting cells (Extended Data Fig. 5d-f).

We acknowledge that the functional effects of the hepcidin peptide (HEP-25) in some of our in vitro assays, while statistically significant, were quantitatively modest. This may be attributable to the inherent instability of this short peptide hormone[2,3].

Most importantly, following the reviewer's suggestion, we directly tested the necessity of tumor-derived hepcidin *in vivo* by generating hepcidin-knockout B16 and LLC tumor cell lines (Extended Data Fig. 5j). Tumors lacking hepcidin exhibited markedly attenuated growth (Fig. 4h, i, and Extended Data Fig. 5k). Analysis of the TME revealed a significant increase in $CD8^+$ T cell infiltration (Fig. 4j, and Extended Data Fig. 5l). These $CD8^+$ T cells displayed higher SLC40A1 expression, reduced ferroptosis markers, and enhanced functional capacity compared to those from control tumors (Fig. 4k-o and Extended Data Fig. 5m).

These *in vivo* experiments provide strong, physiologically relevant evidence that tumor-derived hepcidin is a critical driver of $CD8^+$ T cell ferroptosis and dysfunction, with a major impact on tumor progression.

6. Figure 5: I didn't find any mention about the time point when the ex-vivo stimulations are performed for assessing cytokine production in TILs. In figure 5b, HID induces faster tumor progression. If cytokine assessment is performed at the end of the experiment, the size of the tumor, rather than the iron content, could be the main factor of T cell exhaustion. The authors should perform this analysis when the tumors have similar sizes. Same remark for the DFP treatment in both B16 and ID8. As both treatments could impact directly tumor growth, the author must perform similar experiments in immunodeficient animals to prove that the main effect is through T cells. So far, they have not proved it.

Response: We thank the reviewer for raising these critical methodological points, which are essential for accurately interpreting the functional data.

We agree that differences in terminal tumor size could confound the assessment of TIL function. To address this, we expanded the sample size and repeated the experiments in both the High-Iron Diet (HID) and Deferiprone (DFP) treatment models, focusing on mice with closely matched tumor sizes at the endpoint of analysis.

1) In HID models: Although HID-treated mice overall exhibited accelerated tumor growth and reduced $CD8^+$ T cell function compared to SID controls (Responding Figure 1a-f), a tumor size-matched analysis revealed that this dysfunction was independent of tumor burden (Responding Figure 1g-l). Specifically, when comparing the five smallest tumors from the HID group with the five largest tumors from the SID group to achieve comparable tumor sizes between conditions, we observed the same iron-induced $CD8^+$ T cell dysfunction (Responding Figure 1g-l).

Responding Figure 1

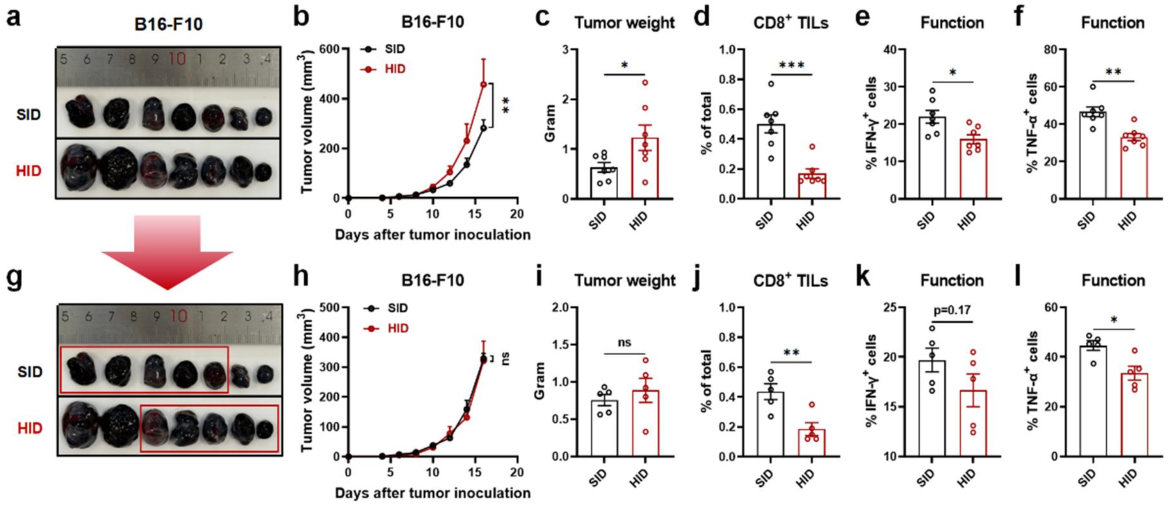

2) In DFP models: Similarly, DFP treatment significantly suppressed tumor growth and reduced ferroptosis and exhaustion markers in CD8$^+$ TILs (Responding Figure 2a-g). Tumor size-matched analysis comparing the seven smallest tumors from control group with the seven largest tumors from DFP-treated group confirmed the same protective effects on CD8$^+$ T cell function independent of tumor burden (Responding Figure 2h-n).

Responding Figure 2

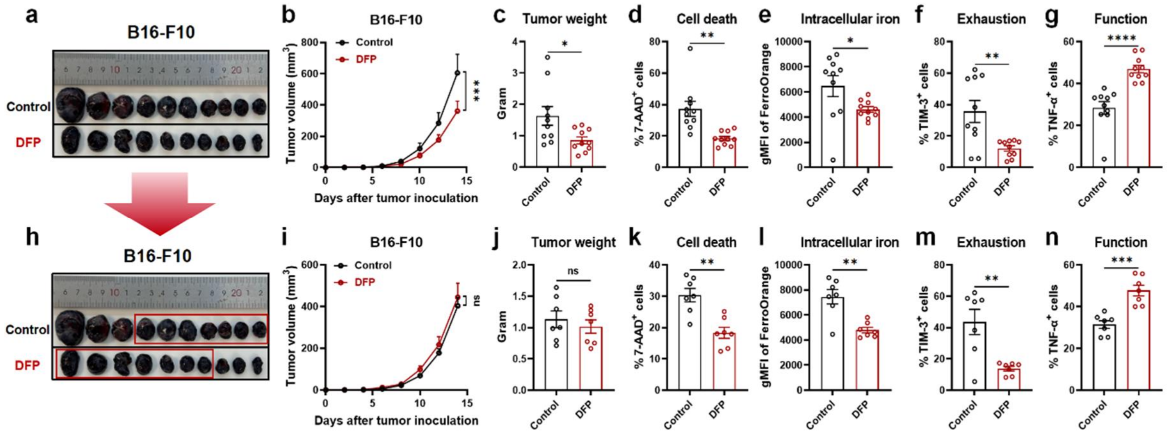

These findings confirm that the observed T cell dysfunction is directly linked to iron status, independent of gross tumor burden.

To definitively rule out the possibility that HID or DFP directly affect tumor growth independently of the immune system, we performed experiments in T cell-deficient nude mice. In this immunodeficient model, neither HID nor DFP treatment had a significant impact on the growth kinetics of B16 tumors (Extended Data Fig. 6b-d and 6i-k). This result provides direct evidence that the pro-tumor effect of iron overload and the anti-tumor effect of iron chelation are primarily mediated through their modulation of T cell function within the adaptive immune compartment.

These additional analyses and experiments have been incorporated into the revised manuscript and the corresponding Results section.

7. Figure 6: The data are interesting, although the effect on tumor growth remains quite limited.

Response: We thank the reviewer for this comment and acknowledge the moderate anti-tumor effect observed in our initial proof-of-concept experiments. This is likely because these experiments employed B16-F10 and LLC models, which are “cold” tumors with low baseline T-cell infiltration[4,5].

To directly address this, we performed experiment in a “hot” and immunogenic MC38-Trop2 model[4,5]. Here, SLC40A1-overexpressing CAR-T cells mediated a potent anti-tumor response, with reduced expression of exhaustion markers including PD-1, LAG-3, and TIM-3, compared to controls (Extended Data Fig. 7w-y).

These crucial data have been added to the manuscript.

References

1. Porte, S., *et al.* Iron Boosts Antitumor Type 1 T-cell Responses and Anti-PD1 Immunotherapy. *Cancer Immunol Res* **12**, 1252-1267 (2024).
2. Ruchala, P. & Nemeth, E. The pathophysiology and pharmacology of hepcidin. *Trends Pharmacol Sci* **35**, 155-161 (2014).
3. Rana, S. & Prabhakar, N. Iron disorders and hepcidin. *Clin Chim Acta* **523**, 454-468 (2021).
4. Shao, Q., *et al.* Tumor therapeutic response monitored by telemetric temperature sensing, a preclinical study on immunotherapy and chemotherapy. *Sci Rep* **13**, 7727 (2023).
5. Kienzl, M., *et al.* Comparative Study of the Immune Microenvironment in Heterotopic Tumor Models. *Cancers (Basel)* **16**(2024).

REVIEWERS' COMMENTS

Reviewer #1 (Remarks to the Author):

Overall, the authors have substantially improved the manuscript and addressed most of my major concerns through additional experiments and more cautious interpretation. One remaining concern is that, although the role of tumor-derived hepcidin has been strengthened, the relative contribution of host-derived hepcidin and the local hepcidin concentrations within the tumor microenvironment remain insufficiently defined.

Response: We thank the reviewer for the positive assessment and for raising this important point. We agree that the relative contribution of host-derived hepcidin and its local concentrations within the tumor microenvironment are interesting questions that warrant future investigation. We have now explicitly discussed this as a limitation of the current study in the Discussion section (Lines 562-570).

Reviewer #2 (Remarks to the Author):

I am satisfied with the answers from the authors. I congratulate them for their nice study.

Response: We appreciate the reviewer’s time and are grateful for the encouraging comments on our study.
